# Novel 9-Methylanthracene Derivatives as p53 Activators for the Treatment of Glioblastoma Multiforme

**DOI:** 10.3390/molecules29102396

**Published:** 2024-05-19

**Authors:** Yuxin Feng, Yingjie Wang, Xiaoxue Li, Ziqiang Sun, Sihan Qiang, Hongbo Wang, Yi Liu

**Affiliations:** 1 Key Laboratory of Molecular Pharmacology and Drug Evaluation, Ministry of Education, Yantai University, Yantai 264005, China; fengyuxinsx@126.com (Y.F.); 17865562075@163.com (Y.W.); 2 School of Chemistry and Chemical Engineering, Yantai University, Yantai 264005, China; lixiaoxue0829@163.com (X.L.); szq9805@163.com (Z.S.); qiangsihan0207@163.com (S.Q.)

**Keywords:** glioblastoma multiforme, anthracenyl skeleton, MDM4, antiproliferative, p53

## Abstract

Glioblastoma multiforme, a highly aggressive and lethal brain tumor, is a substantial clinical challenge and a focus of increasing concern globally. Hematological toxicity and drug resistance of first-line drugs underscore the necessity for new anti-glioma drug development. Here, 43 anthracenyl skeleton compounds as p53 activator **XI-011** analogs were designed, synthesized, and evaluated for their cytotoxic effects. Five compounds (**13d**, **13e**, **14a**, **14b**, and **14n**) exhibited good anti-glioma activity against U87 cells, with IC_50_ values lower than 2 μM. Notably, **13e** showed the best anti-glioma activity, with an IC_50_ value up to 0.53 μM, providing a promising lead compound for new anti-glioma drug development. Mechanistic analyses showed that **13e** suppressed the MDM4 protein expression, upregulated the p53 protein level, and induced cell cycle arrest at G2/M phase and apoptosis based on Western blot and flow cytometry assays.

## 1. Introduction

The World Health Organization classifies brain tumors into four grades (I–IV) based on histopathological observations and their severity [1]. Glioblastoma multiforme (GBM) is one of the most lethal and highly aggressive grade IV brain and spinal cord tumors that usually occur in glial cells of the central nervous system [2]. Statistically, GBM accounts for approximately 45.2% of primary malignant brain tumors in adult patients [3]. Chemoradiotherapy for GBM has many treatment challenges, such as the rapid proliferation rate of GBM cells, the differentiation of treatment-resistant cell clones, and the difficulty of passing through the blood–brain barrier to access the brain parenchyma, etc. [4]. Because of the infiltrative, diffuse, and sporadic nature of GBM, traditional surgical resection and concomitant chemoradiotherapy only extend the median survival rate to 14 months and improve the mean 3-year survival from 1.9% to 16% [5].

Oral imidazotetrazine alkylating agent temozolomide (TMZ) is the most widely used first-line chemotherapeutic drug for GBM patients [6]. After entering cells, TMZ is converted to 3-methyl-(triazen-1-yl)imidazole-4-carboxamide and induces methylation of the O6-guanine, N7-guanine, and N3-adenine in DNA, inducing cell cycle arrest in G2/M phase, thereby inhibiting cancer cell proliferation [7]. However, the hematological toxicity and strong resistance of TMZ constrain its efficacy in clinical use [8]. The high resistance against methylguanine-DNA-methyltransferase, which often occurs during chemotherapy, results in a poor response to TMZ treatment in approximately 50% of patients. More importantly, gliomas obtain TMZ resistance through multiple mechanisms during TMZ treatment, and many patients with high methylguanine-DNA-methyltransferase expression are naturally resistant to TMZ [9]. Other first-line anti-glioma drugs, such as carmustine [10], lomustine [11], and the humanized monoclonal antibody bevacizumab [12], result in pulmonary toxicity, hypertension, and leucopenia [13]. Therefore, the development of new chemotherapeutic drugs for GBM treatment is still urgent.

As a potent tumor suppressor, the p53 pathway is one of the most promising targets in tumors [14]. Restoring p53 function to induce apoptosis or growth arrest has been considered a practical approach to restrain cancer. However, more than half of GBM patients exhibit p53 positivity [15]. p53 is prominently regulated by mouse double-minute protein (MDM) 2 and its homolog MDM4 [16], which bind to the N-terminal transactivation domain of p53, blocking its transcriptional function. Downregulation of p53 protein is responsible for the proliferation, invasion, migration, avoidance of apoptosis, and other properties of GBM cells [17]. Therefore, reducing MDM4 or MDM2 expression and restoring p53 function represent an attractive GBM treatment strategy. Various MDM intracellular protein–protein interaction inhibitors have been developed to stabilize p53 for cancer treatment [18,19]. Although several MDM2 inhibitors have entered clinical trials, increasing evidence suggests that enhanced inhibition of MDM4 remains critical for this class of inhibitors to exert more sensitive and potent activity to release p53 [20]. Because the binding domain of MDM4 to p53 contains a peptide sequence and three-dimensional structure that is highly similar to MDM2, the development of selective MDM4 inhibitor remains challenging [21].

**Figure 1:**
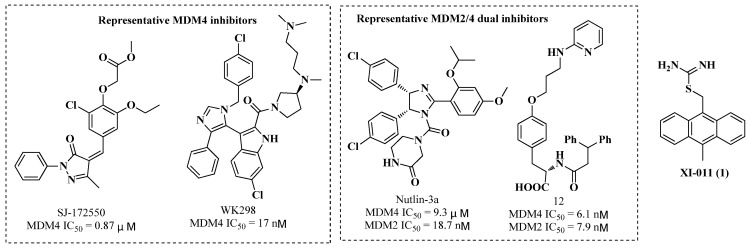
Structures of representative MDM4 inhibitors and **XI-011**.

In previous studies, we discovered a small molecule, (10-methylanthracen-9-yl)methyl carbamimidothioate (**XI-011**) (Figure 1), which effectively inhibits MDM4 expression in cancer cells [22,23]. Recent studies have demonstrated that **XI-011** effectively binds to the oncogenic driver heterogeneous nuclear ribonucleoprotein A2B1 and disrupts the heterogeneous nuclear ribonucleoprotein A2B1/MDM4 promoter and untranslated region interaction, thereby activating and stabilizing p53, and inhibiting cancer cell growth [24]. Here, 43 new analogs of **XI-011** were designed, synthesized, and evaluated for their anti-glioma activities.

## 2. Results and Discussion

### 2.1. Chemistry

**Scheme 1:**
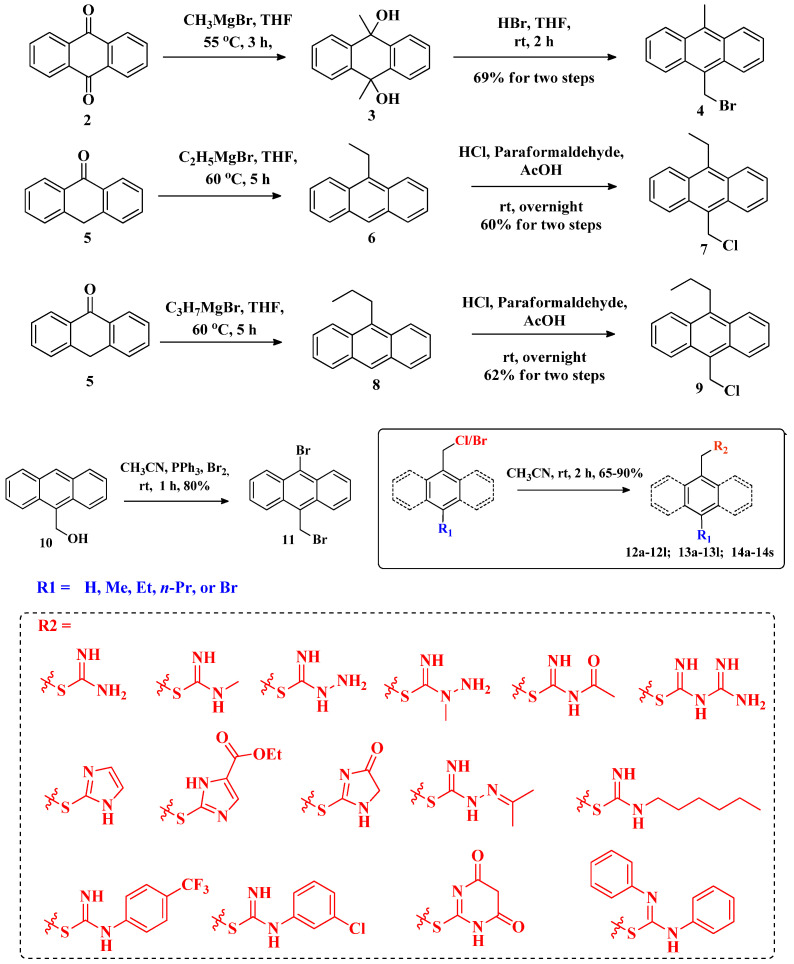
General synthetic procedure of compounds **12a**–**12l**, **13a**–**13l**, and **14a**–**14s**.

The general synthetic routes of targeted **12a**–**12l**, **13a**–**13l**, and **14a**–**14s** are outlined in Scheme 1. Synthesis of targeted **12a**–**12l**, **13a**–**13l**, and **14a**–**14s** is first required for the synthesis of halogenated derivatives (**4**, **7**, **9**, and **11**). 9-(Bromomethyl)-10-methylanthracene **4** was prepared from anthracene-9,10-dione **2** by treatment with CH_3_MgBr at 55 °C, followed by aromatization and bromination in the presence of a 40% HBr solution to generate **4** with a 69% yield. Synthesis of ethyl **7** and propyl **9** analogs started from anthracene-9(10*H*)-one **5**, which were, respectively, reacted with CH_3_CH_2_MgBr and CH_3_CH_2_CH_2_MgBr at 60 °C to produce 9-substituted anthracenes (**6** and **8**). Next, targets **7** and **9** were prepared from synthesized 9-substituted anthracenes (**6** and **8**) via the Mannich reaction with paraformaldehyde/HCl, resulting in 60–62% yields.

The bromination reaction of anthracen-9-ylmethanol **10** proceeded smoothly in the presence of PPh_3_/Br_2_ at room temperature to yield 9-bromo-10-(bromomethyl)anthracene **11** with an 80% yield. Using **4**, **7**, **9**, and **11**, various thiourea derivatives finally substituted the chloro or bromo group of anthracene to yield the corresponding compounds **12a**–**12l**, **13a**–**13l**, and **14a**–**14s**.

### 2.2. In Vitro Antiproliferative Activity Analysis

To determine whether these synthesized **XI-011** analogs had desired p53 activation and antiproliferative activities, all compounds were tested for their antiproliferative effect at 10 µM in the human glioblastoma cell line U87.

As shown in Table 1, **XI-011** and doxorubicin (DOX) were chosen as controls. **XI-011** and DOX induced high inhibition rates of U87 cells at 10 µM (91.8% and 81.1%, respectively). DMSO (1%) was used as the vehicle control. SAR analysis of most small sterically hindered analogs, such as 1-naphthyl, 2-naphthyl, and benzyl analogs (**12a**–**12f**), showed slight inhibition of U87 cells, and the inhibition rates were maintained at 1.5–15.2%. Whether the methyl group was substituted on the naphthalene ring (**12a** vs. **12b**) had little effect on the inhibitory activity. Hydrazinecarbimidothioate analogs **12f** and **12g** exhibited poor inhibition rates of 13.1% and 14.5% at 10 µM, respectively. The quinolinyl-derived hydrazinecarbimidothioate analog (**12k**) only had a 10.7% inhibition rate in U87 cells with similar potency to **12g**. Notably, after replacing the naphthyl group with a 10-methylanthracenyl group, the resulting compound **13a** exhibited approximately 4-fold more potency than **12f**, suggesting that the 10-methylanthracenyl was optimal for cell inhibition.

Next, we explored more substitutions with various thiourea groups for potency while keeping the 10-methylanthracenyl. As indicated in Table 2, moderate U87 inhibitory profiles were observed when the synthesized compounds featured an acetyl (**13b**) or amidinyl (**13c**) group. Aryl groups, such as phenyl (**13j** and **13k**), imidazolyl (**13g** and **13i**), and pyrimidine-4,6(1*H*,5*H*)-dione-derived (**13h**) analogs, all showed a significant decrease in the inhibition rate in U87 cells. Compared with these aryl-derived compounds, long-chain alkyl (**13l**) and imine (**13f**) analogs exhibited good anti-glioma activity and had a 3–4-fold potency improvement in U87 cells. Remarkably, the compound **13d** and methylhydrazine-derived analog **13e** showed the highest anti-glioma potency, with 93.3% and 88.6% inhibition rates at 10 µM.

Encouraged by the promising anti-glioma effect of analogs **13d** and **13e**, we performed further optimization of the anthracenyl skeleton.

After replacing the methyl-substituent with an ethyl group at the 10-position of anthracene, seven new analogs (**14a**–**14g**) were prepared and evaluated for their anti-glioma activity. As shown in Table 3, analogs **14a**–**14c** exhibited good potency, indicating that introducing a longer hydrophobic substituent on the 10-position might be beneficial for improving their anti-glioma activity. However, the compounds with acetyl (**14e**), amidinyl (**14d**), hexyl (**14f**), and *p*-CF_3_-phenyl (**14g**) groups showed 3~4-fold decreases in anti-glioma activity. Further increasing the chain length by one methylene unit (**14h**) at the 10-position of anthracene led to a slight decrease in potency relative to **14a**. After removing the alkyl-substituents, compounds **14i**–**14l** exhibited weak to moderate anti-glioma activity. Only analog **14i** displayed pronounced good anti-glioma activity (83.0%) at 10 μM. A similar trend was observed when the methyl-substituent was replaced by a bromo-substituent group (**14i** vs. **14n** and **14o** vs. **14j**). Three acridine analogs (**14p**–**14r**) had weak inhibition rates against U87 cells, demonstrating that the heteroatoms in the anthracenyl skeleton were not well tolerated. Notably, compound **14s** with a non-rigid structure exhibited approximately 12-fold less potency than **XI-011**, indicating that the rigid anthracenyl skeleton was necessary.

Considering the potent inhibitory activities at 10 µM, the IC_50_ values of **13d**, **13e**, **14a**, **14b**, and **14n** were further investigated in human glioblastoma cell line U87, human cervical cancer cell line HeLa, human breast cancer cell line MCF-7, and HHL-5 as a human normal cell line by MTT assays. As shown in Table 4, all compounds showed good in vitro antitumor activities against the three cancer cell lines. Compound **13e** showed effective anti-cervical activity against HeLa cells (IC_50_ = 1.63 µM) and MCF-7 cells (IC_50_ = 0.97 µM), which was similar to that of Dox (Hela: IC_50_ = 0.54 µM; MCF-7: IC_50_ = 1.32 µM). The methyl and ethyl groups in anthracene (**14a**, **14b**, and **XI-011**) might be crucial for the anti-cervical cancer activity, and substitution with bromo (**14n**) resulted in a 1–2-fold reduction in activity. Notably, **13e** exhibited the best anti-breast cancer cell activity in MCF-7 cells, which was 5–8-fold more potent than that of **13d**, **14a**, **14b**, and **XI-011**.

As anticipated, compound **13e** also exhibited the best anti-glioma activity (IC_50_ = 0.53 µM), which was similar to those of the controls **XI-011** (IC_50_ = 0.69 µM) and Dox (IC_50_ = 0.44 µM). Notably, **13e** exhibited more potential cytotoxicity to U87 cells (IC_50_ = 0.53 µM) and 2-fold lesser toxicity to human normal HHL-5 cells (IC_50_ = 1.11 µM). Other analogs (**13d**, **14a**, **14b**, and **14n)** also had cell growth inhibitory IC_50_ values of less than 2 µM in U87 cells, demonstrating that these anthracenyl skeleton compounds were efficient and promising for developing new anti-glioma drugs.

### 2.3. Mechanistic Analyses of ***13e*** against U87 Cells

**Figure 2:**
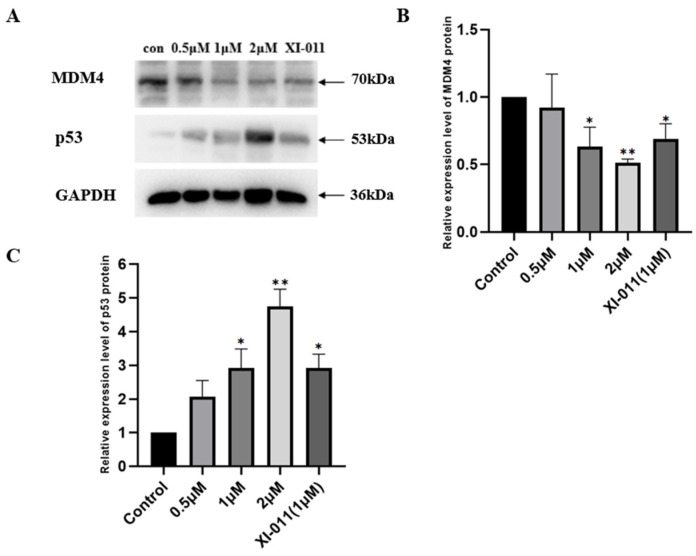
(**A**) Western blot analysis of p53 and MDM4 expression in U87 cells after 24 h of treatment with **XI-011** (1.0 µM) and **13e** (0.5, 1, and 2 µM). Glyceraldehyde 3-phosphate dehydrogenase (GAPDH) was used as the loading control. (**B**) Effect of **13e** on MDM4 expression in U87 cells (*n* = 3). (**C**) Effect of **13e** on p53 expression in U87 cells (*n* = 3). Data are represented as the mean ± SD (error bars). * *p* < 0.05, ** *p* < 0.01.

Considering the good antiproliferative effect of **13e**, the expression of MDM4, p53, and GAPDH was evaluated by Western blot analysis after treating U87 cells with **13e**. As shown in Figure 2, after 24 h of treatment with **13e,** we observed dose-dependent suppression of MDM4 expression and remarkable stabilization of p53 protein in U87 cells, which was consistent with its cytotoxicity. Notably, compound 13e significantly upregulated p53 protein expression, demonstrating similar effects at 1 µM compared with **XI-011**. Collectively, these findings provide compelling evidence that compound 13e may exert a strong suppressive effect on MDM4 expression, stabilize p53, and upregulate p53 expression through an MDM4–p53-dependent mechanism, which is similar to the effects of **XI-011**.

**Figure 3:**
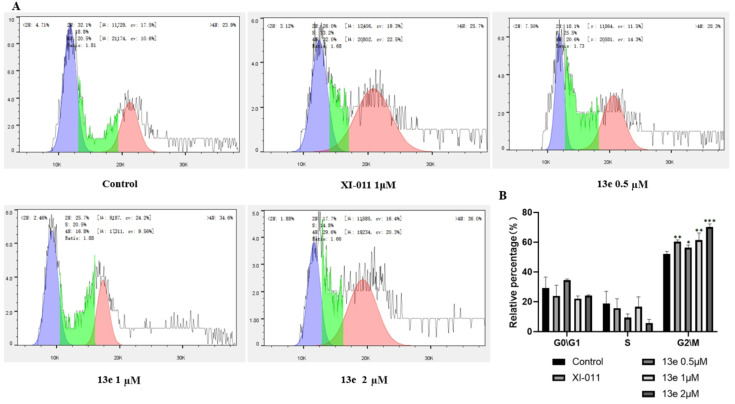
(**A**) Flow cytometric analysis of the cell cycle distribution of U87 cells in the presence of **XI-011** (1.0 µM) and **13e** (0.5, 1, and 2 µM) for 24 h. (**B**) Cell cycle distribution of U87 cells treated with **13e** and **XI-011**. The experiments were repeated three times. * *p* < 0.05, ** *p* < 0.01, and *** *p* < 0.001 compared with the control group.

Next, we conducted flow cytometric analysis of cell cycle progression in U87 cells treated with **13e** to investigate its cellular mechanisms. As shown in Figure 3, **13e** at three concentrations arrested U87 cells in G2/M phase and demonstrated a dose-dependent effect similar to **XI-011**. Compared with the control, treatment with 0.5, 1, and 2 µM **13e** decreased the proportion of U87 cells in S phase and increased the proportion of U87 cells in the G2/M fraction. These results indicated that the strong antiproliferative activity of **13e** in U87 cells was caused by U87 cell cycle arrest at G2/M phase.

**Figure 4:**
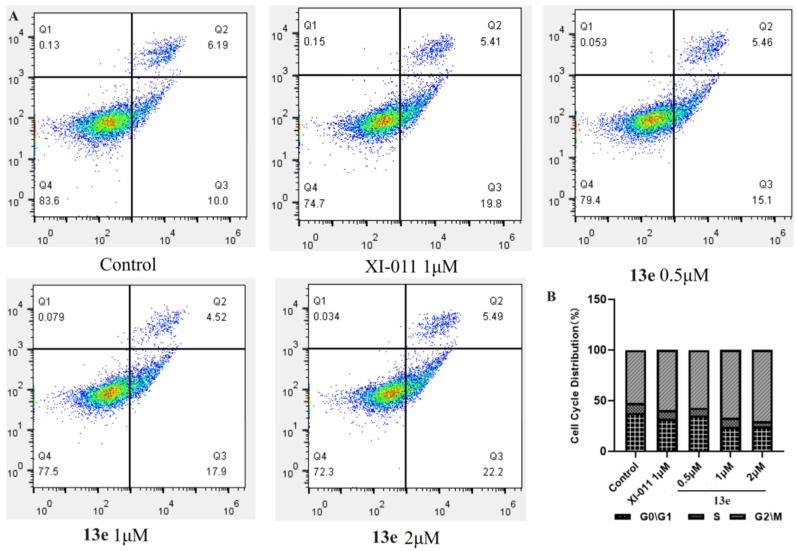
(**A**) Flow cytometric analysis of apoptosis induction in U87 cells in the presence of **XI-011** (1.0 µM) and **13e** (0.5, 1, and 2 µM) for 24 h. (**B**) Histograms of apoptotic cells (%). Data are shown as the mean ± SD of three independent experiments.

Reducing MDM4 expression restores the p53 function and increases apoptotic cells [25]. The apoptotic effects of compound **13e** were determined in U87 cells by flow cytometry. As shown in Figure 4, compounds **13e** and **XI-011** dose-dependently induced U87 cell apoptosis. **13e** induced 21% apoptosis at 1 µM, which was slightly lower than the 23% apoptosis induced by **XI-011**. At 2 µM **13e**, apoptosis was up to 25.9%. These results provide evidence that compound **13e** effectively induces apoptosis in U87 cells. The mechanism by which **13e** induces apoptosis in U87 cells may be related to its ability to downregulate MDM4 expression and stabilize p53 protein.

### 2.4. Molecular Docking of ***13e***

**Figure 5:**
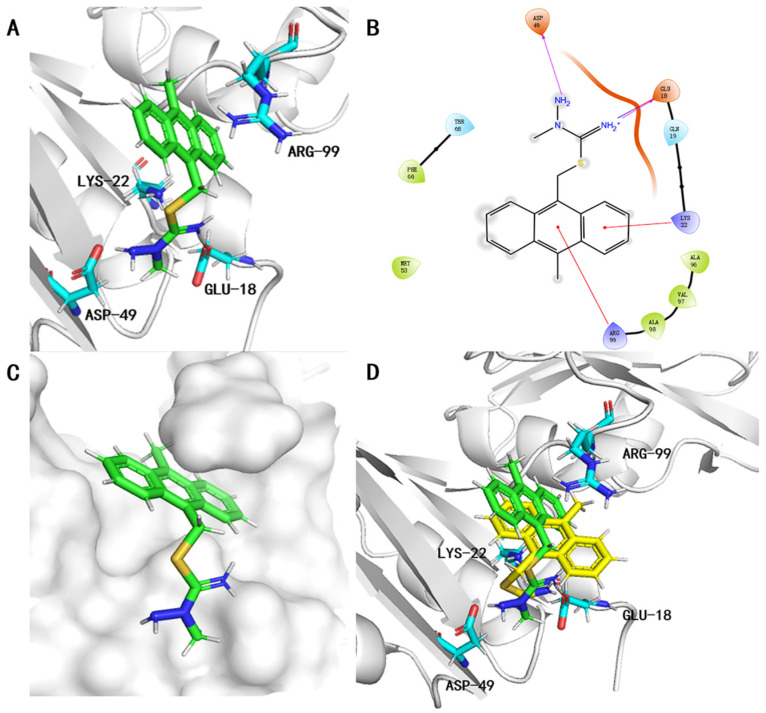
Overview of **13e** and **XI-011** binding to the hnRNPA2B1 protein pocket (PDB code: 5HO4). (**A**) Three-dimensional view of **13e** (green) with hnRNPA2B1 protein (gray). The carbon, sulfur, and nitrogen atoms of **13e** are displayed in green, brown, and blue, respectively. The carbon, oxygen, and nitrogen atoms of amino acid residues in hnRNPA2B1 are displayed in light blue, red, and blue, respectively. (**B**) Two-dimensional view of **13e** with hnRNPA2B1 protein. (**C**) hnRNPA2B1 protein shown by gray surface representation and **13e** (green) shown as sticks. (**D**) Overlap of **13e** (green) and **XI-011** (yellow).

In our previous studies, **XI-011** disrupted recruitment of the heterogeneous nuclear ribonucleoprotein A2B1 (hnRNPA2B1) to the promoter and untranslated region of MDM4, leading to inhibition of MDM4 transcription. Therefore, we analyzed the binding mode of the **XI-011** analog **13e** and hnRNPA2B1 protein (PDB code: 5HO4) (Figure 5). The results revealed that the anthracenyl skeleton of compound **13e** was well accommodated and positioned similarly to that of **XI-011** in the cavity of hnRNPA2B1. The -NH_2_ and imine groups in **13e** formed crucial hydrogen bond interactions with the carboxyl group in Asp-49 and the carboxyl group in Glu-18. Additionally, the anthracenyl group exhibited cation-π interactions with the terminal amino group of Lys-22 and the guanidine group in Arg-99. As anticipated, we observed similar binding modes of **13e** and **XI-011** to hnRNPA2B1 (Figure 5D). These interactions may explain the effective inhibition of MDM4 expression by **13e**.

Accumulated evidence has shown that MDM4 inhibition plays a crucial role in reinstating p53 functions. In this study, structural modifications and SAR analysis were conducted based on the reported p53 activator **XI-011**. Convenient and efficient synthesis is imperative for the effectiveness of new drug development. Here, anthracenyl skeleton compounds were prepared efficiently by two to three linear steps with good yields. SAR analysis indicated that the anthracenyl skeleton analogs exhibited significant potency against glioma cells compared with phenyl and naphthyl analogs. Interestingly, compounds with a small steric hindrance thiourea substitution exhibited more potent antiproliferative activities compared with their counterparts with bulky steric hindrance. Five anthracenyl skeleton compounds were selected and evaluated for their antiproliferative activities in three cancer cell lines, namely U87, Hela, and MCF-7. Remarkably, compound **13e** exhibited antiproliferative effects similar to those of doxorubicin and demonstrated slight selectivity of human cancer U87 cells compared with normal HHL-5 cells. These results suggest that 13e is a promising candidate for cancer therapy.

**Figure 6:**
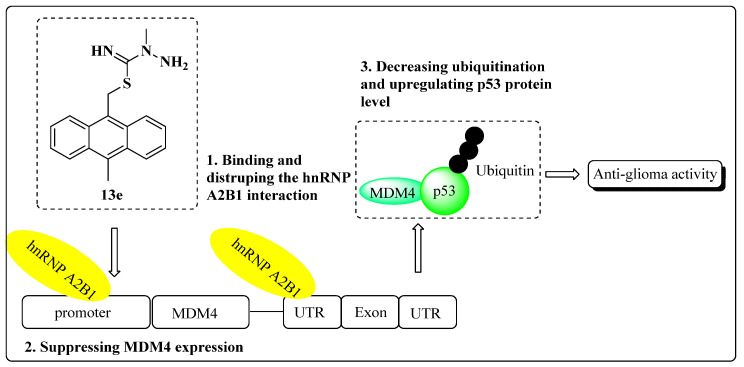
Proposed anti-glioma mechanism of **13e**.

To verify whether the antiproliferative mechanism of **13e** was similar to that of **XI-011**, we conducted a series of experiments, including Western blotting, cell cycle assays, and molecular docking. The results provided evidence that analogs of the anthracenyl skeleton compounds inhibited MDM4 expression, stabilized p53 protein, and induced apoptosis in U87 glioma cells. On the basis of these findings and our previous studies, a proposed anti-glioma mechanism of **13e** is illustrated in Figure 6. **13e** might bind to the interaction between hnRNPA2B1 protein and the MDM4 promoter and untranslated region, which was verified by molecular docking analyses. Next, p53 protein expression is upregulated, ultimately resulting in apoptosis of U87 cells.

Several limitations of this study should be highlighted. This study relied on in vitro experiments, restricting insights into the drugs’ ability to permeate the blood–brain barrier, which is crucial for GBM treatment. Future studies should validate these findings through experiments involving the blood–brain barrier and xenograft models. Additional experimental evidence is also necessary to fully elucidate the antitumor mechanism of these anthracene skeleton compounds. Moreover, further exploration of combining anthracenyl skeleton compounds, which demonstrate the potential for p53 activation, with other antitumor drugs is warranted.

## 3. Materials and Methods

### 3.1. Cytotoxicity Evaluation

U87, Hela, MCF-7, and HHL-5 cells were provided by Chinese Academy of Medical Sciences (Beijing, China). The cancer cells were cultured in DMEM media containing 10% heat-inactivated fetal calf serum. All cells were incubated at 37 °C in a 5% CO_2_ humidified atmosphere and harvested in the exponential growth phase for assays.

### 3.2. MTT Assay

Cells were seeded in a 96-well plate and treated with the test substance at the desired concentration for 72 h. In cytotoxicity experiments, cells were treated with 10 µM of the test compound or 1 µM **XI-011** and doxorubicin as positive controls. DMSO (1%) was used as a negative control. In the IC_50_ analysis, doxorubicin, **XI-011**, **13d**, **13e**, **14a**, **14b**, and, **14n** were applied to HHL-5 at 50, 25, 10, 5, 2.5, 1.25, 0.625, 0.3125, and 0.15625 µM and U87 cells at 10, 7.5, 5, 25, 1.25, 0.625, 0.3125, and 0.15625 µM, whereas 30, 10, 3, 0.5, and 0.1 µM were applied to Hela and MCF-7 cells. Treated cells were incubated with an MTT solution for 2 h and then with DMSO to dissolve the crystals. Absorbance was determined at 570 nm using a microplate reader (BioTek, Seattle, DC, USA). IC_50_ values were calculated using the Hill model in GraphPad Prism 8.0.2 software.

### 3.3. Cell Cycle Analysis

Cells were cultured for 24 h in a 6-well plate, and then the test compound (0.5, 1, and 2 µM) was added to the culture medium. After treatment for 24 h, the cells were harvested and fixed at 4 °C overnight in an ethanol solution (80%). The cells were washed with PBS and stained with a PI solution (20 mg/mL PI and 20 mg/mL RNase in PBS). After 30 min, the cells were subjected to flow cytometry (C6; BD, Franklin Lakes, NJ, USA). The recorded cell fluorescence was used to analyze the cell cycle distribution.

### 3.4. Western Blotting

Protein levels in U87 cells treated with test compound **13e** (0.5, 1, and 2 µM) for 24 h were measured by Western blot analysis as described previously [26]. Briefly, after **13e** treatment, cells were lysed in RIPA buffer. Proteins were separated by 8% SDS-PAGE and transferred to a PVDF membrane (Millipore, Billerica, MA, USA). The membrane was incubated with an anti-MDM4 antibody (A300-287A, Bethyl Laboratories, Montgomery, TX, USA), anti-p53 antibody (sc-126, Santa Cruz Biotechnology, Inc., Dallas, TX, USA), or anti-GAPDH antibody (AG019, Beyotime, Shanghai, China) followed by an HRP-linked secondary antibody (A0216, Beyotime) for 2 h. ECL reagent was used for chemiluminescent development. Densitometric analysis of protein bands was performed using ImageJ V.1.8.0 software.

### 3.5. Data and Statistical Analysis

Data are presented as the mean ± SD. Statistical analysis was performed using GraphPad Prism 8.0.2 Software. Differences among groups were assessed by the *t*-test. *p* < 0.05 was considered statistically significant.

### 3.6. Chemistry Section

#### 3.6.1. General Methods

All reagents were purchased from commercial suppliers. Reactions under standard conditions were monitored by thin-layer chromatography on F254 gel plates. Flash chromatography was performed using a silica gel (200–300 mesh). High-resolution mass spectrometric data were acquired using a Q-TOF analyzer (Thermo Fisher Scientific, BRE, German). ^1^H and ^13^C NMR spectra were recorded using 500 MHz/125 MHz Bruker spectrometers (Billerica, MA, USA) or 400 MHz/100 MHz JEOL JNM-ECZ400S spectrometers (Akishima, Japan). The coupling constant (*J*) was Hz. Signals were reported as follows: s (singlet), d (doublet), t (triplet), q (quartet), dd (doublet of doublets), dt (doublet of triplets), ddt (doublet of doublets of triplets), dq (doublet of quartets), br s (broad singlet), and m (multiplet). The purity of **13e** was high (≥99.2%). ^1^H and ^13^C nuclear magnetic resonance (NMR) spectroscopy data of compounds **12a**–**12l, 13a**–**13l**, and **14a**–**14s**, and HPLC spectra of **13e** are shown in Supplementary Materials.

#### 3.6.2. Synthesis of Compound **4**

In an argon atmosphere, **2** (50 mmol) was dissolved in 350 mL of dry tetrahydrofuran. A solution of methylmagnesium bromide (100 mL, 1.5 M/L, 150 mmol) was slowly added dropwise to the solution at 50 °C. The reaction mixture was heated and stirred at 50 °C for 3 h. Subsequently, the reaction was quenched with 100 mL water and extracted with dichloromethane (3 × 80 mL). The organic phase was combined, dried with anhydrous sodium sulfate, filtered, and evaporated in a vacuum to yield crude 9,10-dimethyl-9,10-dihydroanthracene-9,10-diol **3** without further purification.

Next, **3** (10 mmol) was dissolved in 250 mL tetrahydrofuran, and 50 mL of 48% hydrobromic acid was added to the reaction mixture, followed by stirring for 2 h at room temperature. The yellow precipitate was filtered and collected to recover compound **4** (69% yield).

#### 3.6.3. Synthesis of Compound **7**

Compound **5** (50 mmol) was dissolved in 50 mL anhydrous toluene. Subsequently, ethylmagnesium bromide (100 mL, 1.5 M, 150 mmol) was added dropwise to the solution at rt. The reaction mixture was heated to 50 °C and stirred for 5 h. Then, 50 mL of 6N HCl was added to quench the reaction. After removing the toluene in a vacuum, CH_2_Cl_2_ (150 mL) was added to dissolve the residue. The residue was then extracted with a 10% NaOH solution five times (5 × 20 mL each). The combined organic phase was dried over sodium sulfate and concentrated in a vacuum to yield the crude product of 9-ethylanthracene **6** without further purification.

Paraformaldehyde (62.5 mmol) was dissolved in dried glacial acetic acid (70 mL), followed by slow addition of dry HCl gas to the reaction until the paraformaldehyde was completely dissolved. 9-Ethylanthracene **6** (25 mmol) was added to the reaction mixture, followed by stirring at 25 °C overnight. H_2_O (150 mL) was added to quench the reaction, followed by filtering out the yellow solid **7** without further purification, yielding 60%.

#### 3.6.4. Synthesis of Compound **9**

Compound **9** was synthesized following a similar synthetic procedure to that for **7** using compound **5** and propyl magnesium bromide to produce 9-propylanthracene. This was followed by generation of 9-(chloromethyl)-10-propylanthracene **9** with a yield of 62%.

#### 3.6.5. Synthesis of Compound **11**

PPh_3_ (5 mmol) was dissolved in anhydrous acetonitrile (15 mL). The solution was purged with nitrogen for 20 min, followed by gradual addition of 0.5 mL bromine (10 mmol) to the solution using an airtight syringe. Commercially available **10** (2.5 mmol) was added to the reaction, followed by stirring at room temperature for 1 h. The precipitate was filtered, collected at 0 °C, and recrystallized from chloroform to recover 9-bromo-10-(bromomethyl)anthracene **11** with an 80% yield.

#### 3.6.6. General Synthesis Procedure for **XI-011**, **12a**–**12l**, **13a**–**13l**, and **14a**–**14s**

A halogenated compound (**4**, **7**, **9**, or **11**) (5 mmol) was dissolved in an acetonitrile solution and then treated with various commercially available thiourea compounds (7.5 mmol). After stirring for 2 h at room temperature, the precipitate was filtered out and sequentially washed with 50 mL petroleum ether, 50 mL dichloromethane, and 50 mL ethyl acetate. After drying, the desired products were obtained with various yields.

### 3.7. Computational Modeling Methods

The X-ray crystal structure of hnRNPA2B1 (PDB:5HO4) was obtained from the Protein Data Bank and used as the receptor for docking analysis. Protein geometry was generated with a Dreiding-like force field by the Discovery Studio 2018 toolbox standard procedure. The protein complex was prepared by monitoring bad valency, removing water molecules, and adding hydrogen. The CHARMm force field was then applied to the receptor with **13e** and **XI-011**. Active site spheres (10 Å diameter) centered on the cognate ligand were automatically generated. The remaining parameters were the default settings.

## 4. Conclusions

Forty-three analogs of p53 activator **XI-011** were designed and synthesized as potent inhibitors of U87 cells. Among them, five new compounds (**13d**, **13e**, **14a**, **14b**, and **14n**) exhibited good anti-glioma activity at the cellular level. Remarkably, compound **13e** exhibited excellent in vitro anti-glioma activity, with an IC_50_ value of 0.53 µM. Additionally, **13e** showed effective anti-cancer activities against HeLa cells (IC_50_ = 1.63 µM) and MCF-7 cells (IC_50_ = 0.97 µM). Antitumor mechanistic analyses indicated that **13e** efficiently suppressed the expression of MDM4 and promoted p53 activation to induce U87 cell cycle arrest at G2/M phase, inducing cancer cell apoptosis. Taken together, our study showcases a series of new p53 activators featuring an anthracenyl skeleton that are promising for future development for GBM treatment.

## Figures and Tables

**Table 1:** In vitro anti-proliferative activities of **12a**–**12l**, and **13a** ^a^.

Cmpd	Structure	U87 (% Inhib. @ 10 µM)	Alog*P*	Cmpd	Structure	U87 (% Inhib. @ 10 µM)	Alog*P*
**XI-011**	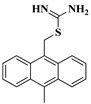	91.8	4.65	**12h**	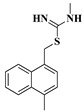	9.9	4.17
**12a**	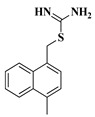	15.2	3.65	**12i**	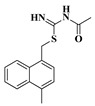	2.7	3.58
**12b**	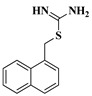	9.0	3.16	**12j**	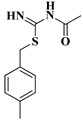	6.8	2.58
**12c**	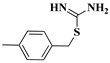	11.8	2.65	**12k**	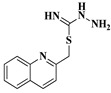	10.7	2.65
**12d**	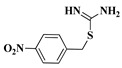	14.8	1.44	**12l**	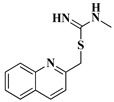	11.3	3.19
**12e**	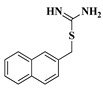	1.5	3.16	**13a**	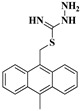	57.9	4.62
**12f**	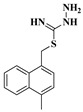	13.1	3.62	DOX		81.1	
**12g**	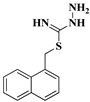	14.5	3.14	DMSO		4.7	

^a^ Inhibition rate (%) of U87 cells treated with 10 µM of a compound for 72 h, as determined by MTT assays. Each experiment was carried out using triplicates. *p* < 0.05 compared with the control group. Alog*P* is the Ghose–Crippen octanol–water partition coefficient of a compound. Doxorubicin (DOX; 1 µM) was used as the positive control. DMSO (1%) was used as the vehicle control.

**Table 2:** In vitro antiproliferative activities of **13a**–**13l** ^a^.

Cmpd	Structure	U87 (% Inhib. @ 10 µM)	Alog*P*	Cmpd	Structure	U87 (% Inhib. @ 10 µM)	Alog*P*
**13a**	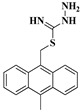	57.9	4.62	**13h**	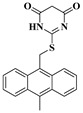	0.7	4.56
**13b**	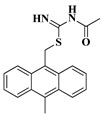	19.3	4.58	**13i**	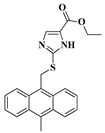	31.2	5.18
**13c**	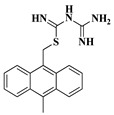	35.4	4.93	**13j**	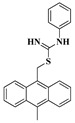	13.3	6.83
**13d**	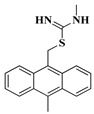	93.3	5.17	**13k**	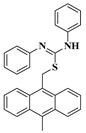	28.1	8.73
**13e**	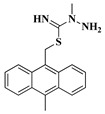	88.6	4.92	**13l**	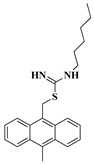	86.7	7.24
**13f**	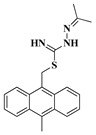	65.1	5.47	DOX		81.1	
**13g**	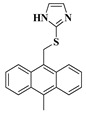	41.2	4.97	DMSO		4.7	

^a^ Inhibition rate (%) of U87 cells treated with 10 µM of a compound for 72 h, as determined by MTT assays. *p* < 0.05 compared with the control group. Alog*P* is the Ghose–Crippen octanol–water partition coefficient of a compound. Doxorubicin (DOX; 1 µM) was used as the positive control. DMSO (1%) was used as the vehicle control. Each experiment was carried out using triplicates.

**Table 3:** In vitro anti-proliferative activities of **XI-011** and **14a**–**14s** ^a^.

Cmpd	Structure	U87 (% Inhib. @ 10 μM)	Alog*P*	Cmpd	Structure	U87 (% Inhib. @ 10 μM)	Alog*P*
**XI-011**	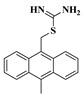	91.8	4.65	**14k**	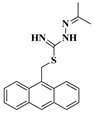	14.2	4.99
**14a**	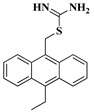	79.0	5.06	**14l**	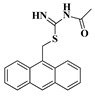	0.7	4.09
**14b**	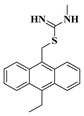	94.7	5.58	**14m**	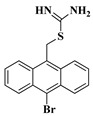	36.8	4.99
**14c**	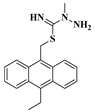	83.6	4.72	**14n**	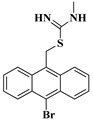	86.5	5.51
**14d**	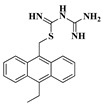	17.3	5.35	**14o**	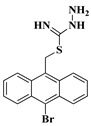	50.7	4.96
**14e**	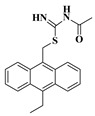	19.3	5.00	**14p**	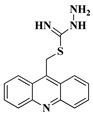	19.0	3.64
**14f**	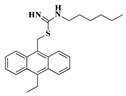	43.6	7.66	**14q**	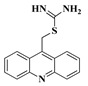	12.5	3.67
**14g**	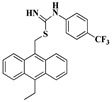	28.0	8.17	**14r**	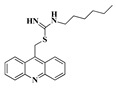	2.1	6.27
**14h**	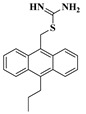	64.6	5.48	**14s**	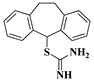	6.9	4.35
**14i**	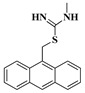	76.4	4.68	DOX		81.1	
**14j**	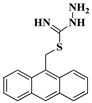	11.8	4.13	DMSO		4.7	

^a^ Inhibition rate (%) of U87 cells treated with 10 µM of a compound for 72 h, as determined by MTT assays. *p* < 0.05 compared with the control group. Alog*P* is the Ghose–Crippen octanol–water partition coefficient of a compound. Doxorubicin (DOX; 1 µM) was used as the positive control. DMSO (1%) was used as the vehicle control. Each experiment was carried out using triplicates.

**Table 4:** In vitro antiproliferative activities of **XI-011**, **13d, 13e**, **14a**, **14b**, and **14n** in cancer cell lines U87, Hela, and MCF-7, and HHL-5 as a normal cell line ^a^.

Cmpd	IC_50_ (μM)			
	U87	Hela	MCF-7	HHL-5
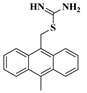 (**XI-011**)	0.69 ± 0.02	1.51 ± 0.06	4.87 ± 0.30	0.55 ± 0.03
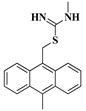 (**13d**)	1.62 ± 0.02	5.66 ± 0.29	4.74 ± 0.20	0.85 ± 0.10
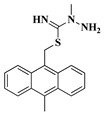 (**13e**)	0.53 ± 0.03	1.63 ± 0.25	0.97 ± 0.11	1.11 ± 0.08
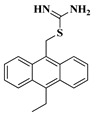 (**14a**)	1.24 ± 0.24	3.64 ± 0.27	4.56 ± 0.03	1.51 ± 0.16
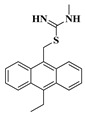 (**14b**)	1.35 ± 0.10	2.66 ± 0.19	4.55 ± 0.27	1.62 ± 0.13
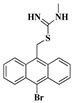 (**14n**)	1.84 ± 0.12	7.74 ± 0.81	7.60 ± 1.37	1.67 ± 0.16
Dox	0.44 ± 0.06	0.54± 0.05	1.32 ± 0.16	0.32 ± 0.02

^a^ IC_50_ values were calculated based on at least three independent experiments using MTT assays. Doxorubicin (Dox) was used as the positive control. Cells were treated with **XI-011**, Dox, **13d**, **13e**, **14a**, **14b**, and **14n** for 72 h. Data are represented as the mean ± SD (n = 3).

## Data Availability

Data are contained within the article and Supplementary Materials.

## Supplementary Materials

The following supporting information can be downloaded at: https://www.mdpi.com/article/10.3390/molecules29102396/s1. The characterization of synthesized compounds (**XI-011**, compounds **12a**–**12l**, compounds **13a**–**13l**, compounds **14a**–**14s**). Figures S1–S88: NMR spectra for 44 products (**XI-011**, compounds **12a**–**12l**, compounds **13a**–**13l**, compounds **14a**–**14s**). The HPLC spectra of **13e** (Figure S89). Dose–response curves of compounds (XI-011, **13d**, **13e**, **14a**, **14b**, **14n**, and DOX) on the cytotoxicity of U87-MG, Hela, MCF-7, and HHL-5 cells (Figures S90–S96). The origin western blots results of **13e** and **XI-011** in U87 cells ( Figures S97–S99).
