# Supplementary material for: Novel 9-Methylanthracene Derivatives as p53 Activators for the Treatment of Glioblastoma Multiforme
Source: Molecules. 2024 May 19;29(10):2396. doi: 10.3390/molecules29102396 (PMC11123991; doi:10.3390/molecules29102396)

Supporting Information

Novel 9-Methylanthracene Derivatives as p53 Activators for the Treatment of Glioblastoma multiforme

Yuxin Feng[1], Yingjie Wang[1], Xiaoxue Li[2], Ziqiang Sun[2], Sihan Qiang[2], Hongbo Wang[1]*, Yi Liu[2]*

[1] Key Laboratory of Molecular Pharmacology and Drug Evaluation (Yantai University), Ministry of Education, Yantai University, Yantai 264005, China

[2] School of Chemistry and Chemical Engineering, Yantai University, Yantai 264005, China;

* Correspondence: liuyi@ytu.edu.cn(Y.L.); hongbowangyt@163.com (H.W)

Contents

1. Characterization of compound (XI-011, 12a-12l, 13a-13l, and 14a-14s) S3

2. NMR spectra of synthesized compounds S11

3. HPLC spectra of 13e S57

4. Dose–response curves of compounds on the cytotoxicity of U87-MG, Hela, MCF-7, and HHL-5 cells S58

5. Origin western blots results in U87 cells treatment with 13e S65

5. Characterization of compound (XI-011, 12a-12l, 13a-13l, and 14a-14s)

XI-011, 12a-12l, 13a-13l, and 14a-14s were prepared following the general synthesis procedure as described in the main text.

XI-011, yellow solid with 86 % yield.^{1}H NMR (400 MHz, DMSO-d_6) δ: 9.43 (s, 3H), 8.45 (dd, J = 8.0 Hz, 12.0 Hz, 4H), 7.72-7.66 (m, 2H), 7.64-7.58 (m, 2H), 5.60 (s, 2H), 3.09 (s, 3H); ^{13}C NMR (125 MHz, DMSO-d_6) δ: 170.2, 133.5, 130.1, 130.0, 127.2, 126.3, 126.0, 124.8, 121.9, 29.2, 14.7. ESI-HRMS calcd for $C_{17}H_{16}N_2NaS$ ([M + Na]$^+$) 303.0926, found 303.0922.

Compound **12a,** white solid with 84% yield. ^{1}H NMR (500 MHz, DMSO-d_6) δ: 9.18 (s, 4H), 8.19 (d, J = 10.0 Hz, 1H), 8.09-8.05 (m, 1H), 7.67−7.61 (m, 2H), 7.56−7.53 (m, 1H), 7.36−7.33 (m, 1H), 5.01 (s, 2H), 2.64 (d, J = 10.0 Hz, 3H); ^{13}C NMR (125 MHz, DMSO-d_6) δ: 135.9, 133.1, 131.3, 128.4, 126.9, 126.6, 125.6, 124.8, 33.2, 19.6; ESI-HRMS calcd for $C_{13}H_{14}N_2NaS$ ([M + Na]$^+$) 253.0770, found 254.0764.

Compound **12b,** white solid with 84% yield. ^{1}H NMR (500 MHz, DMSO-d_6) δ: 9.28 (s, 2H), 9.08 (s, 2H), 8.19 (d, J = 10.0 Hz, 1H), 7.99 (d, J = 10.0 Hz, 1H), 7.94 (d, J = 10.0 Hz, 1H), 7.67−7.62 (m, 2H), 7.58 (t, J = 5.0 Hz, 1H), 7.50 (t, J = 5.0 Hz, 1H), 5.02 (s, 2H). ^{13}C NMR (125 MHz, DMSO-d_6) δ: 169.1, 133.6, 130.8, 130.1, 129.1, 128.9, 128.1, 126.8, 126.3, 125.6, 123.8, 32.6; ESI-HRMS calcd for $C_{12}H_{12}N_2NaS$ ([M + Na]$^+$) 239.0613, found 239.0608.

Compound **12c,** white solid with 72% yield.^{1}H NMR (500 MHz, DMSO-d_6) δ: 9.12 (s, 4H), 7.31 (d, J = 10.0 Hz, 2H), 7.19-7.16 (m, 2H), 4.47 (d, J = 20.0 Hz, 2H), 2.28 (d, J = 5.0

Hz, 3H); ^{13}C NMR (125 MHz, DMSO-d_6) *δ*: 169.6, 137.8, 132.3, 129.8, 129.4, 34.6, 21.2; ESI-HRMS calcd for $C_9H_{12}N_2NaS$ ([M + Na]$^+$) 203.0613, found 203.0609.

Compound **12d,** white solid with 76% yield.^1H NMR (500 MHz, DMSO-d_6) *δ*: 9.21 (s, 4H), 8.24 (s, 2H), 7.72 (d, *J* = 10.0 Hz, 2H), 4.72 (s, 2H); ^{13}C NMR (125 MHz, DMSO-d_6) *δ*: 168.9, 147.5, 144.0, 130.7, 124.4, 33.7; ESI-HRMS calcd for $C_8H_9N_3NaO_2S$ ([M + Na]$^+$) 234.0308, found 234.0304.

Compound **12e,** white solid with 78% yield. ^1H NMR (500 MHz, DMSO-d_6) *δ*: 9.14 (s, 4H), 7.96−7.88 (m, 4H), 9.52 (s, 1H), 7.57−7.51 (m, 3H), 4.70−4.66 (m 2H); ^{13}C NMR (125 MHz, DMSO-d_6) *δ*: 168.9, 132.7, 132.6, 132.4, 128.6, 127.8, 127.7, 126.8, 126.7, 126.5, 34.6; ESI-HRMS calcd for $C_{12}H_{12}N_2NaS$ ([M + Na]$^+$) 239.0613, found 239.0610.

Compound **12f,** white solid with 76% yield. ^1H NMR (500 MHz, DMSO-d_6) δ: 8.19 (d, *J* = 8.0 Hz, 1H), 8.09-8.07 (m, 1H), 7.65-760 (m, 2H), 7.51-7.41 (m, 1H), 7.33 (s, 1H), 4.96 (s, 2H), 3.34 (s, 2H), 2.64 (s, 3H).^{13}C NMR (125 MHz, DMSO-d_6) *δ*: 165.2, 135.1, 132.6, 130.8, 128.9, 128.1, 127.8, 126.4, 126.3, 126.1, 125.0, 124.4, 124.2, 32.8, 19.1; ESI-HRMS calcd for $C_{13}H_{15}N_3NaS$ ([M + Na]$^+$) 268.0879, found 268.0868.

Compound **12g,** white solid with 77% yield. ^1H NMR (500 MHz, DMSO-d_6) δ: 10.89 (s, 1H), 9.42-8.86 (m, 2H), 8.19 (d, *J* = 10.0 Hz, 1H), 7.97 (d, *J* = 10.0 Hz, 1H), 7.92 (d, *J* = 10.0 Hz, 1H), 7.64-7.55 (m, 3H), 7.48 (t, *J* = 10.0 Hz, 1H), 5.28−4.83(m, 4H); ^{13}C NMR (125 MHz, DMSO-d_6) *δ*: 133.5, 130.7, 128.9, 127.9, 126.6, 126.2, 125.5, 123.8, 32.8; ESI-HRMS calcd for $C_{12}H_{13}N_3NaS$ ([M + Na]$^+$) 254.0722, found 254.0716.

Compound **12h,** white solid with 81% yield. ^1H NMR (500 MHz, DMSO-d_6) *δ*: 9.41 (s, 3H), 8.21 (d, *J* = 9.5 Hz, 1H), 8.08-8.06 (m, 1H), 7.66-7.60 (m, 2H), 7.54-7.51 (m, 1H), 7.35-7.33 (m, 1H), 5.05-5.01 (m, 2H), 2.91 (s, 3H), 2.64 (s, 3H), 2.07 (s, 1H); ^{13}C NMR (125 MHz, DMSO-d_6) *δ*: 131.0, 130.0, 129.3, 128.9, 127.1, 125.6, 124.0, 19.0; ESI-HRMS calcd for $C_{14}H_{16}N_2NaS$ ([M + Na]$^+$) 267.0926, found 267.0917.

Compound **12i,** white solid with 80% yield.^1H NMR (500 MHz, DMSO-d_6) *δ*: 8.22 (d, *J* = 10.0 Hz, 1H), 8.12 – 8.05 (m, 1H), 7.69 – 7.61 (m, 2H), 7.60 – 7.56 (m, 1H), 7.36 (d, *J* = 5.0 Hz, 1H), 5.14 (s, 2H), 2.65 (s, 3H), 2.22 (s, 3H); ^{13}C NMR (125 MHz, DMSO-d_6) *δ*: 172.3, 135.8, 132.6, 130.9, 128.3, 126.9, 126.6, 126.3, 126.2, 126.2, 125.1, 124.3, 33.3, 24.5, 19.2; ESI-HRMS calcd for $C_{15}H_{16}N_2NaOS$ ([M + Na]$^+$)295.0876, found 295.0869.

Compound **12j,** white solid with 69% yield. ^{1}H NMR (500 MHz, DMSO-d_6) δ: 7.34 (d, J = 10.0 Hz, 2H), 7.18 (d, J = 5.0 Hz, 2H), 4.65 (s, 2H), 2.27 (s, 3H), 2.21 (s, 3H); ^{13}C NMR (125 MHz, DMSO-d_6) δ: 172.2, 169.5, 137.7, 130.7, 129.5, 129.2, 34.6, 24.5, 20.7; ESI-HRMS calcd for $C_{11}H_{14}N_2NaOS$ ([M + Na]$^+$)245.0719, found 245.0714.

Compound **12k,** white solid with 71% yield. ^{1}H NMR (500 MHz, DMSO-d_6) δ: 8.45 (d, J = 10.0 Hz, 1H), 8.01 (t, J = 10.0 Hz, 2H), 7.86–7.77 (m, 1H), 7.64 (d, J = 10.0 Hz, 2H), 4.73 (s, 2H). ^{13}C NMR (125 MHz, DMSO-d_6) δ: 156.6, 146.5, 137.9, 130.3, 128.1, 128.0, 127.0, 126.9, 121.3, 36.4; ESI-HRMS calcd for $C_{11}H_{12}N_4NaS$ ([M + Na]$^+$) 255.0675, found 255.0668.

Compound **12l,** white solid with 78% yield. ^{1}H NMR (500 MHz, DMSO-d_6) δ: 10.16 (s, 1H), 9.80 (s, 1H), 9.15 (s, 1H), 8.47 (d, J = 10.0 Hz, 1H), 8.03 (d, J = 10.0 Hz, 1H), 7.98 (d, J = 10.0 Hz, 1H), 7.83 (t, J = 5.0 Hz, 1H), 7.67−7.63 (m, 2H), 7.84−7.83 (m, 1H), 2.97 (d, J = 5.0 Hz, 3H). ^{13}C NMR (125 MHz, DMSO-d_6) δ: 166.8, 156.5, 146.5, 138.0, 130.5, 128.1, 128.0, 127.1, 127.0, 121.3, 36.8, 30.6; ESI-HRMS calcd for $C_{12}H_{13}N_3NaS$ ([M + Na]$^+$) 254.0722, found 254.0715.

Compound **13a,** yellow solid with 76% yield. ^{1}H NMR (500 MHz, DMSO-d_6) δ: 8.44 (d, J = 10.0 Hz, 4H), 7.68 (t, J = 5.0 Hz, 2H), 7.62 (t, J = 5.0 Hz, 2H), 5.54 (s, 2H), 3.43 (s, 5H), 3.10 (s, 3H). ^{13}C NMR (100 MHz, DMSO-d_6) δ:166.1, 133.3, 130.1, 130.0, 127.1, 126.3, 126.0, 124.9, 123.1, 29.3, 14.8; ESI-HRMS calcd for $C_{17}H_{17}N_3NaS$ ([M + Na$^+$]$^+$) 318.1035, found 318.1025.

Compound **13b,** yellow solid with 88% yield. ^{1}H NMR (500 MHz, DMSO-d_6) δ: 9.33 (s, 2H), 9.17 (s, 2H), 8.45 (d, J = 10 Hz, 4H), 7.70 (t, J = 5.0 Hz, 2H), 7.63 (t, J = 5.0 Hz, 2H), 5.57 (s, 2H), 3.10 (s, 3H). ^{13}C NMR (125 MHz, DMSO-d_6) δ: 169.7, 133.1, 129.7, 129.5, 126.7, 125.9, 125.5, 124.3, 121.4, 28.7, 14.3; ESI-HRMS calcd for $C_{19}H_{19}N_2OS$ ([M + H$^+$]$^+$) 323.1213, found 323.1238.

Compound **13c,** yellow solid with 69% yield. ^{1}H NMR (400 MHz, DMSO-d_6) δ: 8.43 (d, J = 8.0 Hz, 2H), 8.36 (d, J = 8.0 Hz, 2H), 8.19 (s, 5H), 7.69 – 7.59 (m, 4H), 5.26 (s, 2H), 3.09 (s, 3H); ^{13}C NMR (100 MHz, DMSO-d_6) δ: 164.4, 162.8, 130.0, 130.0, 126.9, 126.3, 125.9, 125.0, 124.4, 29.3, 14.7. ESI-HRMS calcd for $C_{18}H_{18}N_4NaS$ ([M + Na$^+$]$^+$) 345.1144, found 345.1138.

Compound **13d,** yellow solid with 82% yield. ^{1}H NMR (500 MHz, DMSO-d_6) δ: 9.38 (s, 2H), 9.21 (s, 2H), 8.56-8.54 (m, 4H), 7.79-7.77 (m, 4H), 5.61 (s, 2H). ^{13}C NMR (125 MHz,

DMSO-d_6) δ: 166.2, 133.0, 129.7, 129.5, 126.7, 125.8, 125.5, 124.5, 121.8, 30.7, 29.3, 14.3. ESI-HRMS calcd for $C_{18}H_{18}N_2NaS$ ([M + Na]$^+$) 317.1083, found 317.1076.

Compound **13e,** yellow solid with 79% yield. ^{1}H NMR (500 MHz, DMSO-d_6) δ: 9.03 (s, 2H), 8.47 – 8.33 (m, 4H), 7.72 – 7.56 (m, 4H), 5.75 – 5.61 (m, 2H), 5.32 (s, 1H), 3.29 (s, 3H), 3.09 (s, 3H).; ^{13}C NMR (125 MHz, DMSO-d_6) δ: 170.8, 167.1, 133.4, 132.8, 129.8, 129.7, 129.5, 126.9, 126.7, 125.9, 125.5, 124.3, 121.5, 121.1, 42.3, 30.3, 28.8, 14.3. ESI-HRMS calcd for $C_{18}H_{19}N_3NaS$ ([M + Na$^+$]$^+$) 332.1192, found 332.1182.

Compound **13f,** yellow solid with 85% yield. ^{1}H NMR (500 MHz, DMSO-d_6) δ: 8.51 (d, J = 10.0 Hz, 1H), 8.48-8.32 (m, 3H), 7.69 (t, J = 5.0 Hz, 2H), 7.63 (t, J = 5.0 Hz, 2H), 5.68 (s, 1H), 3.31 (s, 3H), 2.11 (d, J = 30.0 Hz, 5H), 1.97-1.82 (m, 2H). ^{13}C NMR (125 MHz, DMSO-d_6) δ: 129.7, 129.5, 126.6, 125.8, 125.5, 124.5, 19.1, 14.2; ESI-HRMS calcd for $C_{20}H_{21}N_3NaS$ ([M + Na$^+$]$^+$) 358.1348, found 358.1338.

Compound **13g,** yellow solid with 81% yield. ^{1}H NMR (500 MHz, DMSO-d_6) δ: 12.34 (s, 1H), 8.39 (t, J = 6.5 Hz, 2H), 8.32 (t, J = 6.5 Hz, 2H),7.56-7.58 (m, 4H), 7.21 (s, 2H), 5.27 (s, 3H). ^{13}C NMR (125 MHz, DMSO-d_6) δ: 138.7, 131.3, 129.7, 129.4, 126.8, 126.1, 125.7, 125.4, 124.9, 31.9, 14.3. ESI-HRMS calcd for $C_{19}H_{16}N_2NaS$ ([M + Na]$^+$) 327.0926, found 327.0917.

Compound **13h,** yellow solid with 84% yield. ^{1}H NMR (500 MHz, DMSO-d_6) δ: 8.41 (dd, J = 10.0 Hz, 20.0 Hz, 4H), 7.66-7.57 (m, 4H), 5.49 (d, J = 20.0 Hz, 2H), 5.29 (s, 1H), 3.09 (s, 3H). ^{13}C NMR (125 MHz, DMSO-d_6) δ: 131.7, 129.5, 129.5, 126.5, 126.4, 125.7, 125.4, 125.1, 124.6, 85.8, 27.2, 14.1; ESI-HRMS calcd for $C_{20}H_{16}N_2NaO_2S$ ([M + Na$^+$]$^+$) 371.0825, found 371.0810.

Compound **13i,** yellow solid with 78% yield. ^{1}H NMR (400 MHz, DMSO-d_6) δ: 8.45 – 8.40 (m, 4H), 8.22 (s, 1H), 7.64 – 7.57 (m, 4H), 5.55 (s, 2H), 4.34 (q, J = 8.0 Hz, 2H), 1.32 (t, J = 8.0 Hz, 3H); ^{13}C NMR (100 MHz, DMSO-d_6) δ: 143.0, 132.3, 129.5, 129.5, 127.4, 126.5, 126.3, 125.7, 125.4, 124.9, 124.6, 61.0, 31.6, 14.3. ESI-HRMS calcd for $C_{22}H_{20}N_2NaO_2S$ ([M + Na$^+$]$^+$) 399.1138, found 399.1129.

Compound **13j,** yellow solid with 85% yield.^{1}H NMR (500 MHz, DMSO-d_6) δ: 11.52 (s, 1H), 9.86 (s, 1H), 9.46 (s, 1H),8.56 (d, J = 10.0 Hz, 2H), 8.46 (d, J = 10.0 Hz, 2H), 7.71 (t, J = 5.0 Hz, 2H), 7.64 (t, J = 5.0 Hz, 2H), 7.55 (m, 2H), 7.44 (d, J = 10.0 Hz, 3H), 5.74 (s, 2H), 3.11 (s, 3H). ^{13}C NMR (125 MHz, DMSO-d_6) δ: 135.1, 133.2, 130.0, 129.8, 129.5, 128.3,

126.7, 125.9, 125.6, 125.4, 124.5, 29.7, 14.3; ESI-HRMS calcd for $C_{23}H_{20}N_2NaS$ ([M + Na$^+$]$^+$) 379.1245, found 379.1228.

Compound **13k,** yellow solid with 82% yield. ^{1}H NMR (500 MHz, DMSO-d_6) δ: 12.34 (s, 1H), 8.39 (t, J = 6.5 Hz, 2H), 8.32 (t, J = 6.5 Hz, 2H),7.56-7.58 (m, 4H), 7.21 (s, 2H), 5.27 (s, 3H). ^{13}C NMR (125 MHz, DMSO-d_6) δ: 138.7, 131.3, 129.7, 129.4, 126.8, 126.1, 125.7, 125.4, 124.9, 31.9, 14.3. ESI-HRMS calcd for $C_{19}H_{16}N_2NaS$ ([M + Na]$^+$) 327.0926, found 327.0917.^{1}H NMR (400 MHz, DMSO-d_6) δ: 8.48 – 8.40 (m, 4H), 7.68 – 7.57 (m, 4H), 7.48 – 7.32 (m, 10H), 7.29 – 7.20 (m, 2H), 5.60 (s, 2H), 3.08 (s, 3H). ^{13}C NMR (100 MHz, DMSO-d_6) δ: 140.0, 133.4, 130.4, 130.2, 130.0, 129.8, 128.9, 128.8, 127.5, 127.1, 126.3, 126.0, 125.1, 124.9, 124.1, 123.7, 122.7, 31.2, 14.8; ESI-HRMS calcd for $C_{29}H_{24}N_2NaS$ ([M + Na$^+$]$^+$) 455.1558, found 455.1541.

Compound **13l,** yellow solid with 85% yield. ^{1}H NMR (500 MHz, DMSO-d_6) δ: 9.75 (s, 1H), 9.51 (s, 1H), 9.28 (s, 1H), 8.46(dd, J = 10.0 Hz, 15.0 Hz, 4H), 7.69 (t, J = 5.0 Hz, 2H), 7.62 (t, J = 5.0 Hz, 2H), 5.60 (s, 2H), 3.10 (s, 3H), 1.61-1.55 (m, 2), 1.29 (s, 1H), 0.90-0.87 (m, 3H). ^{13}C NMR (100 MHz, DMSO-d_6) δ: 165.9, 133.3, 130.2, 130.0, 127.1, 126.3, 126.0, 125.1, 122.6, 44.6, 31.4, 30.1, 27.9, 26.3, 22.5, 14.8, 14.4; ESI-HRMS calcd for $C_{23}H_{28}N_2NaS$ ([M + Na$^+$]$^+$) 387.1865, found 387.1858.

Compound **14a,** yellow solid with 71% yield. ^{1}H NMR (500 MHz, DMSO-d_6) δ: 9.38 (s, 4H), 8.45 (dd, J = 10.0 Hz, 15.0 Hz, 4H), 7.73–7.58 (m, 4H), 5.57 (s, 2H), 3.66 (q, J = 10.0 Hz, 2H), 1.36 (t, J = 10.0 Hz, 3H). ^{13}C NMR (125 MHz, DMSO-d_6) δ: 169.8, 139.1, 129.8, 128.5, 126.7, 125.8, 125.3, 124.5, 121.7, 28.7, 20.7, 15.7; ESI-HRMS calcd for $C_{18}H_{18}N_2NaS$ ([M + Na$^+$]$^+$) 317.1083, found 317.1071.

Compound **14b,** yellow solid with 75% yield. ^{1}H NMR (500 MHz, DMSO-d_6) δ: 10.09 (s, 1H), 9.71 (s, 1H), 9.34 (s, 1H), 8.50 (d, J = 10.0 Hz, 2H,), 8.42 (d, J = 10.0 Hz, 2H), 7.76 (t, J = 5.0 Hz, 2H), 7.62 (t, J = 5.0 Hz, 2H), 5.65 (s, 2H), 3.66 (q, J = 5.0 Hz, 2H), 3.00 (d, J = 5.0 Hz, 3H), 1.36 (t, J = 5.0 Hz, 3H). ^{13}C NMR (125 MHz, DMSO-d_6) δ: 166.1, 139.0, 129.9, 128.6, 126.6,125.7 125.3, 124.7, 122.3, 30.7, 29.4, 20.7, 15.7; ESI-HRMS calcd for $C_{19}H_{20}N_2NaS$ ([M + Na$^+$] $^+$) 331.1239, found 331.1229.

Compound **14c,** yellow solid with 81% yield. ^{1}H NMR (500 MHz, DMSO-d_6) δ: 9.39 (s, 2H), 8.52 – 8.30 (m, 4H), 7.73 – 7.56 (m, 4H), 5.69 (s, 1H), 5.37 (s, 2H), 3.63 (q, J = 7.5 Hz, 2H), 3.47 (s, 3H), 1.33 (t, J = 7.5 Hz, 3H). ^{13}C NMR (125 MHz, DMSO-d_6) δ: 170.7, 167.0,

139.4, 138.8, 129.9, 128.6, 126.8, 126.6, 125.7, 125.3, 124.5, 122.0, 121.5, 42.4, 30.5, 28.9, 20.7, 15.7; ESI-HRMS calcd for $C_{19}H_{21}N_3NaS$ ([M + Na$^+$]$^+$) 346.1348, found 346.1357.

Compound **14d,** yellow solid with 81% yield. ^{1}H NMR (400 MHz, DMSO-d_6) δ: 8.41 (d, J = 8.0 Hz, 2H), 8.36 (d, J = 8.0 Hz, 2H), 7.69 – 7.57 (m, 5H), 7.18 (s, 2H), 7.01 (s, 2H), 5.25 (s, 2H), 3.65 (q, J = 8.0 Hz, 2H), 1.35 (t, J = 8.0 Hz, 3H); ^{13}C NMR (100 MHz, DMSO-d_6) δ: 187.6, 163.9, 162.4, 161.4, 138.2, 129.7, 128.6, 126.4, 125.7, 125.2, 124.7, 124.2, 28.8, 20.7, 15.8. 28.8, 20.7, 15.8; ESI-HRMS calcd for $C_{19}H_{20}N_4NaS$ ([M + Na$^+$] $^+$) 359.1301, found 359.1291.

Compound **14e,** yellow solid with 85% yield. ^{1}H NMR (400 MHz, DMSO-d_6) δ: 8.54 – 8.39 (m, 4H), 7.73 – 7.60 (m, 4H), 5.67 (s, 2H), 3.67 (d, J = 8.0 Hz, 2H), 2.26 (s, 3H), 1.36 (t, J = 8.0 Hz, 3H); ^{13}C NMR (100 MHz, DMSO-d_6) δ: 181.7, 172.0, 139.2, 130.1, 129.4, 128.5, 126.9, 126.7, 126.5, 125.8, 125.7, 125.6, 125.4, 125.3, 125.1, 124.6, 23.8, 20.8, 15.7; ESI-HRMS calcd for $C_{20}H_{20}N_2NaOS$ ([M + Na$^+$]$^+$) 359.1189, found 359.1179.

Compound **14f,** yellow solid with 77% yield. ^{1}H NMR (500 MHz, DMSO-d_6) δ: 10.03 (t, J = 5.0 Hz, 1H), 9.75 (s, 1H), 9.52 (s, 1H), 8.52 (d, J = 10.0 Hz, 2H), 8.41 (d, J = 10.0 Hz, 2H), 7.76 (t, J = 5.0 Hz, 2H), 7.61 (t, J = 5.0 Hz, 2H), 5.67 (s, 2H), 3.65 (q, J = 10.0 Hz, 2H), 1.59 (t, J = 5.0 Hz, 3H), 1.36-1.28 (m , 10H), 0.89-0.86 (m, 3H). ^{13}C NMR (125 MHz, DMSO-d_6) δ: 165.2, 138.9, 129.9, 128.5, 126.5, 125.7, 125.2, 124.8, 122.5, 43.9, 30.8, 29.5, 27.4, 25.8, 22.0, 20.7, 15.7, 13.9; ESI-HRMS calcd for $C_{24}H_{30}N_2NaS$ ([M + Na$^+$] $^+$) 401.2022, found 401.2017.

Compound **14g,** yellow solid with 73% yield. ^{1}H NMR (400 MHz, DMSO-d_6) δ: 8.63 (d, J = 8.0 Hz, 2H), 8.43 (d, J = 8.0 Hz, 2H), 7.87 (s, 1H), 7.76 (s, 3H), 7.71 – 7.61 (m, 4H), 5.82 (s, 2H), 3.66 (q, J = 8.0 Hz, 2H), 1.36 (t, J = 8.0 Hz, 3H); ^{13}C NMR (100 MHz, DMSO-d_6) δ: 139.2, 131.1, 130.0, 129.5, 128.6, 126.7, 125.8, 125.3, 124.9, 122.4, 122.0, 30.0, 20.8, 15.7; ESI-HRMS calcd for $C_{25}H_{21}F_3N_2NaS$ ([M + Na$^+$]$^+$) 461.1270, found 461.1262.

Compound **14h,** yellow solid with 74% yield. ^{1}H NMR (500 MHz, DMSO-d_6) δ: 8.47 – 8.40 (m, 4H), 7.70 – 7.65 (m, 2H), 7.64 – 7.59 (m, 2H), 5.57 (s, 2H), 3.63 – 3.56 (m, 2H), 1.73 (q, J = 7.5 Hz, 2H), 1.10 (t, J = 7.5 Hz, 3H); ^{13}C NMR (125 MHz, DMSO-d_6) δ: 183.9, 169.8, 137.8, 129.8, 129.0, 126.8, 125.8, 125.6, 124.5, 121.8, 29.5, 28.7, 24.6, 14.4; ESI-HRMS calcd for $C_{19}H_{20}N_2NaS$ ([M + Na]$^+$) 331.1239, found 331.1227.

Compound **14i,** yellow solid with 87% yield. ^{1}H NMR (500 MHz, DMSO-d_6) δ: 9.84 (s, 1H), 9.58 (s, 1H), 9.23 (s, 1H), 8.71 (s, 1H), 8.47 (d, J = 10.0 Hz, 2H), 8.16 (d, J = 10.0 Hz, 2H), 7.68 (t, J = 10.0 Hz, 2H), 7.59 (t, J = 10.0 Hz, 2H), 5.62 (s, 2H), 3.00 (d, J = 5.0 Hz, 3H). ^{13}C NMR (125 MHz, DMSO-d_6) δ: 166.1, 131.0, 129.9, 129.3, 128.9, 127.1, 125.6, 123.9, 123.8, 30.7, 29.0; ESI-HRMS calcd for $C_{17}H_{16}N_2NaS$ ([M + Na$^+$]$^+$) 303.0926, found 303.0916.

Compound **14j,** yellow solid with 89% yield. ^{1}H NMR (500 MHz, DMSO-d_6) δ: 8.70 (s, 1H), 8.43 (d, J = 10.0 Hz, 2H), 8.16 (d, J = 10.0 Hz, 2H), 7.68 (t, J = 10.0 Hz, 2H), 7.58 (t, J = 10.0 Hz, 2H), 5.55 (s, 2H). ^{13}C NMR (125 MHz, DMSO-d_6) δ: 131.0, 129.8, 129.3, 127.1, 125.5, 123.9; ESI-HRMS calcd for $C_{16}H_{15}N_3NaS$ ([M + Na$^+$]$^+$) 304.0879, found 304.0870.

Compound **14k,** yellow solid with 81% yield. ^{1}H NMR (400 MHz, DMSO-d_6) δ: 8.73-8.71 (s, 1H), 8.65 – 8.31 (m, 2H), 8.16 (d, J = 8.3 Hz, 2H), 7.69 (t, J = 7.3 Hz, 2H), 7.59 (t, J = 7.3 Hz, 2H), 5.84 – 5.41 (m, 2H), 2.35 – 1.61 (m, 6H). ^{13}C NMR (100 MHz, DMSO-d_6) δ: 164.4, 131.6, 130.5, 129.8, 129.5, 127.7, 126.1, 124.5, 29.6, 25.5, 19.7; ESI-HRMS calcd for $C_{19}H_{20}N_3S$ ([M + H$^+$]$^+$) 322.1372, found 322.1326.

Compound **14l,** yellow solid with 66% yield. ^{1}H NMR (500 MHz, DMSO-d_6) δ: 8.74 (s, 1H), 8.48 (d, J = 10.0 Hz, 2H), 8.18 (d, J = 10.0 Hz, 2H), 7.69 (t, J = 10.0 Hz, 2H), 7.60 (t, J = 10.0 Hz, 2H), 5.73 (s, 2H), 2.26 (s, 3H); ^{13}C NMR (125 MHz, DMSO-d_6) δ: 172.2, 131.0, 130.2, 129.4, 129.3, 129.1, 127.4, 125.7, 125.6, 124.0, 123.8, 122.4, 29.2, 24.5, 23.8; ESI-HRMS calcd for $C_{18}H_{16}N_2NaS$ ([M + Na]$^+$)331.0876, found 331.0867.

Compound **14m,** yellow solid with 84% yield. ^{1}H NMR (500 MHz, DMSO-d_6) δ: 9.38 (s, 2H), 9.21 (s, 2H), 8.56-8.54 (m, 4H), 7.79–7.77 (m, 4H), 5.61 (s, 2H). ^{13}C NMR (125 MHz, DMSO-d_6) δ: 169.2, 130.6, 129.7, 128.1, 128.1, 127.6, 125.0, 124.7, 124.4, 28.6; ESI-HRMS calcd for $C_{16}H_{13}BrN_2NaS$ ([M + Na$^+$] $^+$) 366.9875, found 366.9868.

Compound **14n,** yellow solid with 83% yield. ^{1}H NMR (500 MHz, DMSO-d_6) δ: 9.88 (s, 1H), 9.61 (s, 1H), 9.25 (s, 1H), 8.61−8.50 (m, 4H), 7.83−7.71 (m, 4H), 5.64 (s, 2H), 2.99 (d, J = 5.0 Hz, 3H). ^{13}C NMR (125 MHz, DMSO-d_6) δ: 166.6, 131.5, 130.5, 129.8, 129.4, 127.7, 126.1, 124.5, 124.3; ESI-HRMS calcd for $C_{17}H_{15}BrN_2NaS$ ([M + Na]$^+$) 381.0032, found 381.0025.

Compound **14o,** yellow solid with 83% yield. ^{1}H NMR (400 MHz, DMSO-d_6) δ: 8.52 (d, J = 8.0 Hz, 4H), 7.77 (dd, J = 4.0 Hz, 8.0 Hz, 4H), 5.57 (s, 2H). ^{13}C NMR (100 MHz, DMSO-

d_6) δ: 130.9, 129.8, 129.2, 127.0, 125.5, 123.8; ESI-HRMS calcd for $C_{16}H_{14}BrN_3NaS$ ([M + Na]$^+$) 381.9984, found 381.9975.

Compound **14p,** yellow solid with 70% yield.^{1}H NMR (500 MHz, DMSO-d_6) δ: 10.04 (s, 1H), 9.77 (s, 1H), 9.36 (s, 1H), 7.33 (t, *J* = 5.0 Hz, 2H), 7.27 (d, *J* = 10.0 Hz, 2H), 7.04 (d, *J* = 10.0 Hz, 2H), 7.00 (t, *J* = 5.0 Hz, 2H), 4.88 (s, 2H), 3.65 (s, 2H). ^{13}C NMR (125 MHz, DMSO-d_6) δ: 168.7, 138.3, 129.8, 126.1, 120.7, 116.4, 114.8, 72.6, 43.7; ESI-HRMS calcd for $C_{15}H_{14}N_4NaS$ ([M + Na]$^+$) 305.0831, found 305.0828.

Compound **14q,** yellow solid with 77% yield.^{1}H NMR (500 MHz, DMSO-d_6) δ: 11.04 (s, 1H), 9.81 (s, 1H), 9.69 (s, 1H), 9.37, (s, 1H), 7.40 (d, *J* = 10.0 Hz, 2H), 7.31 (t, *J* = 5.0 Hz, 2H), 7.02 (m, 4H), 3.62 (s, 2H). ^{13}C NMR (125 MHz, DMSO-d_6) δ: 170.9, 137.4, 129.4, 125.7, 120.4, 119.4, 114.5, 67.8, 47.8; ESI-HRMS calcd for $C_{15}H_{13}N_3NaS$ ([M + Na]$^+$) 290.0722, found 290.0715.

Compound **14r,** yellow solid with 64% yield. ^{1}H NMR (500 MHz, DMSO-d_6) δ: 10.85 (s, 1H), 10.26 (s, 1H), 9.68 (s, 1H), 7.41 (d, *J* = 10.0 Hz, 2H), 7.33−7.31 (m, 2H), 7.04−6.98 (m, 4H), 3.59 (s, 2H), 3.48 (q, *J* = 5.0 Hz, 2H), 1.63−1.60 (m, 2H), 1.37−1.28 (m, 6H), 0.9 (t, *J* = 5.0 Hz, 4H). ^{13}C NMR (125 MHz, DMSO-d_6) δ: 165.2, 131.0, 129.9, 129.3, 128.9, 127.1, 125.5, 124.0, 123.9, 44.0, 30.8, 27.4, 25.8, 22.0, 13.9; ESI-HRMS calcd for $C_{21}H_{25}N_3NaS$ ([M + Na]$^+$) 374.1661, found 374.1653.

Compound **14s,** white solid with 73% yield. ^{1}H NMR (500 MHz, DMSO-d_6) δ: 9.71 (d, *J* = 50.0 Hz, 2H), 9.22 (d, *J* = 25.0 Hz, 2H), 7.66−7.61 (m, 2H), 7.30−7.26 (m, 2H), 7.23 (s, 4H), 6.84 (d, *J* = 50.0 Hz, 1H), 3.62 (q, *J* = 10.0 Hz, 10.0 Hz, 3H), 2.93−2.88 (m, 2H); ^{13}C NMR (125 MHz, DMSO-d_6) δ = 167.2, 143.5, 141.1, 137.7, 135.5, 131.3, 129.9, 129.4, 127.2, 126.8, 126.0, 125.4, 71.2, 32.6, 31.9; ESI-HRMS calcd for $C_{16}H_{16}N_2NaS$ ([M + Na]$^+$) 291.0926, found 291.0921.

Table of contents

Figure S1-S2. NMR spectra of compound XI-011.

Figure S3-S4. NMR spectra of compound 12a.

Figure S5-S6. NMR spectra of compound 12b.

Figure S7-S8. NMR spectra of compound 12c.

Figure S9-S10. NMR spectra of compound 12d.

Figure S11-S12. NMR spectra of compound 12e.

Figure S13-S14. NMR spectra of compound 12f.

Figure S15-S16. NMR spectra of compound 12g.

Figure S17-S18. NMR spectra of compound 12h.

Figure S19-S20. NMR spectra of compound 12i.

Figure S21-S22. NMR spectra of compound 12j.

Figure S23-S24. NMR spectra of compound 12k.

Figure S25-S26. NMR spectra of compound 12l.

Figure S27-S28. NMR spectra of compound 13a.

Figure S29-S30. NMR spectra of compound 13b.

Figure S31-S32. NMR spectra of compound 13c.

Figure S33-S34. NMR spectra of compound 13d.

Figure S35-S36. NMR spectra of compound 13e.

Figure S37-S38. NMR spectra of compound 13f.

Figure S39-S40. NMR spectra of compound 13g.

Figure S41-S42. NMR spectra of compound 13h.

Figure S43-S44. NMR spectra of compound 13i.

Figure S45-S46. NMR spectra of compound 13j.

Figure S47-S48. NMR spectra of compound 13k.

Figure S49-S50. NMR spectra of compound 13l.

Figure S51-S52. NMR spectra of compound 14a.

Figure S53-S54. NMR spectra of compound 14b.

Figure S55-S56. NMR spectra of compound 14c.

Figure S57-S58. NMR spectra of compound 14d.

Figure S59-S60. NMR spectra of compound 14e.

Figure S61-S62. NMR spectra of compound 14f.

Figure S63-S64. NMR spectra of compound 14g.

Figure S65-S66. NMR spectra of compound 14h.

Figure S67-S68. NMR spectra of compound 14i.

Figure S69-S70. NMR spectra of compound 14j.

Figure S71-S72. NMR spectra of compound 14k.

Figure S73-S74. NMR spectra of compound 14l.

Figure S75-S76. NMR spectra of compound 14m.

Figure S77-S78. NMR spectra of compound 14n.

Figure S79-S80. NMR spectra of compound 14o.

Figure S81-S82. NMR spectra of compound 14p.

Figure S83-S84. NMR spectra of compound 14q.

Figure S85-S86. NMR spectra of compound 14r.

Figure S87-S88. NMR spectra of compound 14s.

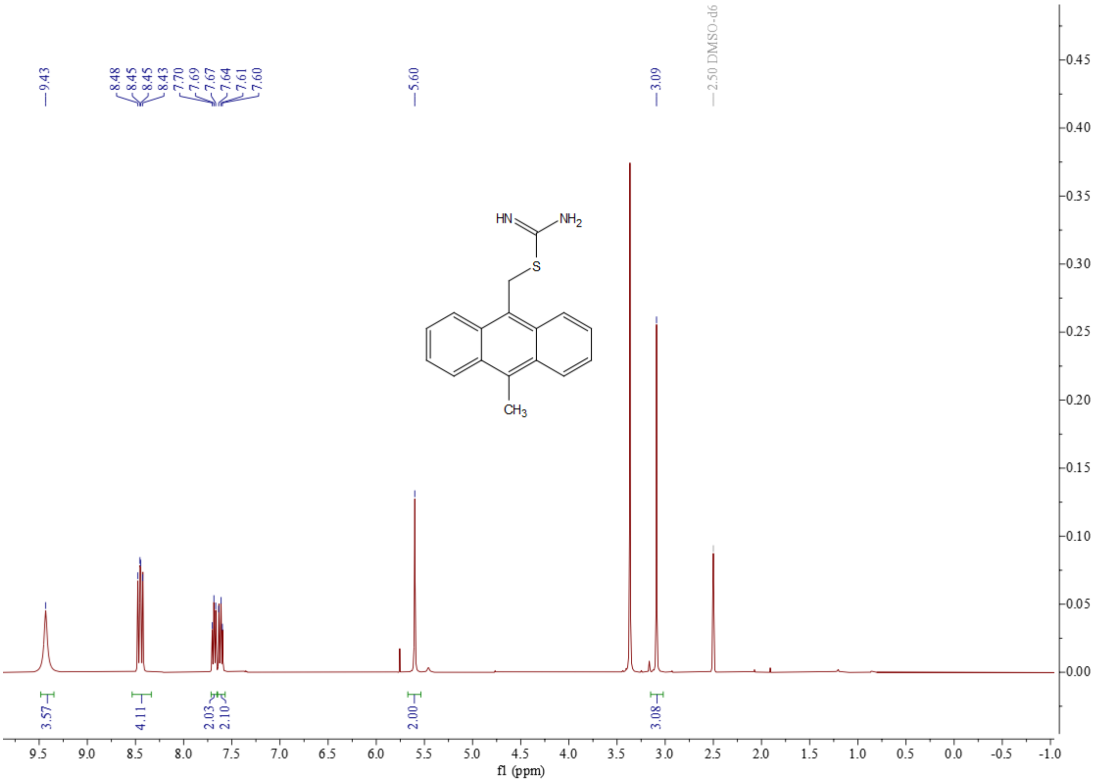

Figure S1 ^{1}H NMR spectrum of **XI-011**

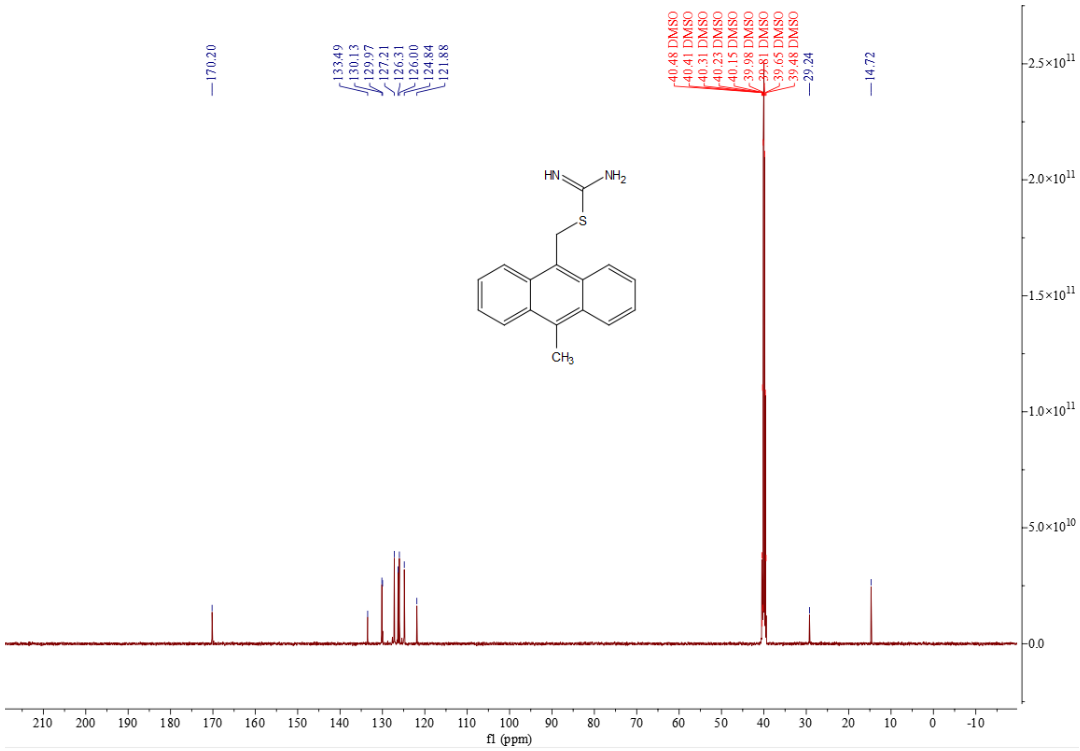

Figure S2 ^{13}C NMR spectrum of **XI-011**

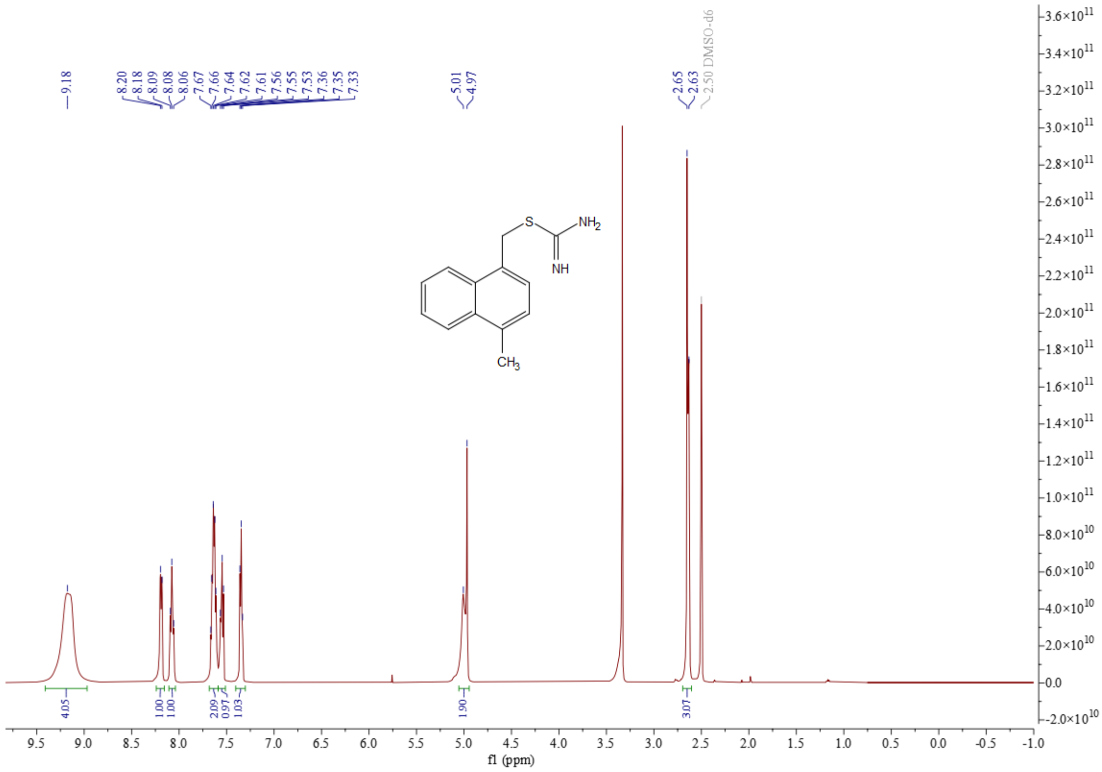

Figure S3 ^{1}H NMR spectrum of **12a**

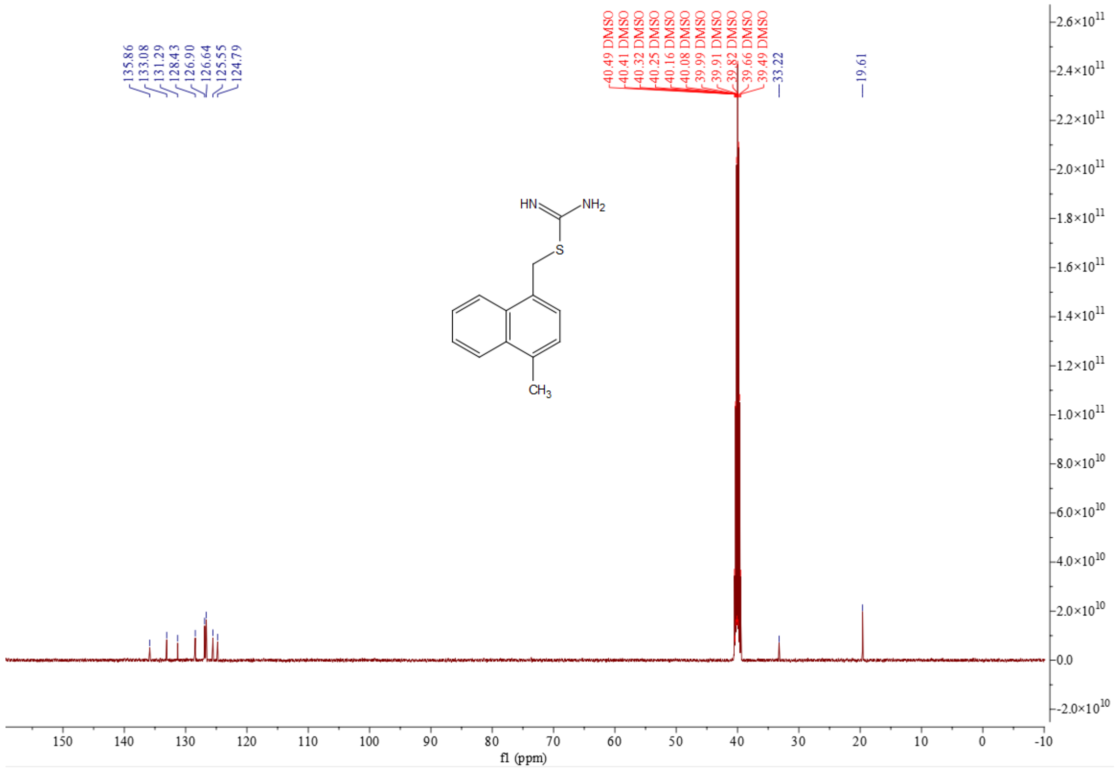

Figure S4 ^{13}C NMR spectrum of **12a**

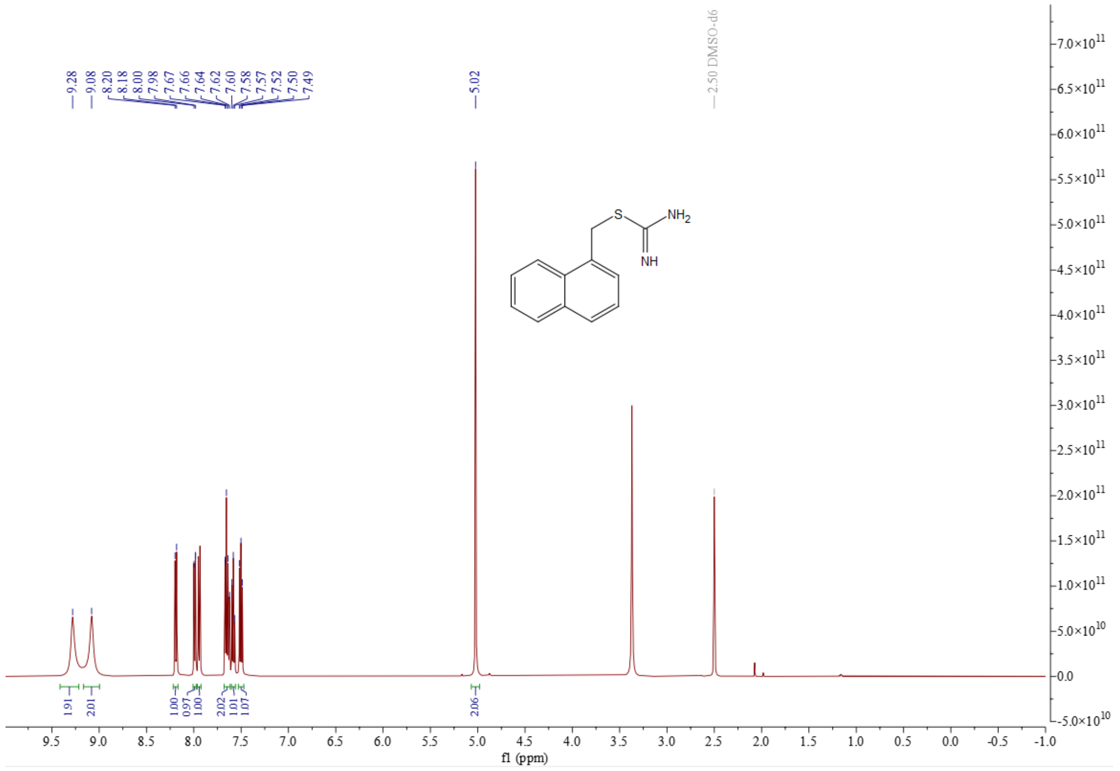

Figure S5 ^{1}H NMR spectrum of **12b**

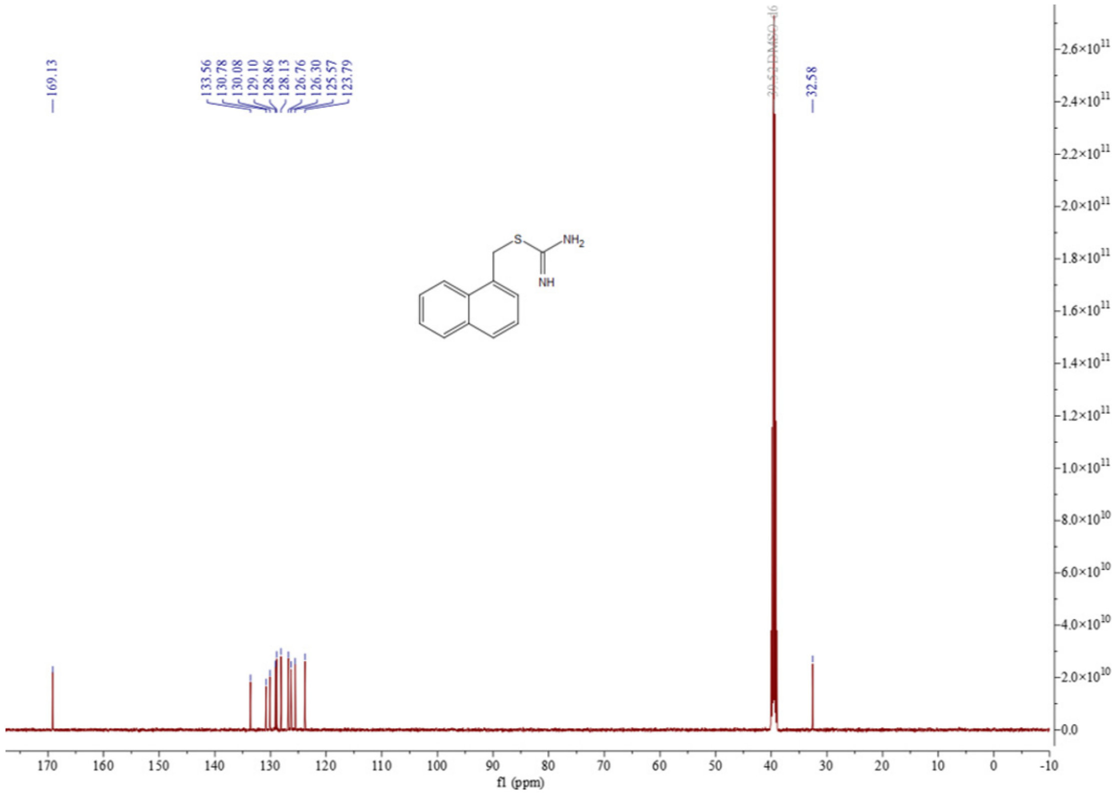

Figure S6 ^{13}C NMR spectrum of **12b**

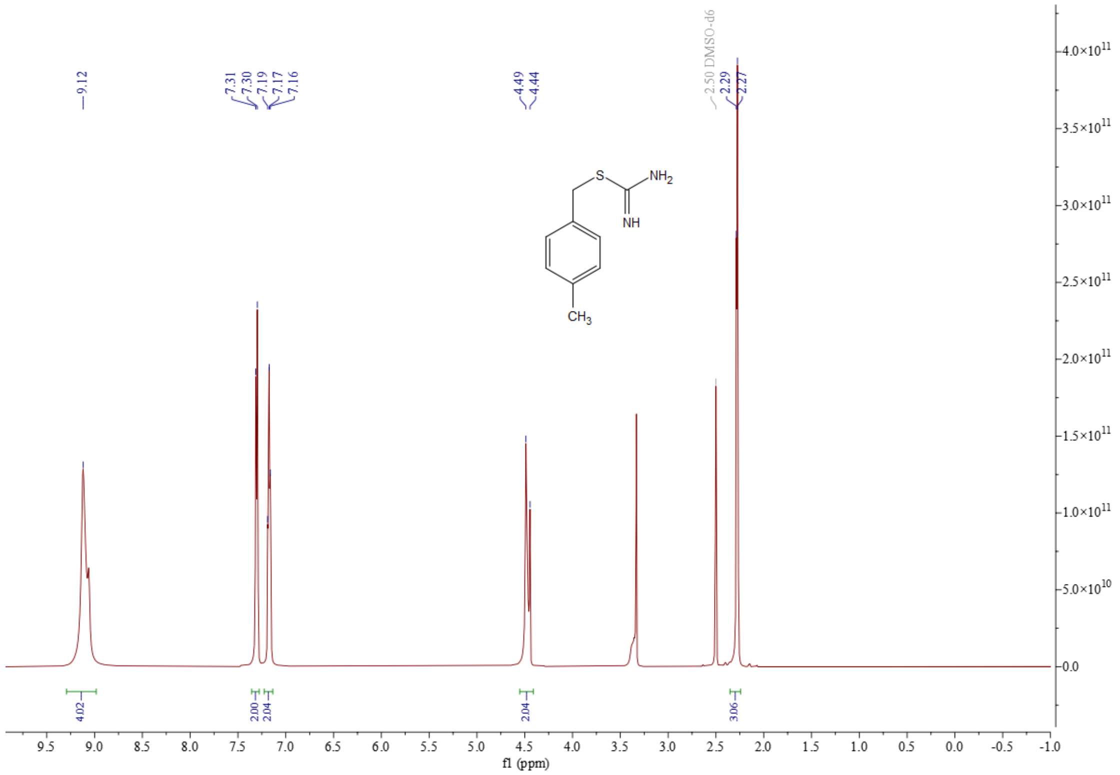

Figure S7 ^{1}H NMR spectrum of **12c**

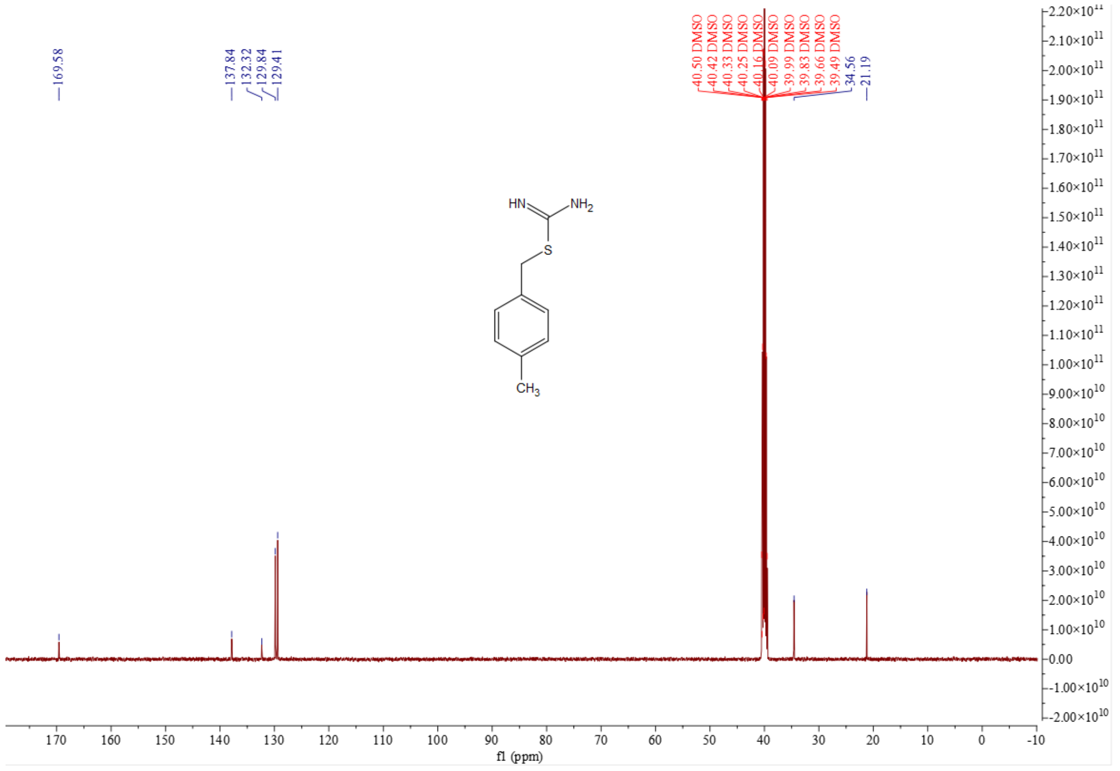

Figure S8 ^{13}C NMR spectrum of **12c**

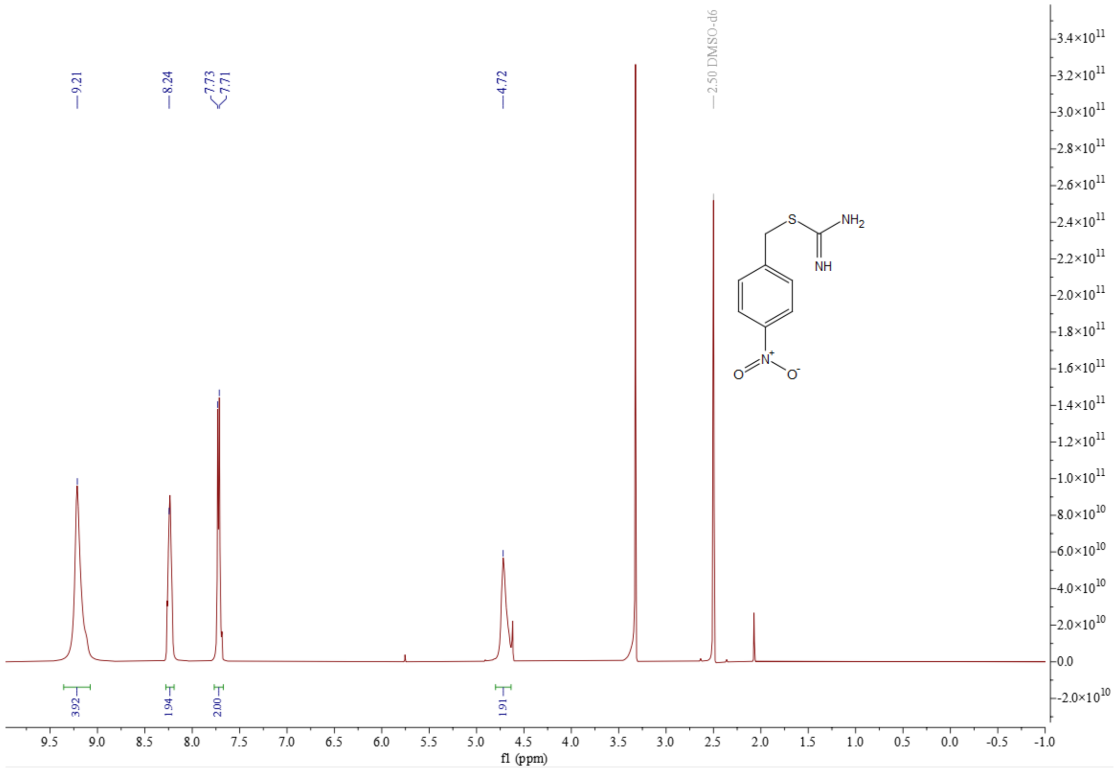

Figure S9 ^{1}H NMR spectrum of **12d**

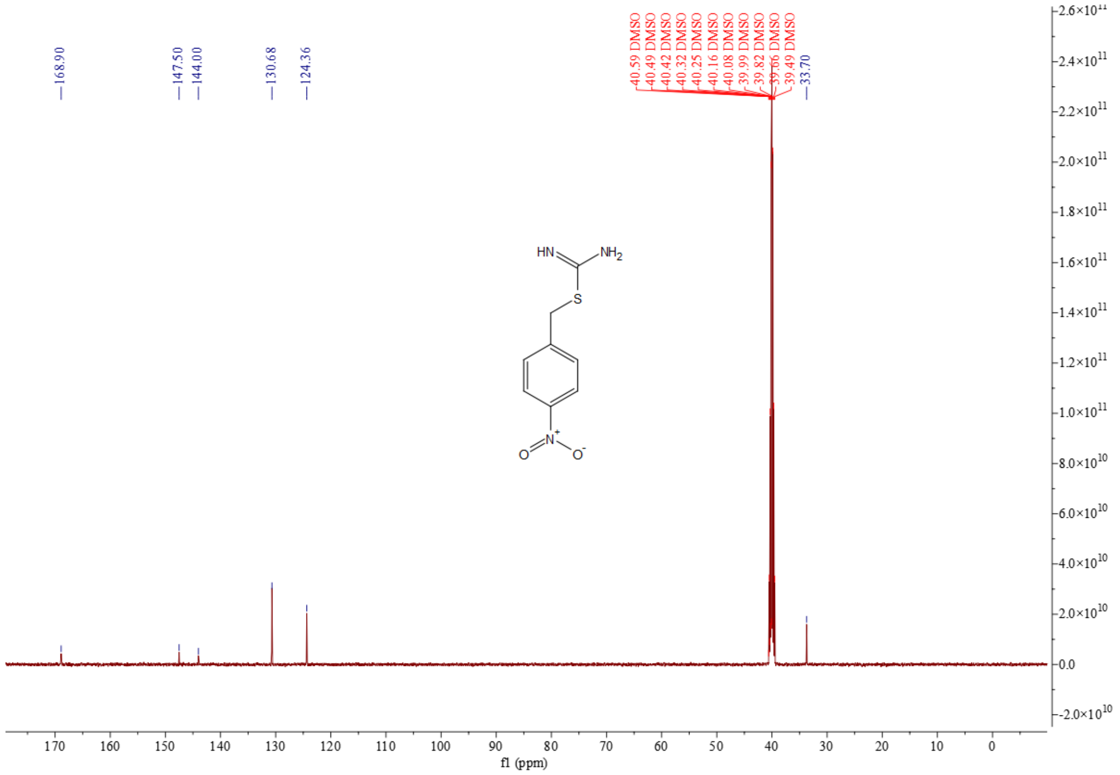

Figure S10 ^{13}C NMR spectrum of **12d**

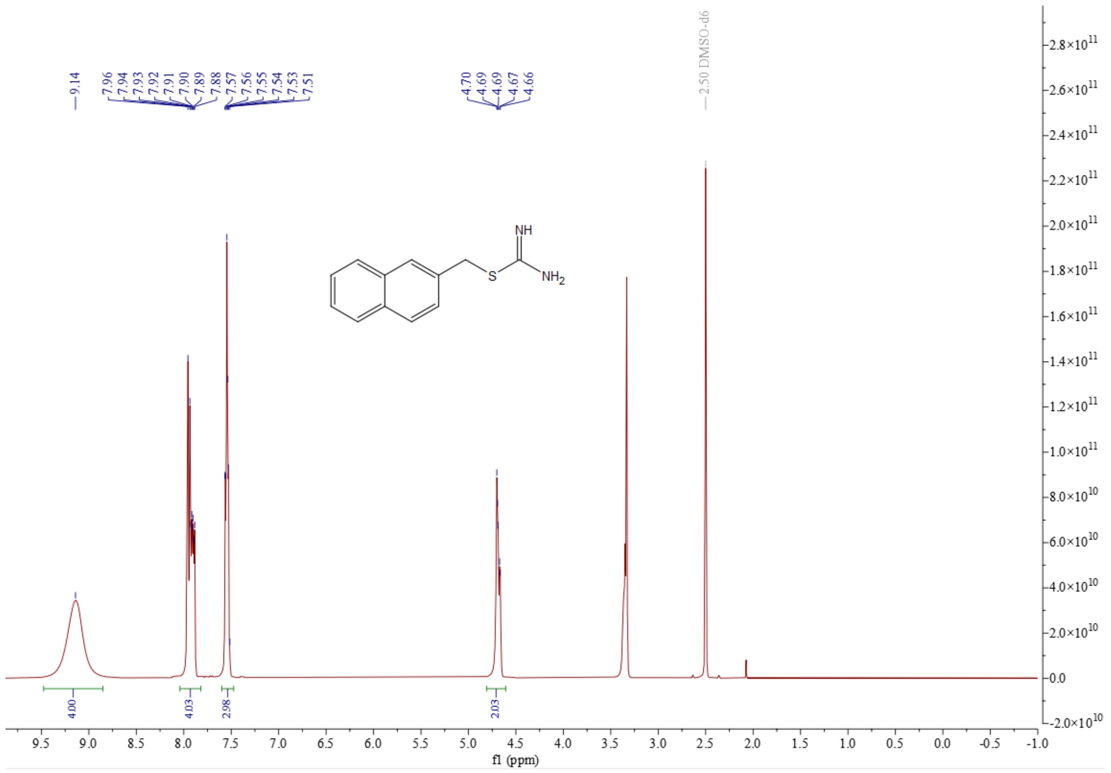

Figure S11 ^{1}H NMR spectrum of **12e**

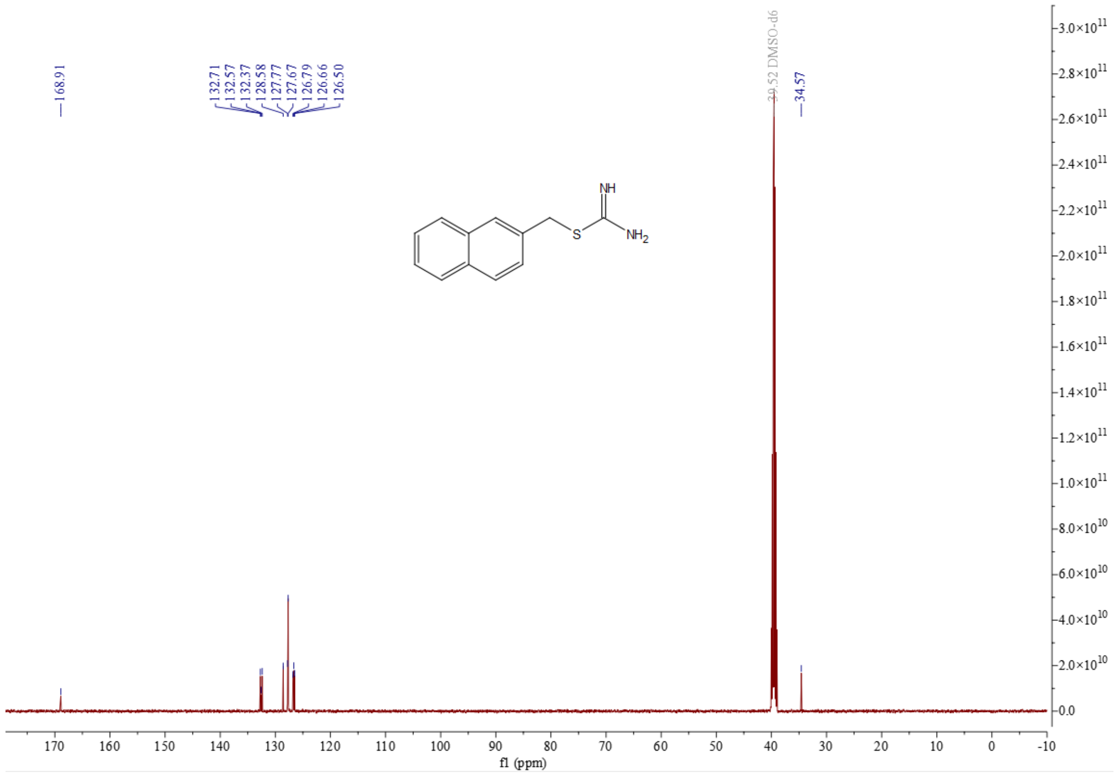

Figure S12 ^{13}C NMR spectrum of **12e**

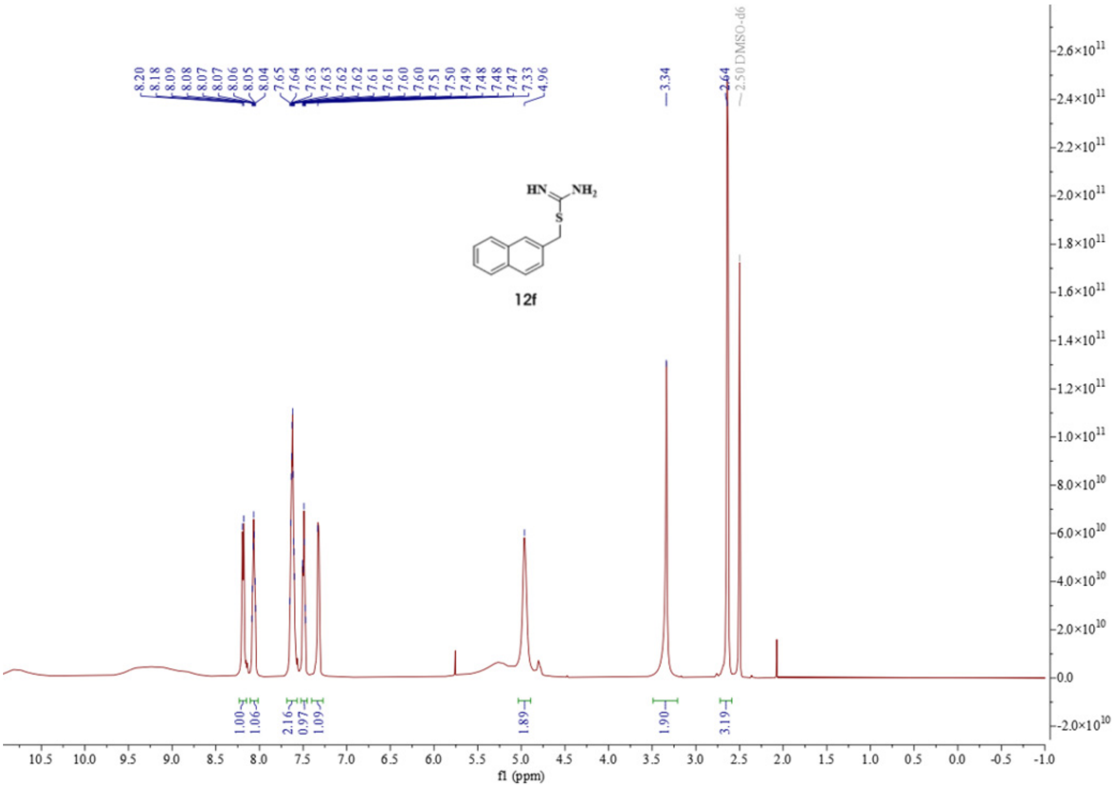

Figure S13 ^{1}H NMR spectrum of **12f**

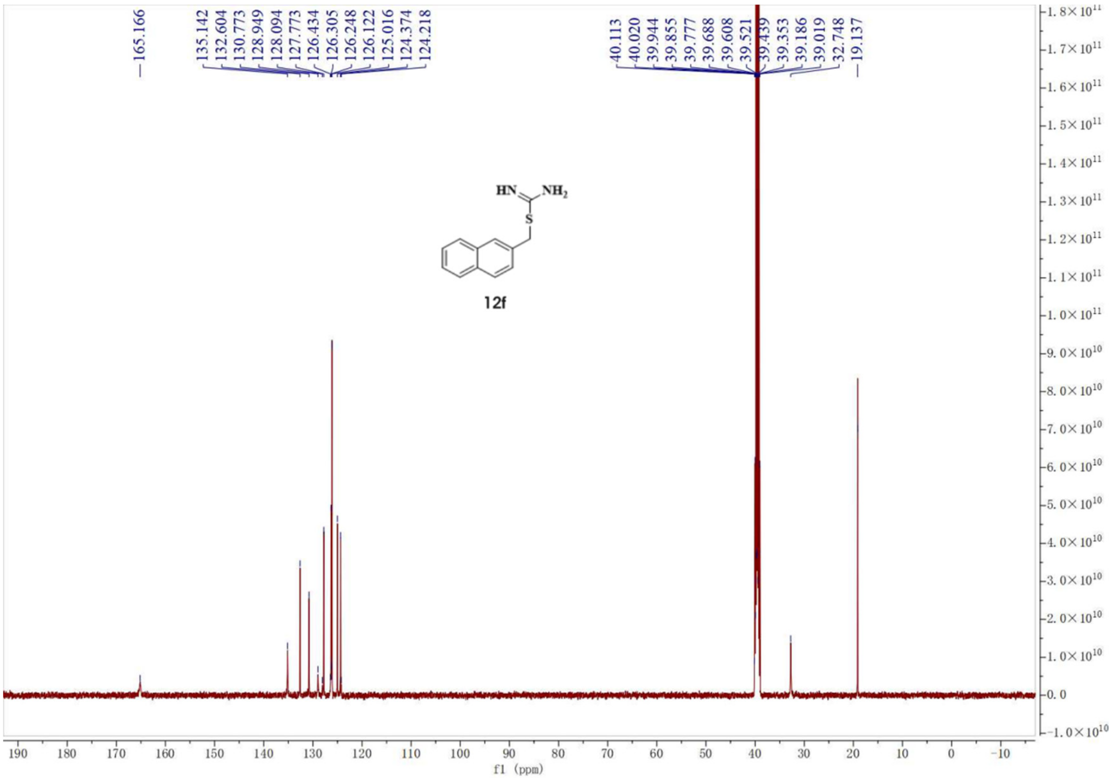

Figure S14 ^{13}C NMR spectrum of **12f**

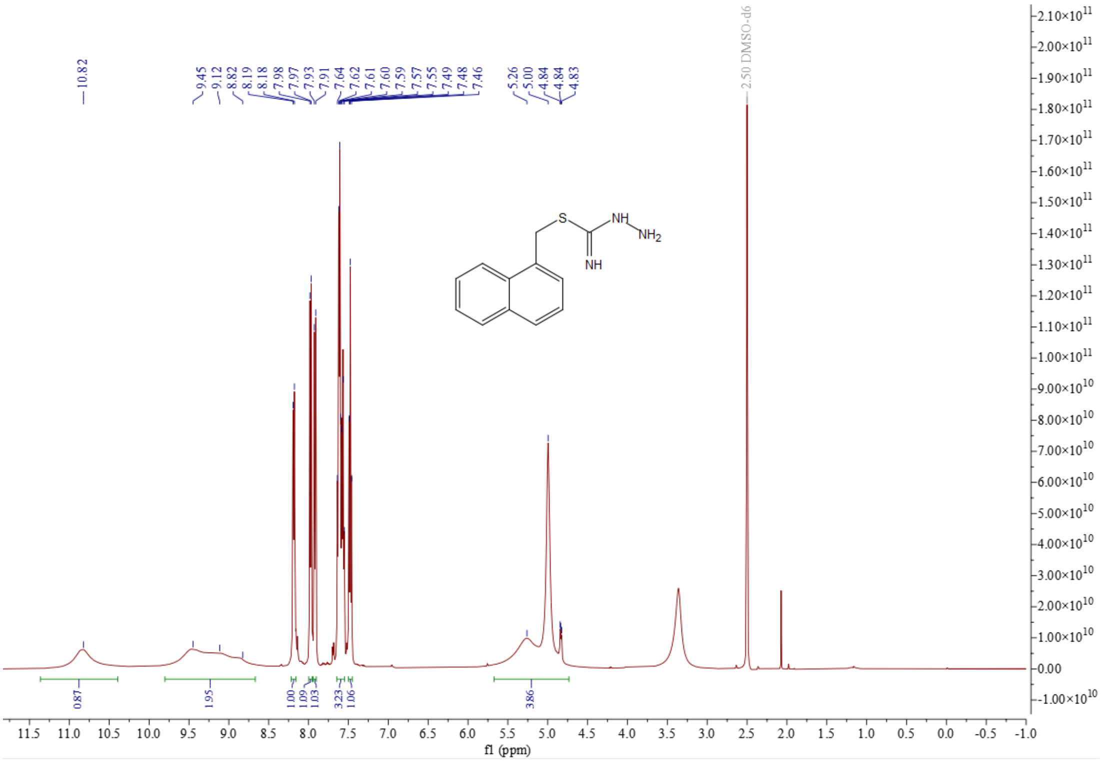

Figure S15 ^{1}H NMR spectrum of **12g**

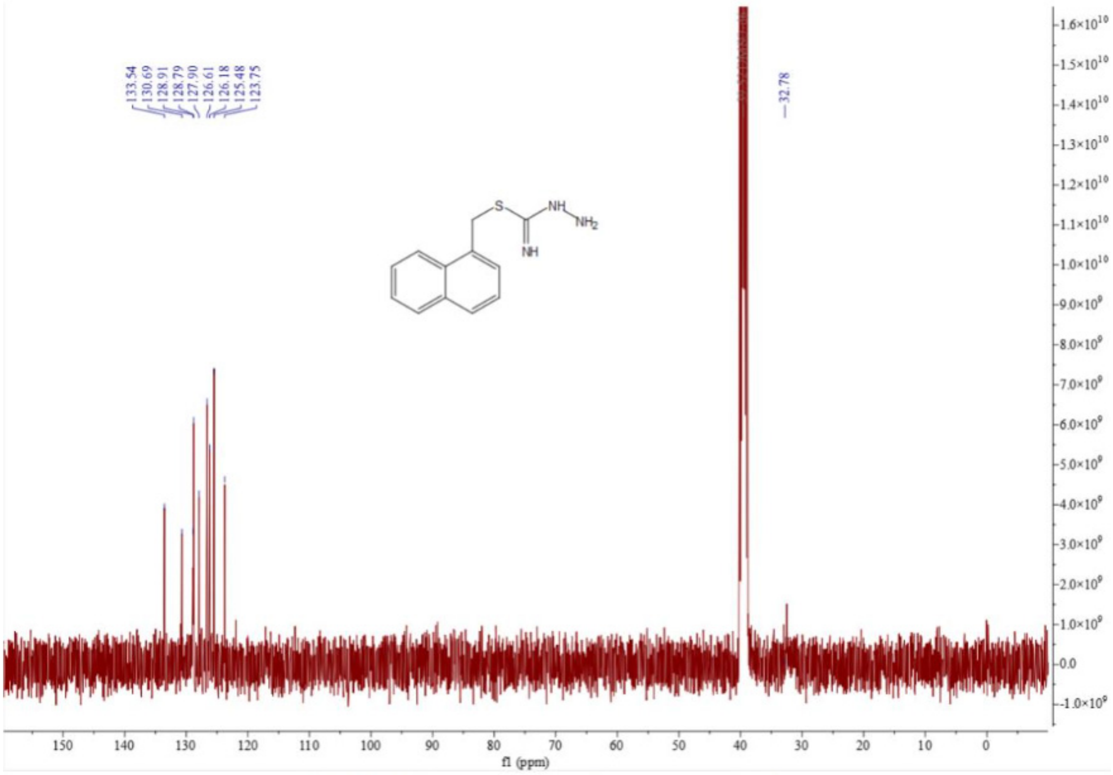

Figure S16 ^{13}C NMR spectrum of **12g**

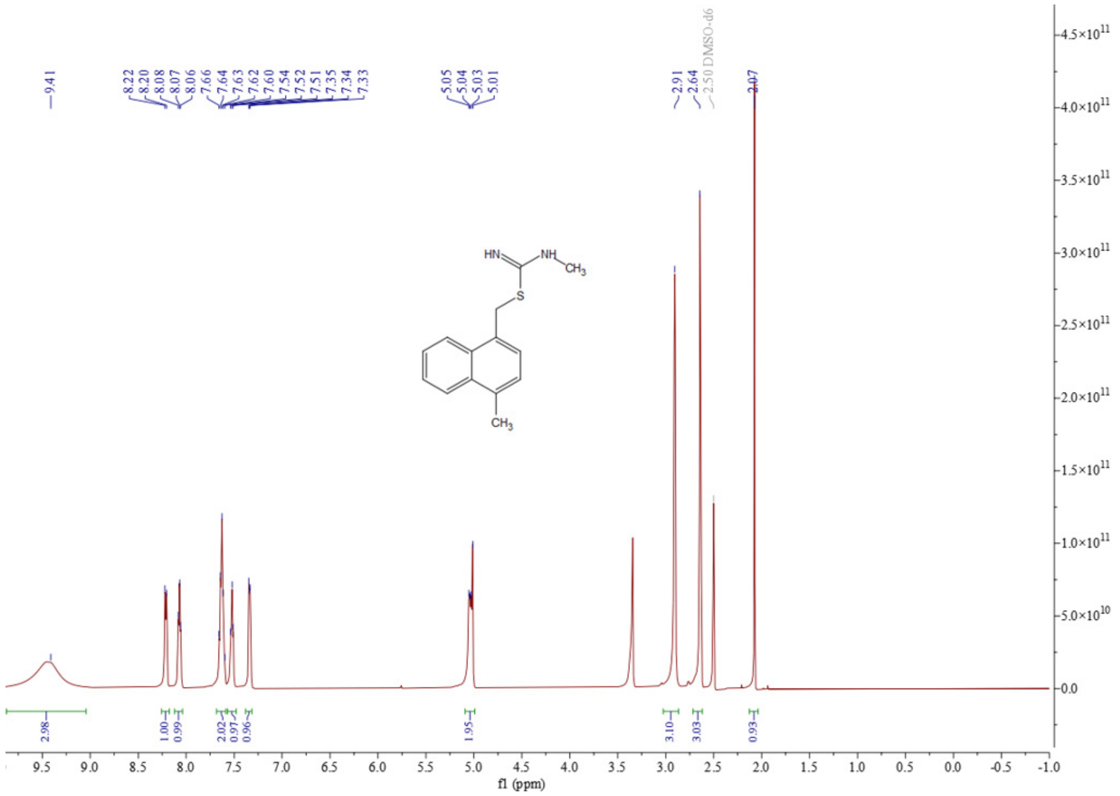

Figure S17 ^{1}H NMR spectrum of **12h**

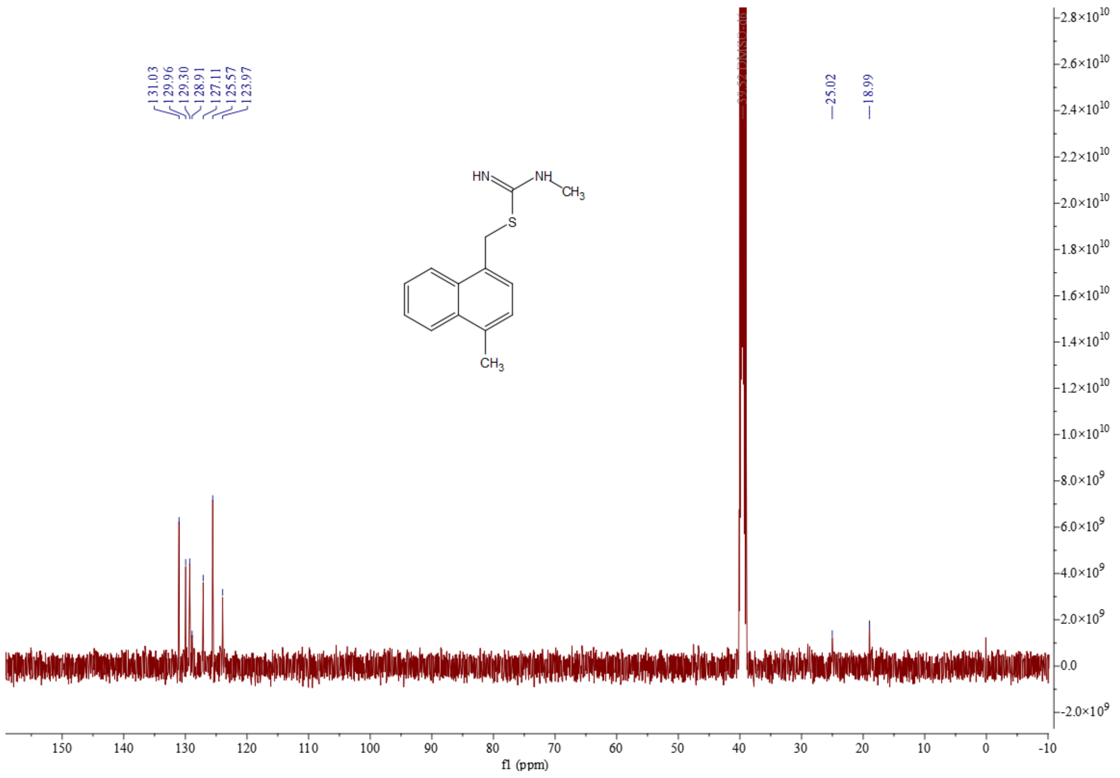

Figure S18 ^{13}C NMR spectrum of **12h**

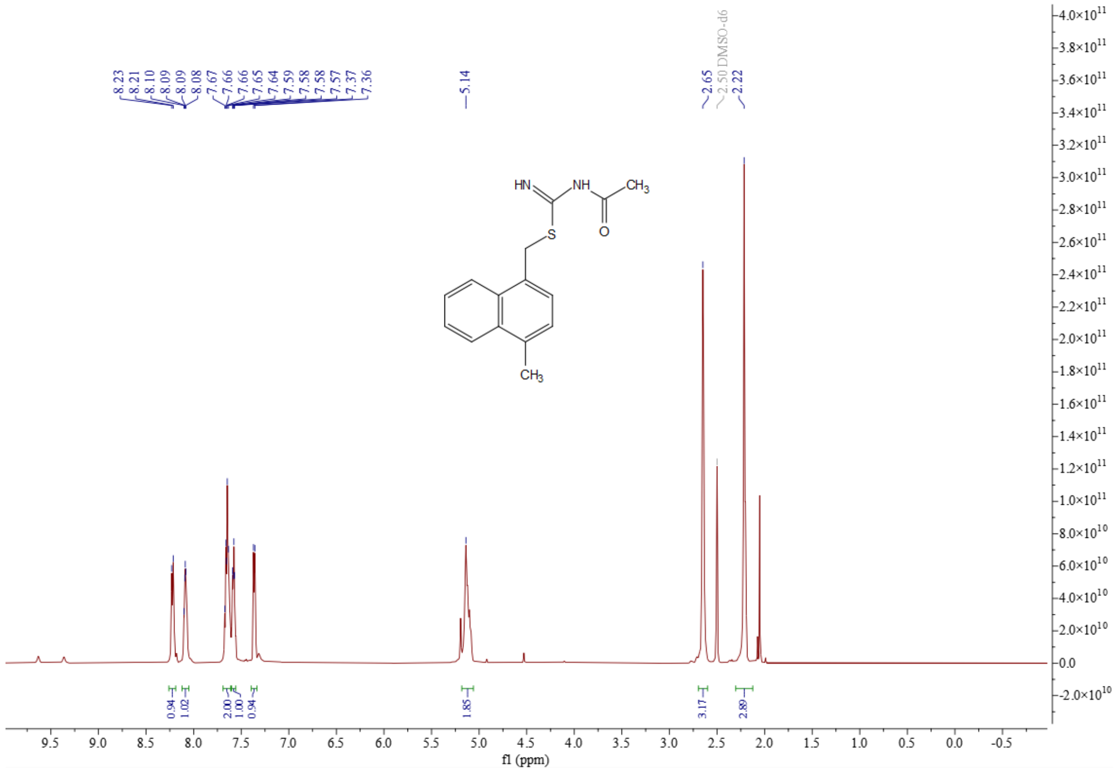

Figure S19 ^{1}H NMR spectrum of **12i**

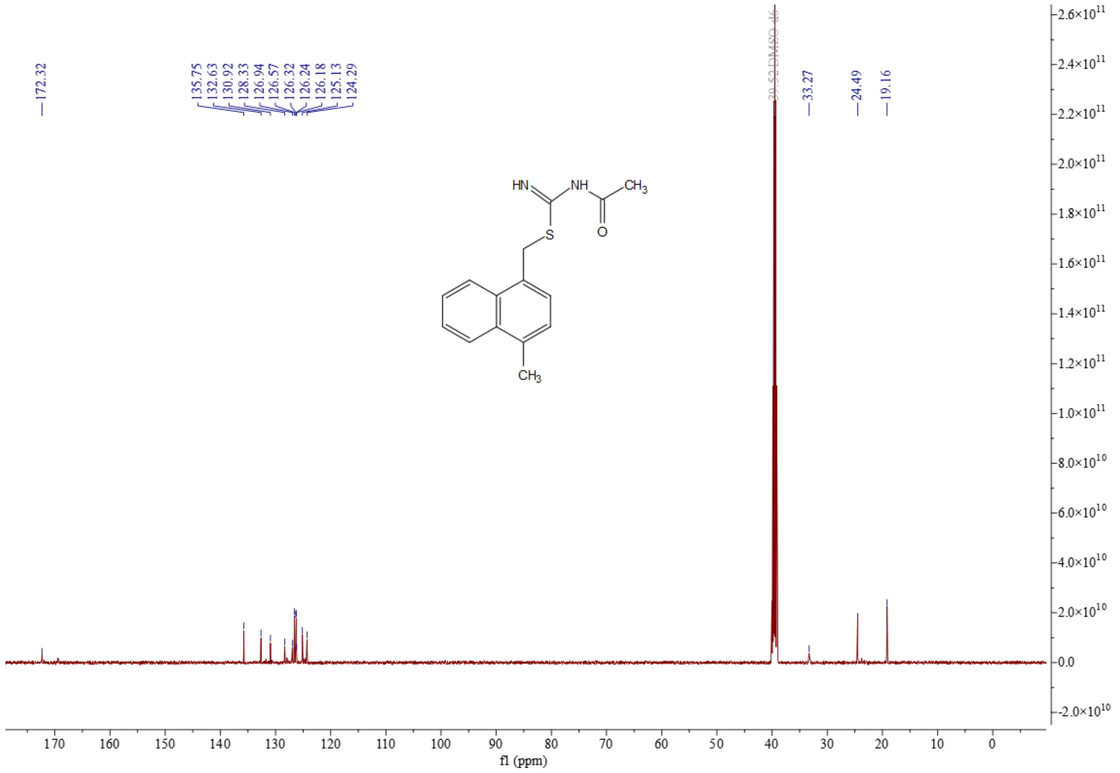

Figure S20 ^{13}C NMR spectrum of **12i**

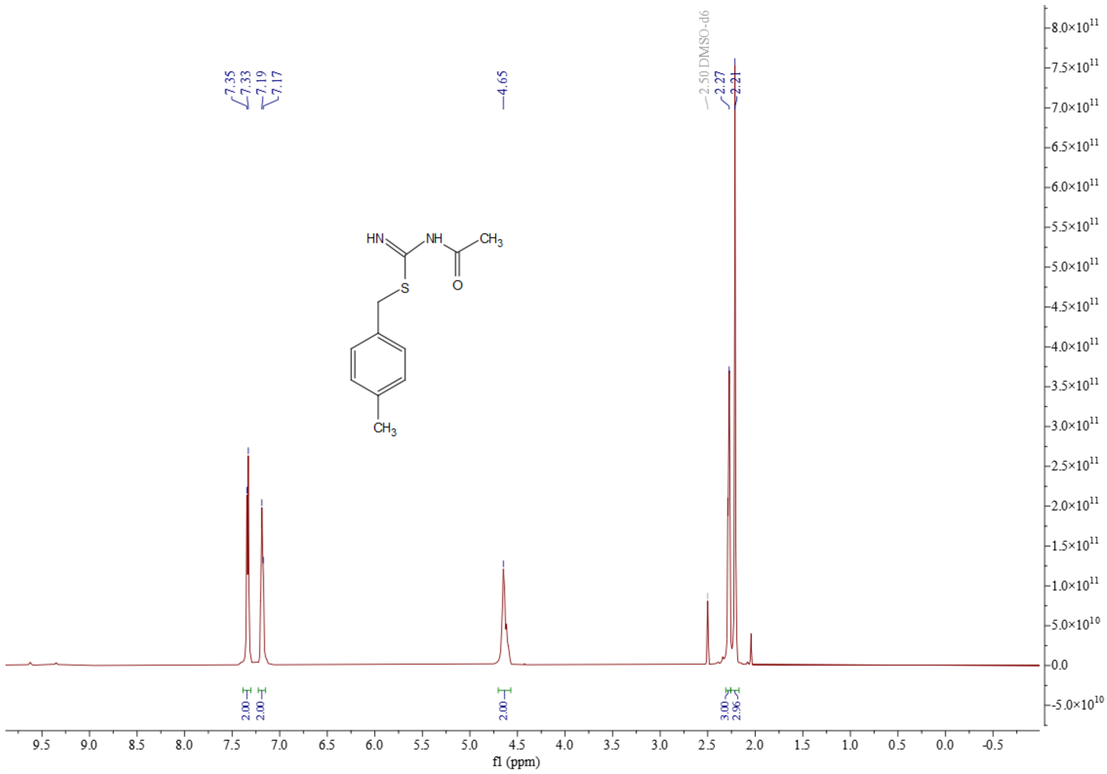

Figure S21 ^{1}H NMR spectrum of **12j**

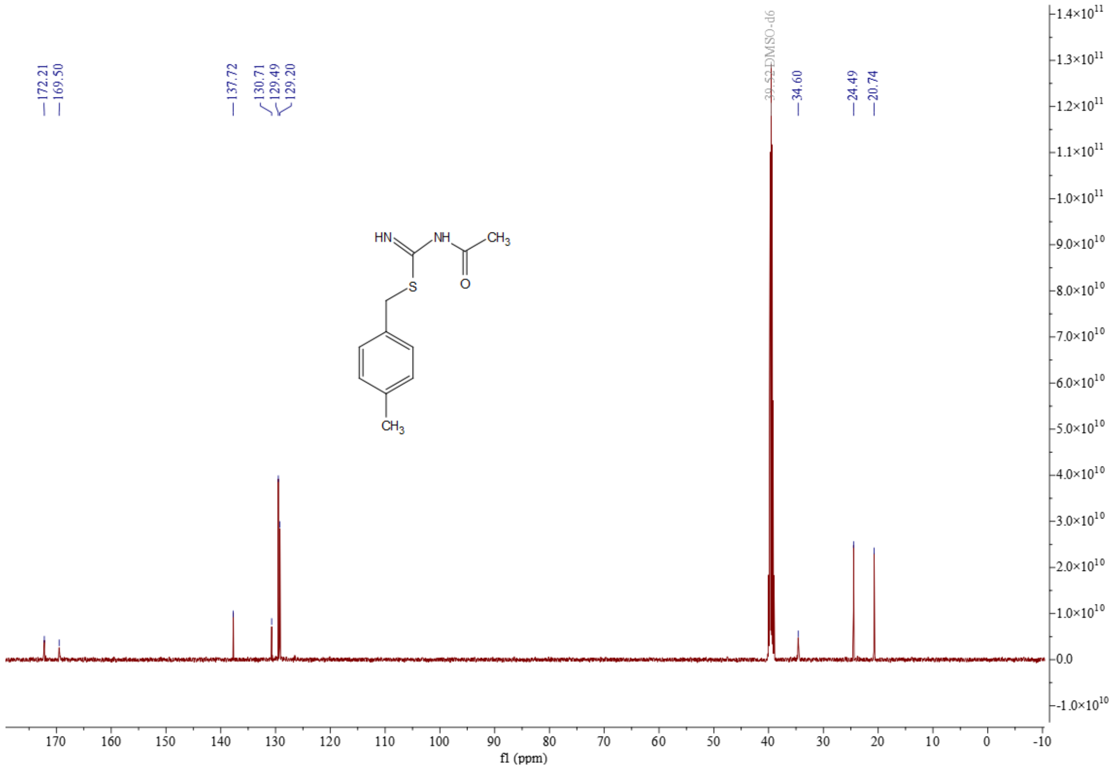

Figure S22 ^{13}C NMR spectrum of **12j**

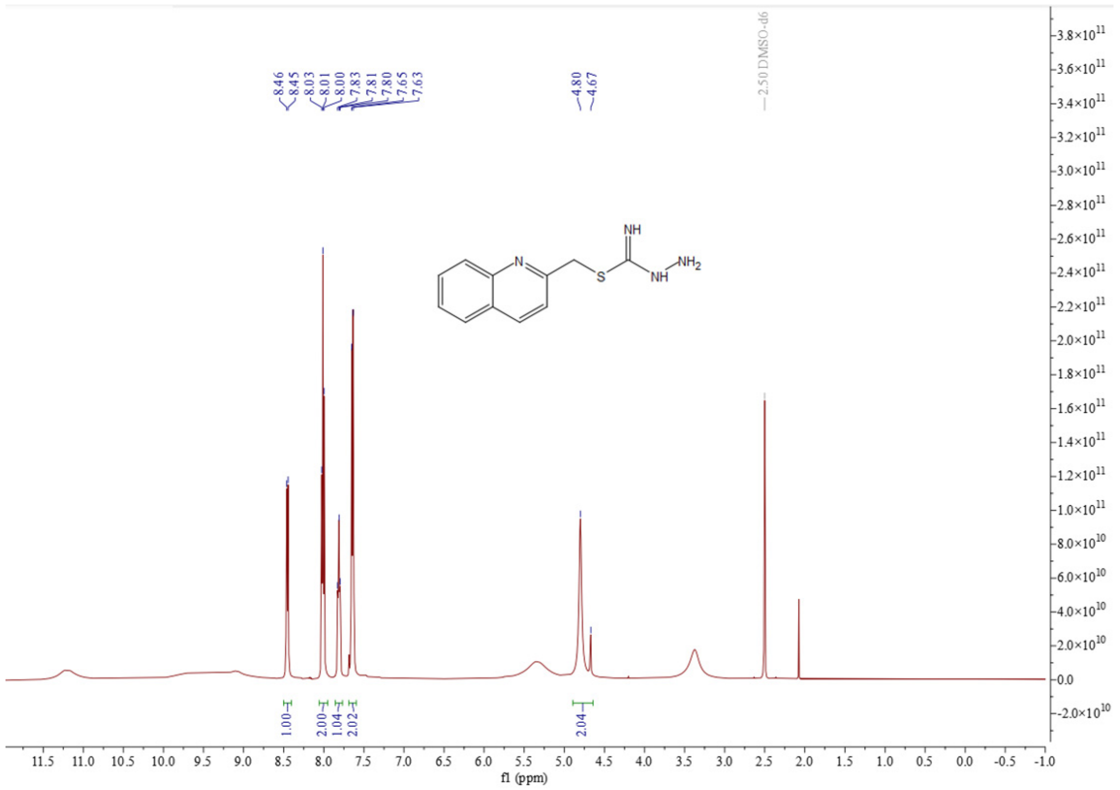

Figure S23 ^{1}H NMR spectrum of **12k**

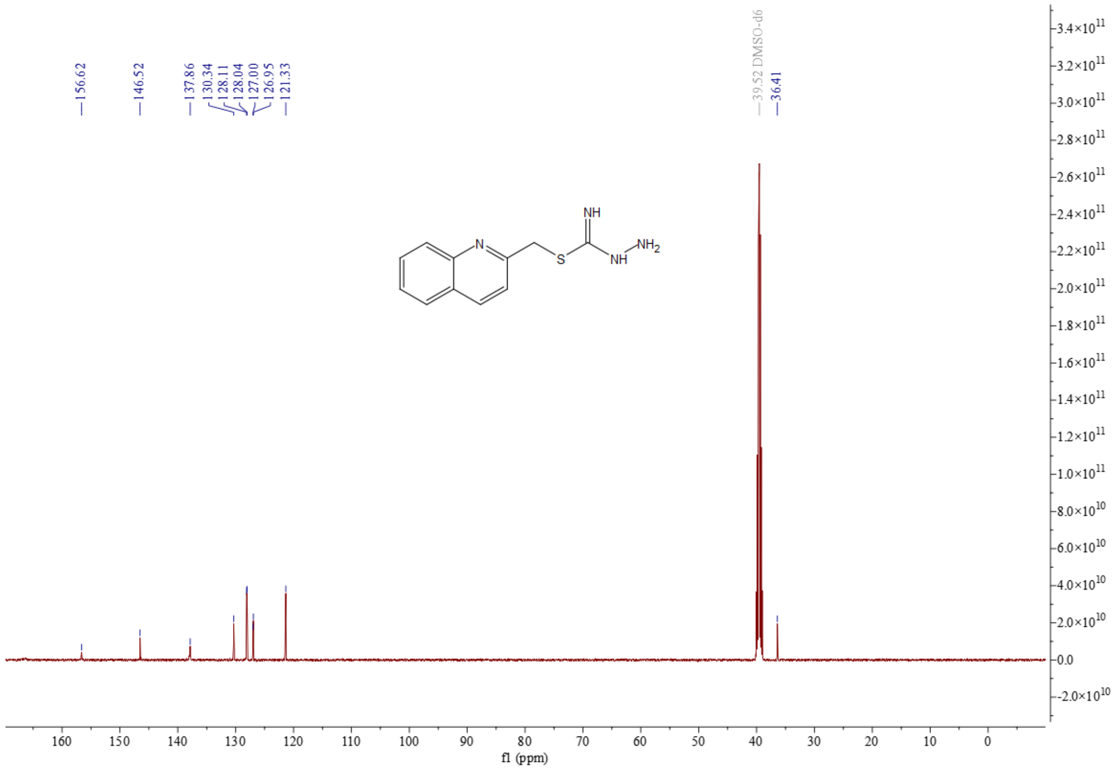

Figure S24 ^{13}C NMR spectrum of **12k**

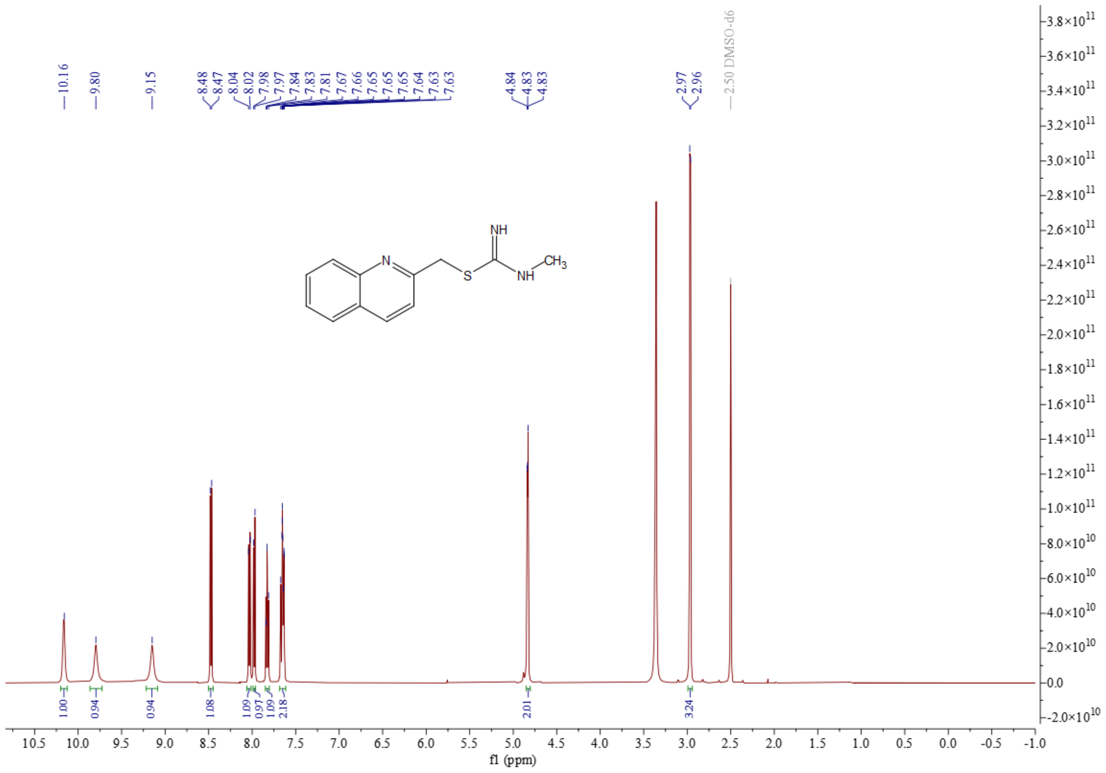

Figure S25 ^{1}H NMR spectrum of **12l**

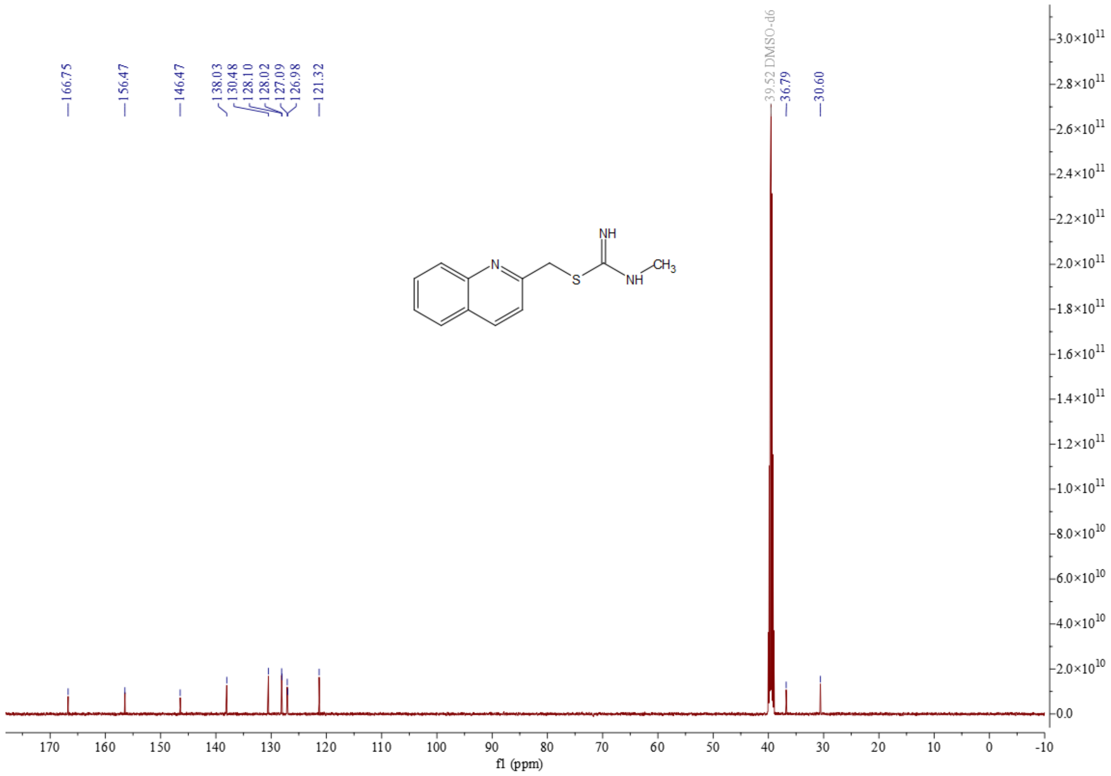

Figure S26 ^{13}C NMR spectrum of **12l**

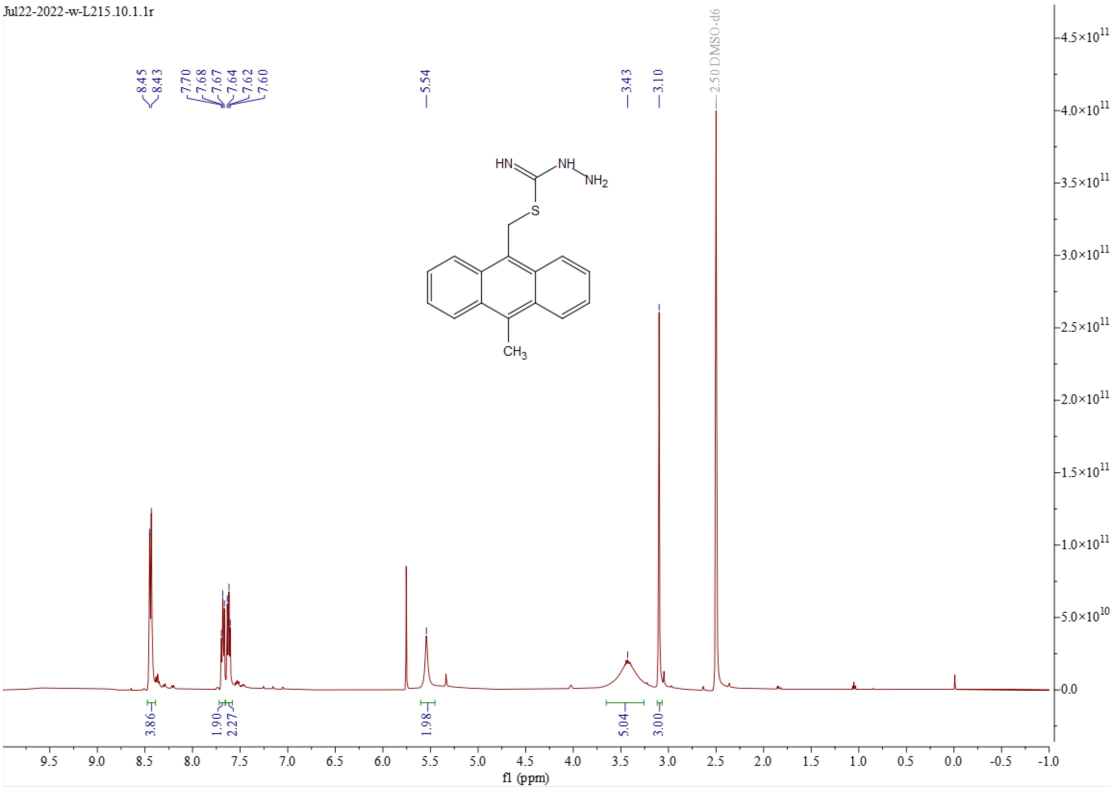

Figure S27 ^{1}H NMR spectrum of **13a**

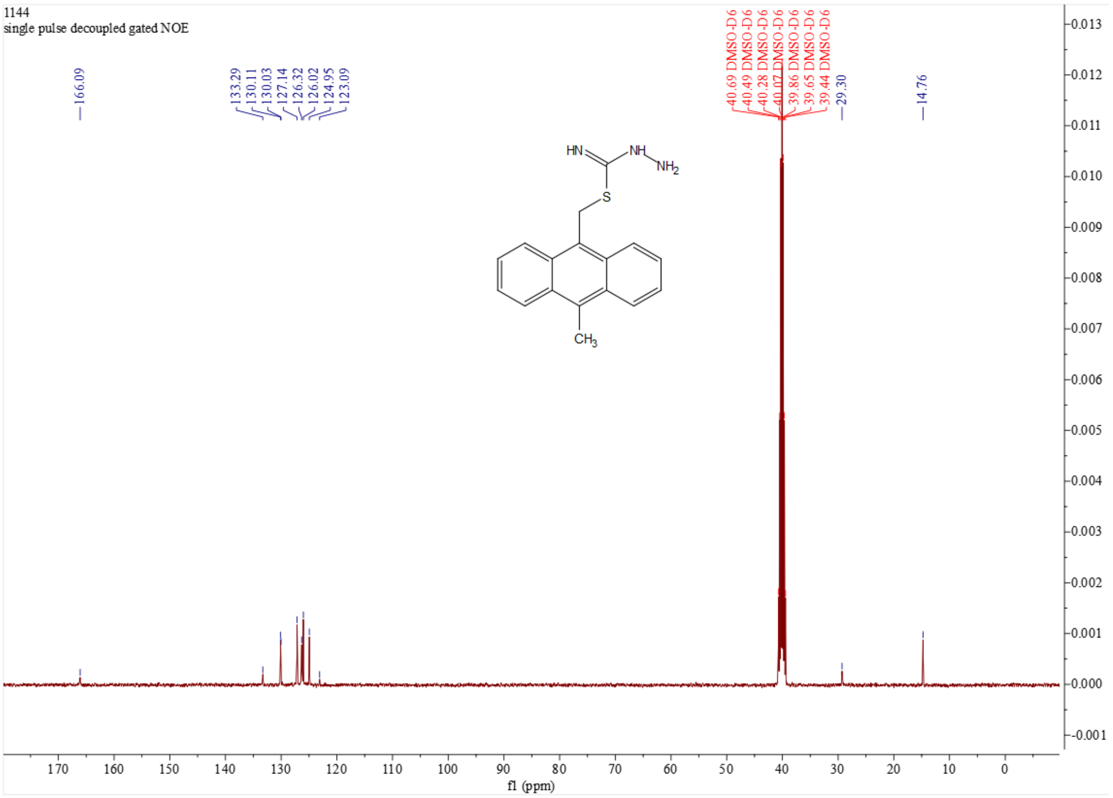

Figure S28 ^{13}C NMR spectrum of **13a**

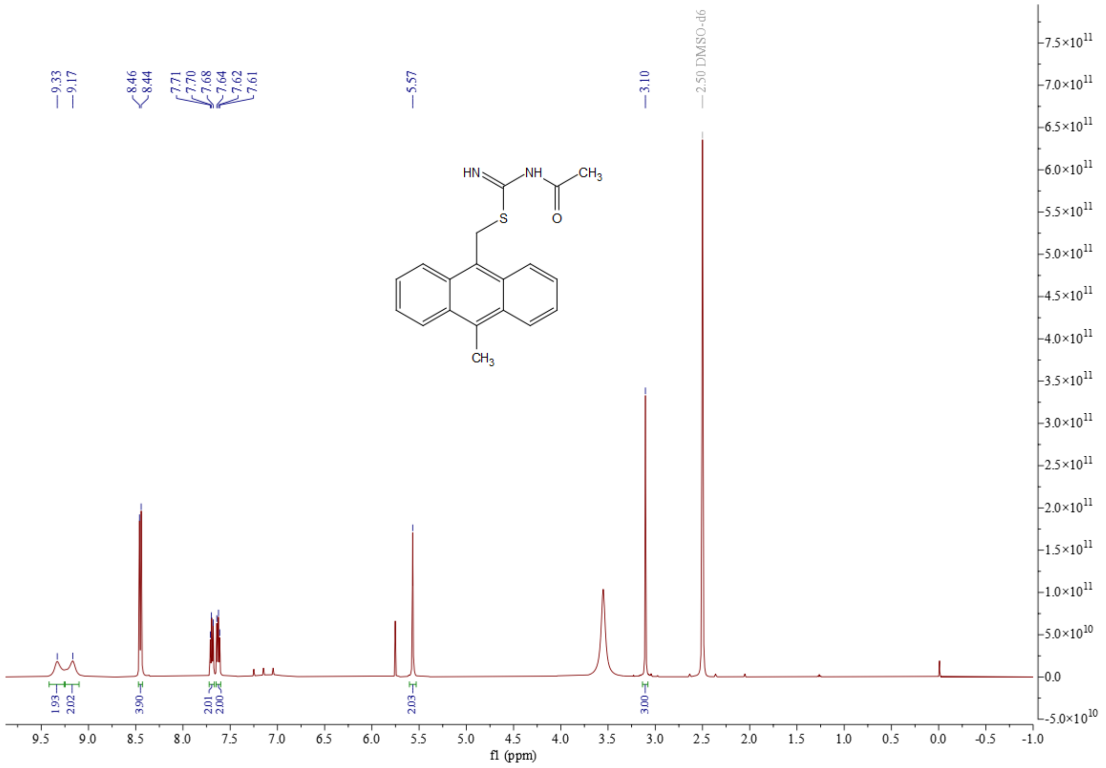

Figure S29 ^{1}H NMR spectrum of **13b**

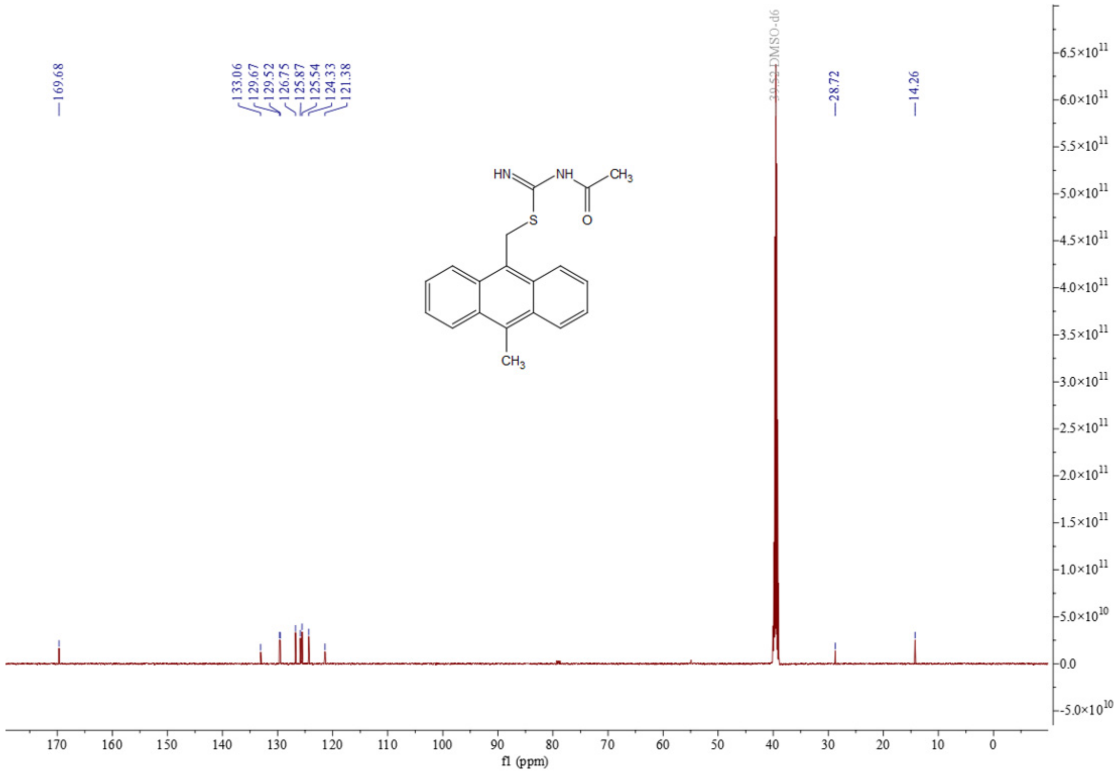

Figure S30 ^{13}C NMR spectrum of **13b**

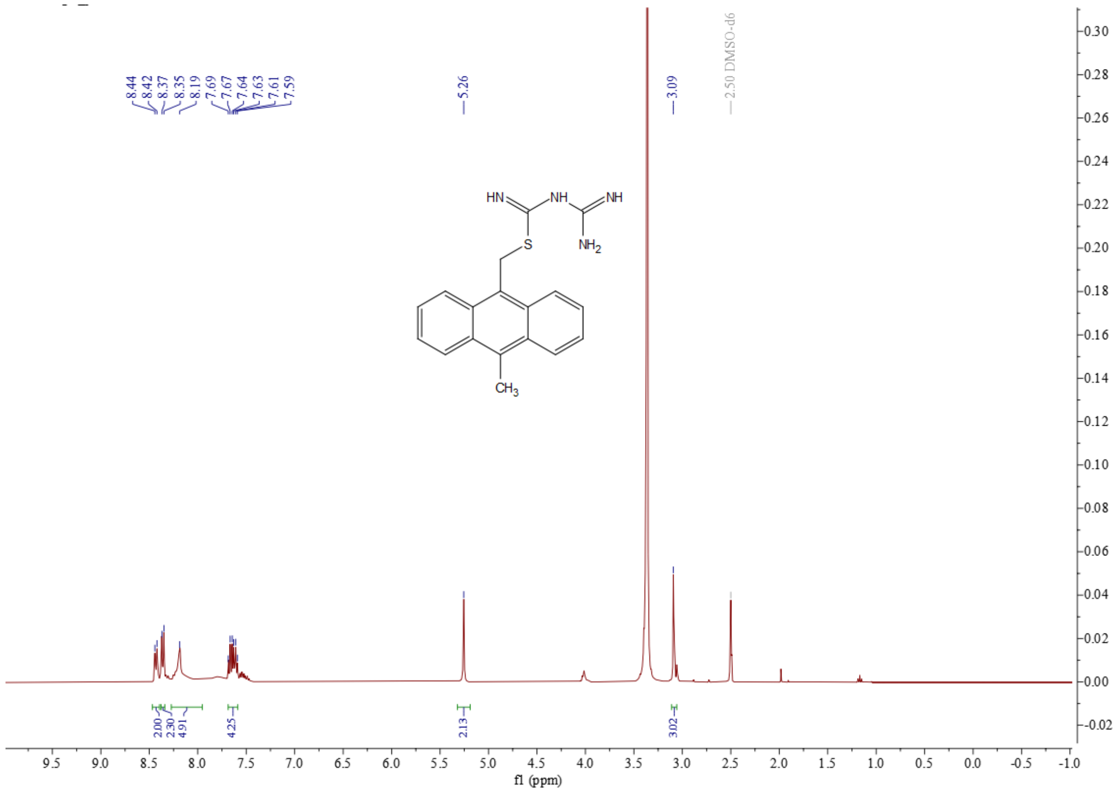

Figure S31 ^{1}H NMR spectrum of **13c**

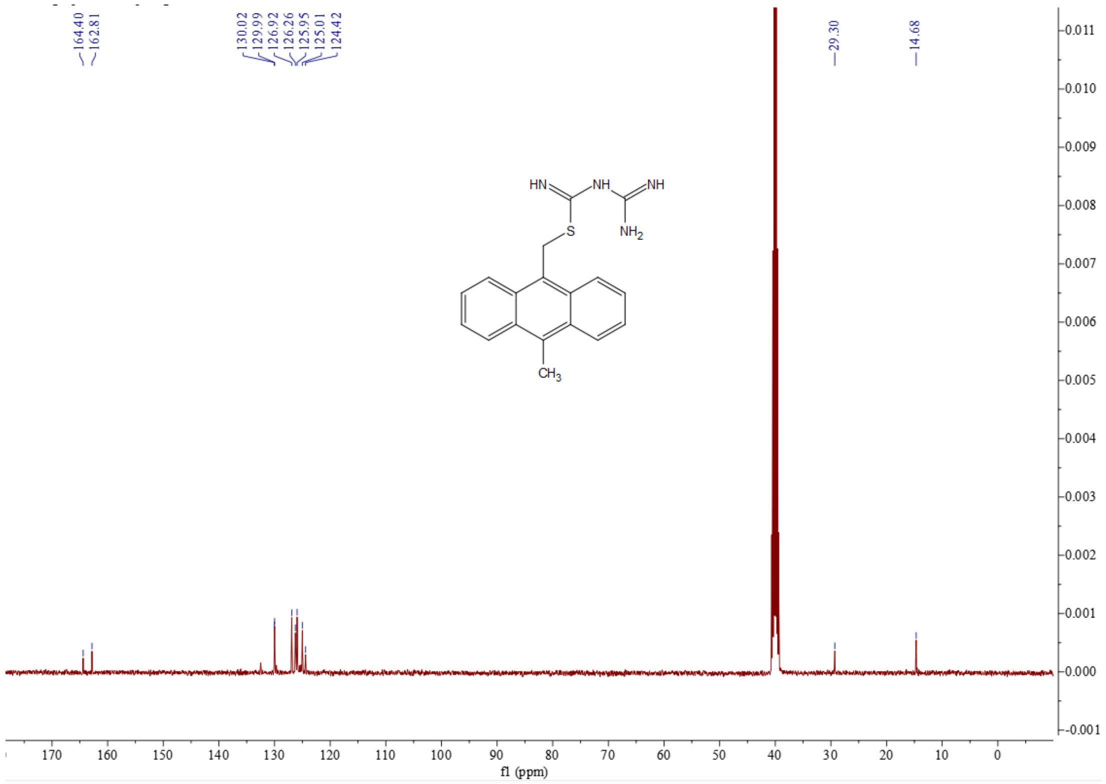

Figure S32 ^{13}C NMR spectrum of **13c**

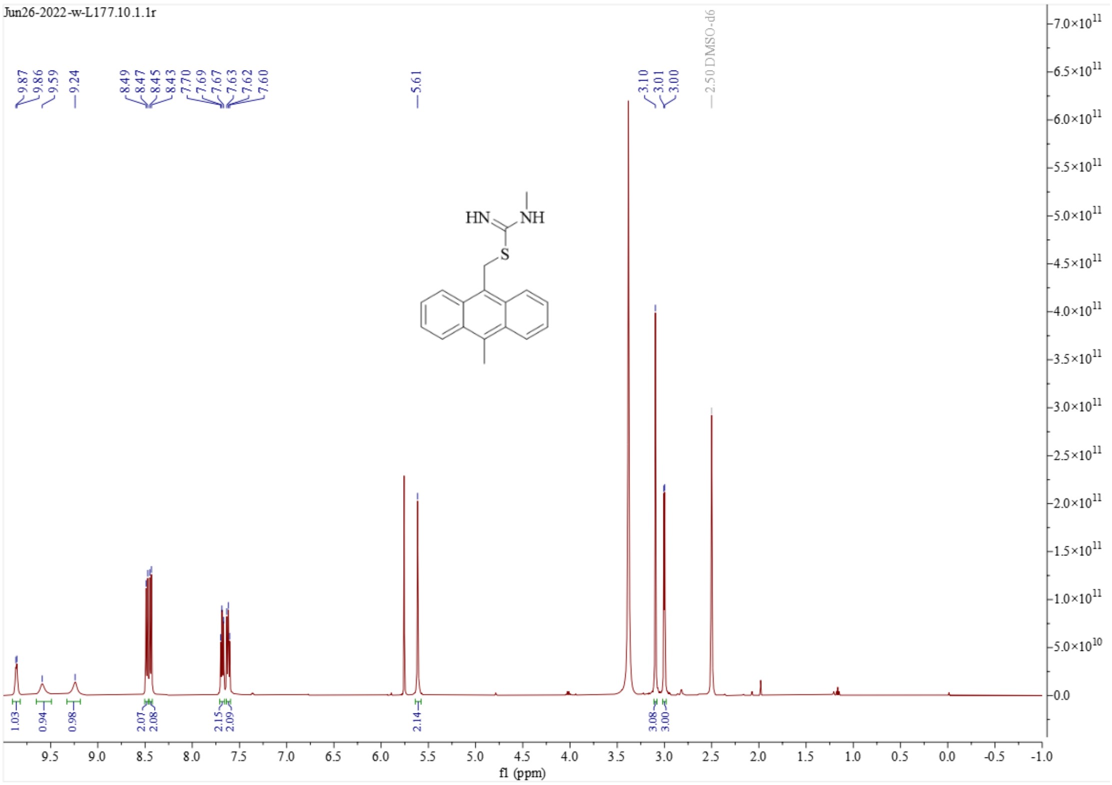

Figure S33 ^{1}H NMR spectrum of **13d**

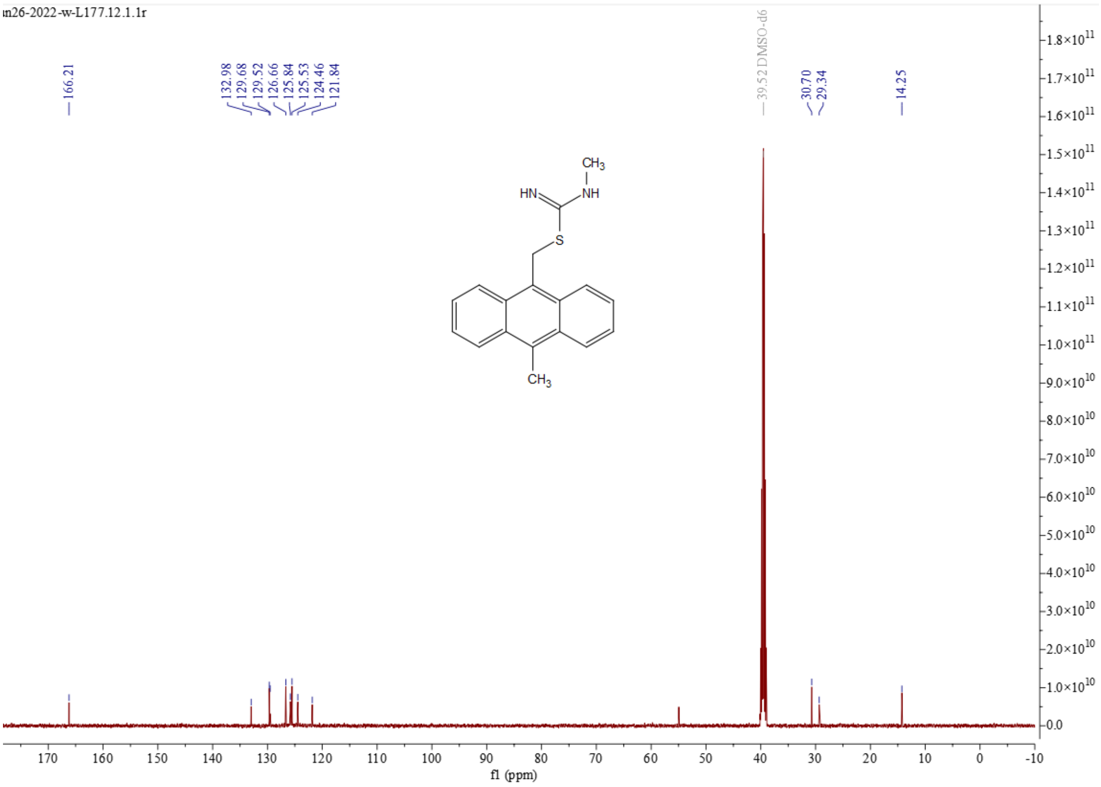

Figure S34 ^{13}C NMR spectrum of **13d**

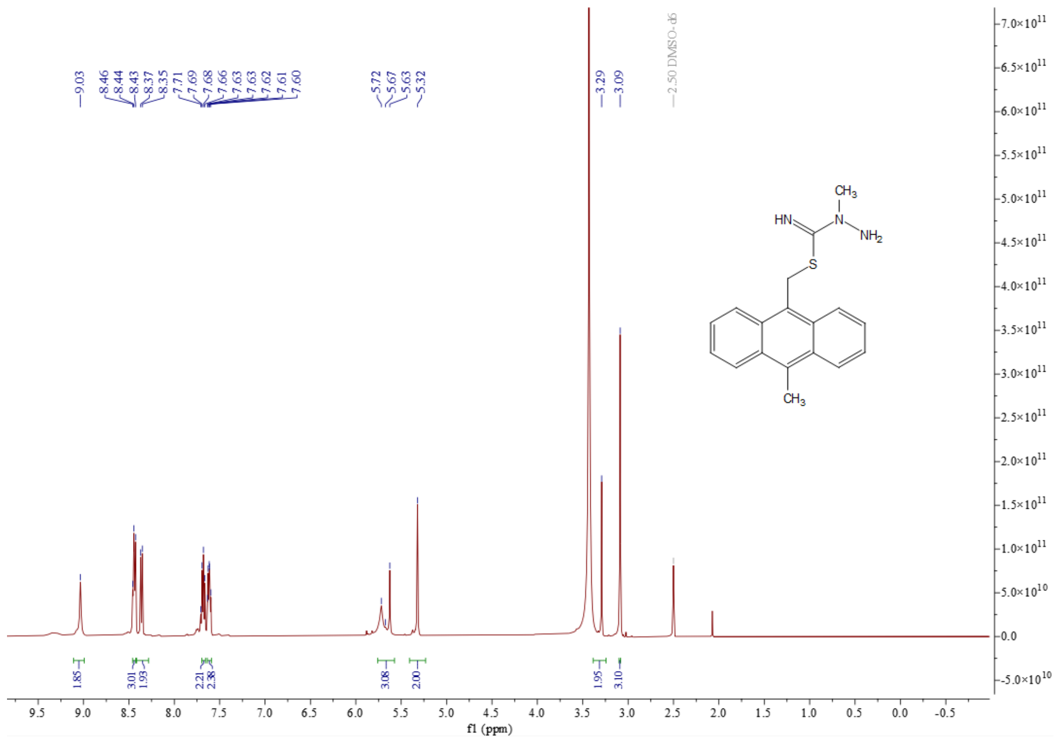

Figure S35 ^{1}H NMR spectrum of **13e**

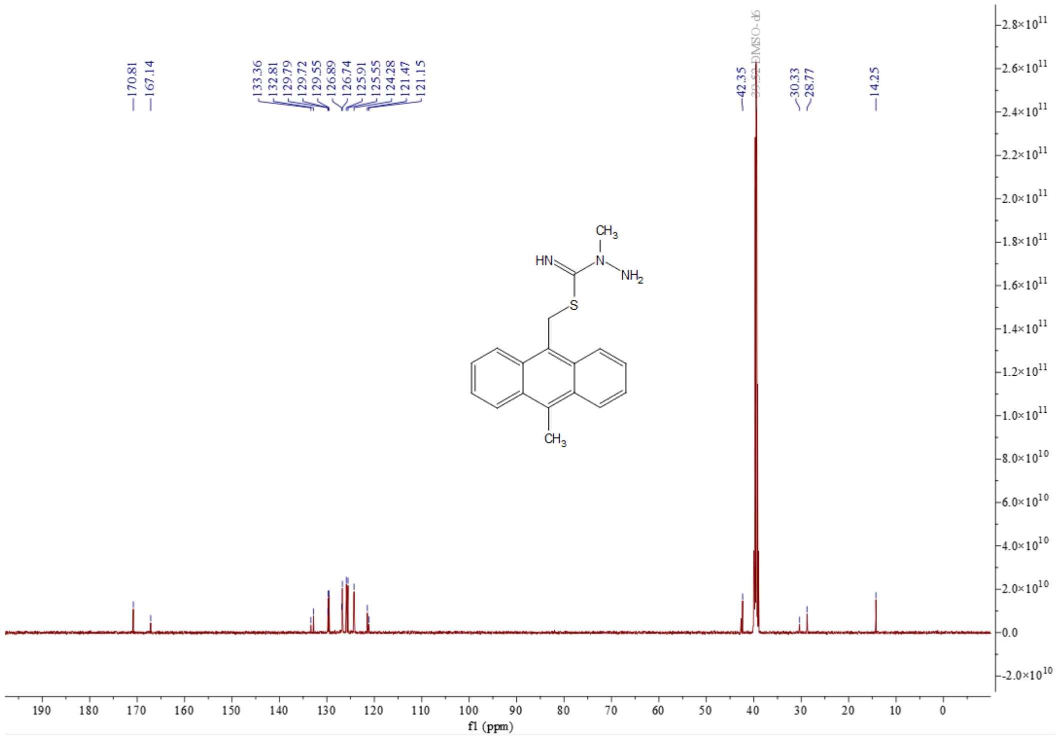

Figure S36 ^{13}C NMR spectrum of **13e**

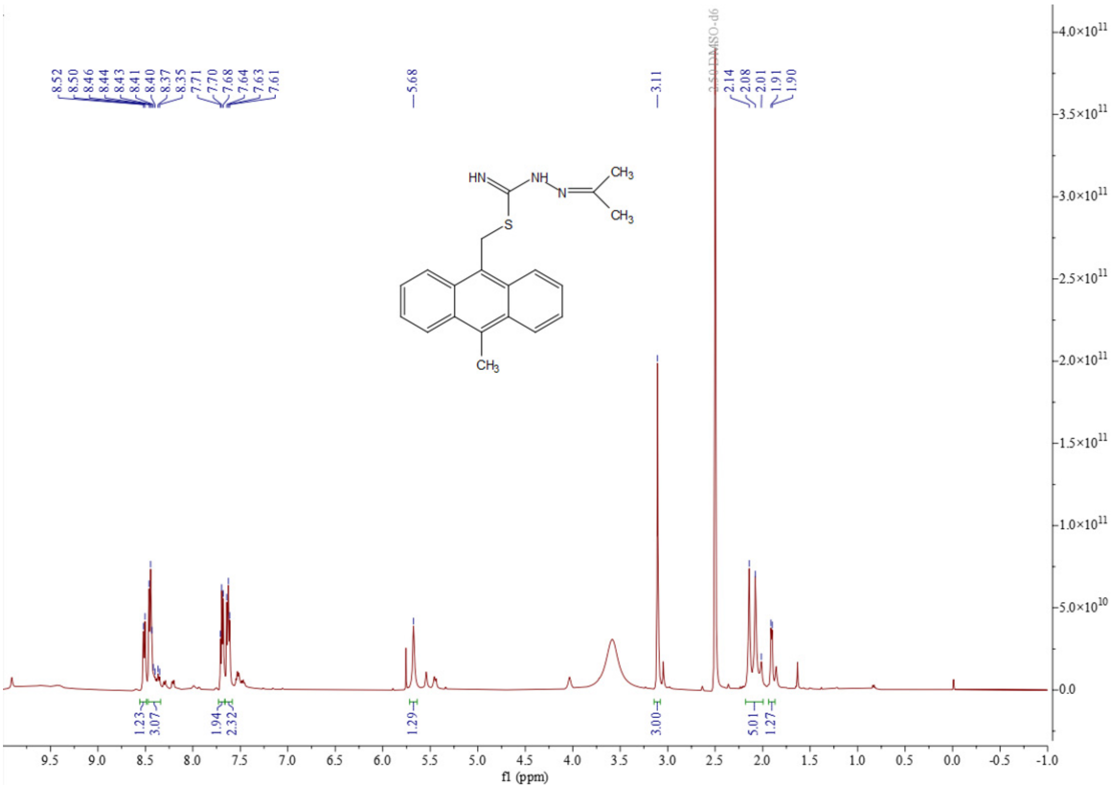

Figure S37 ^{1}H NMR spectrum of **13f**

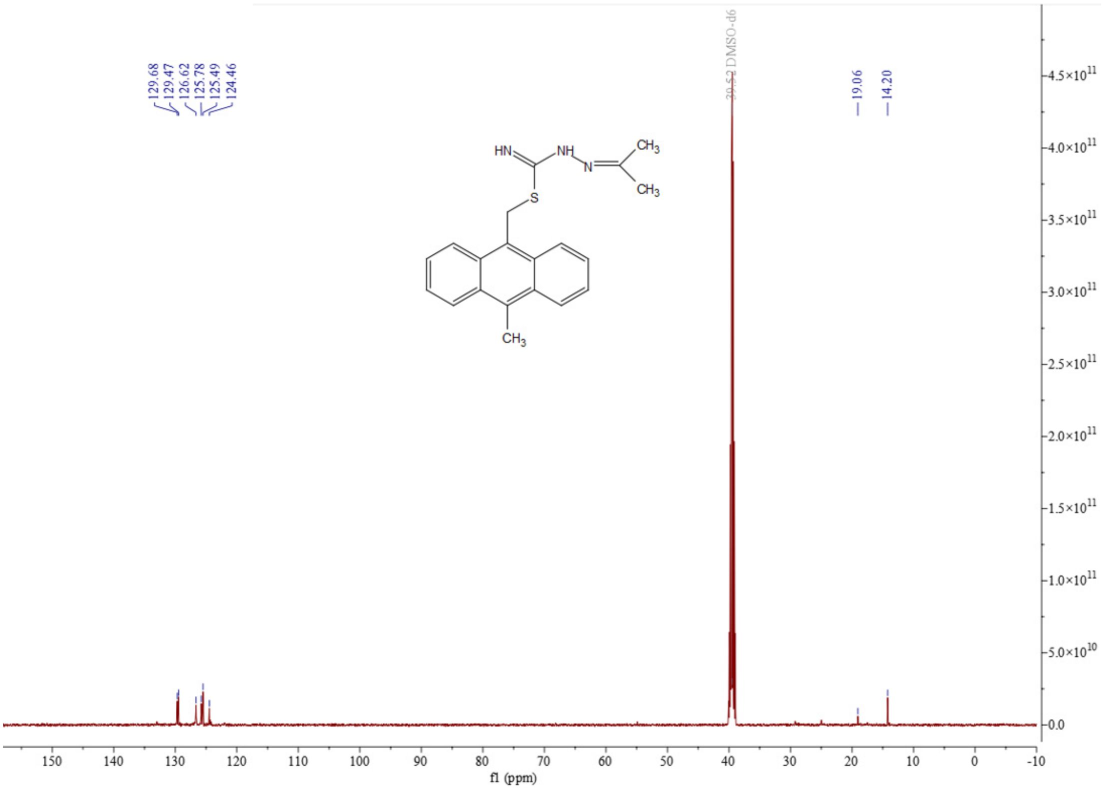

Figure S38 ^{13}C NMR spectrum of **13f**

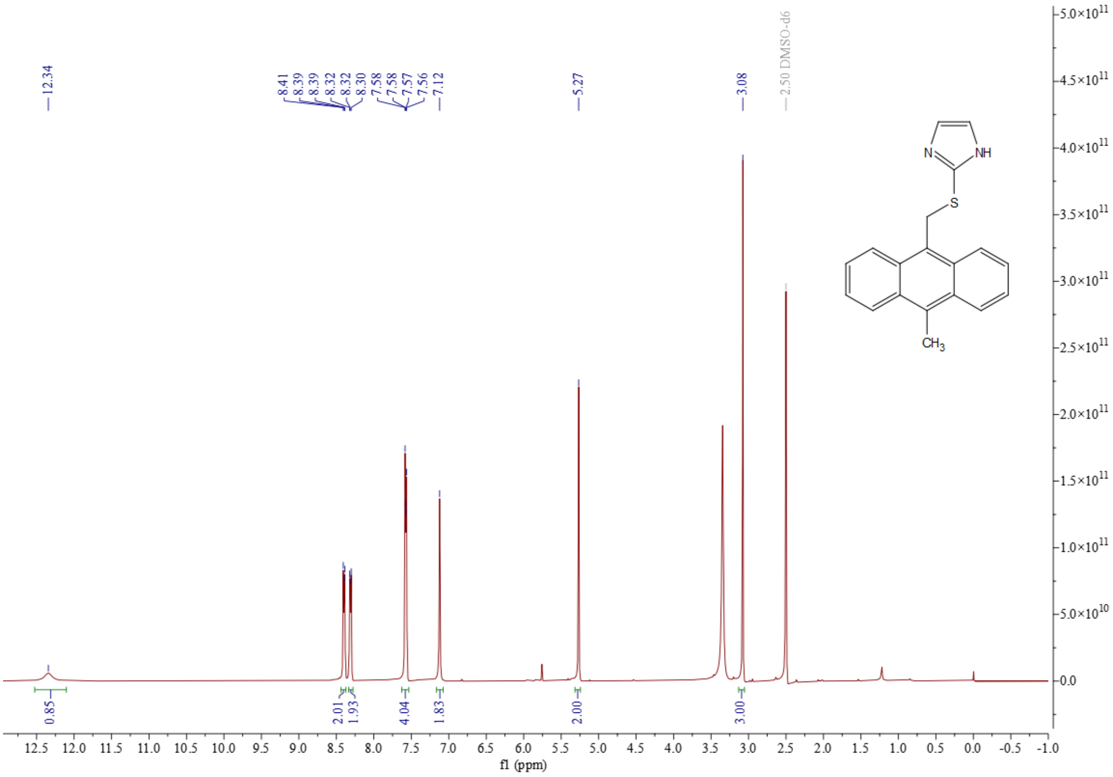

Figure S39 ^{1}H NMR spectrum of **13g**

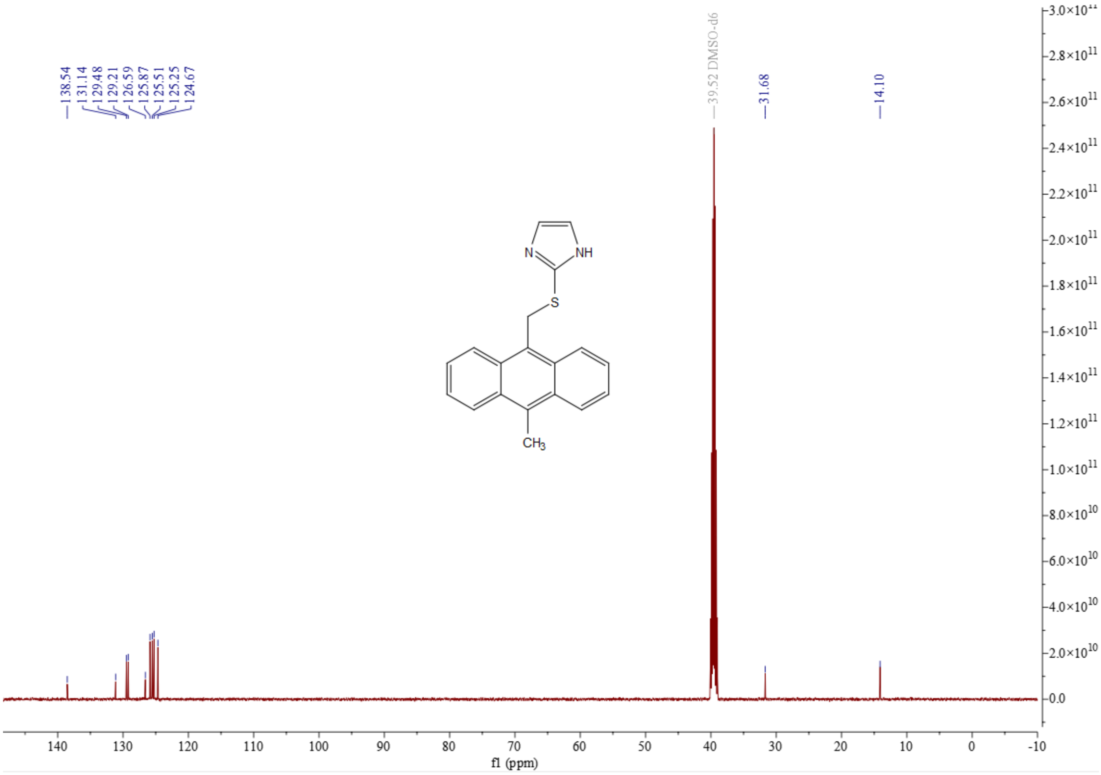

Figure S40 ^{13}C NMR spectrum of **13g**

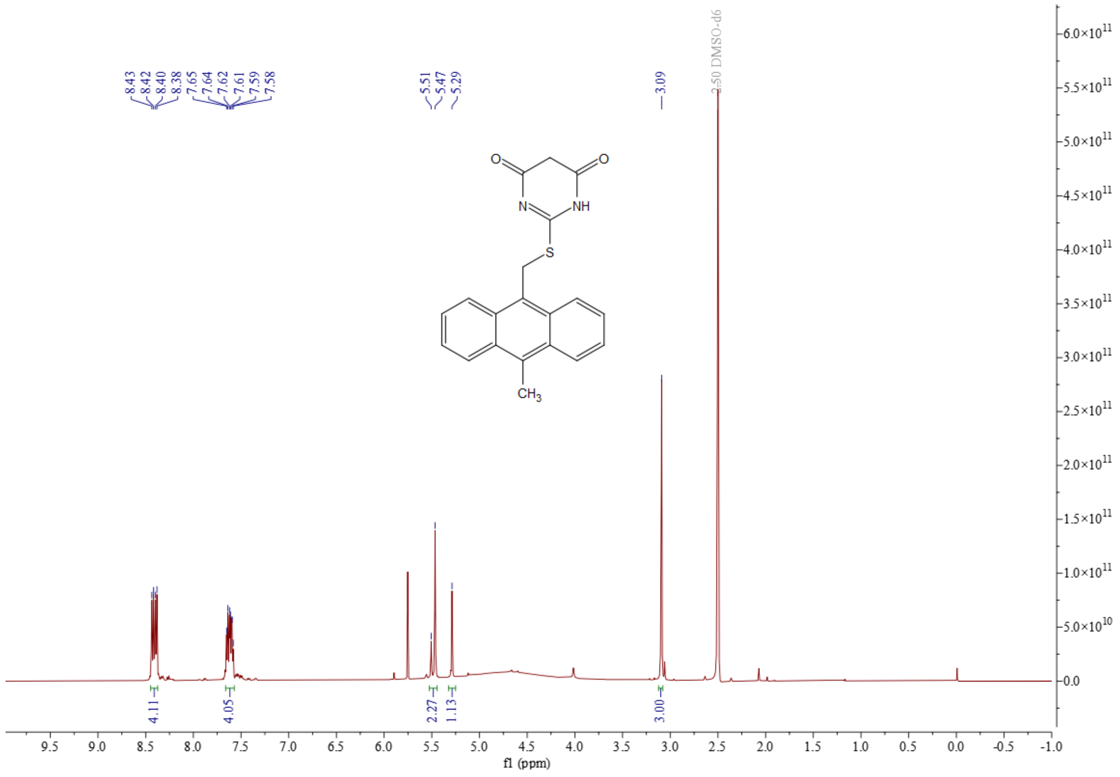

Figure S41 ^{1}H NMR spectrum of **13h**

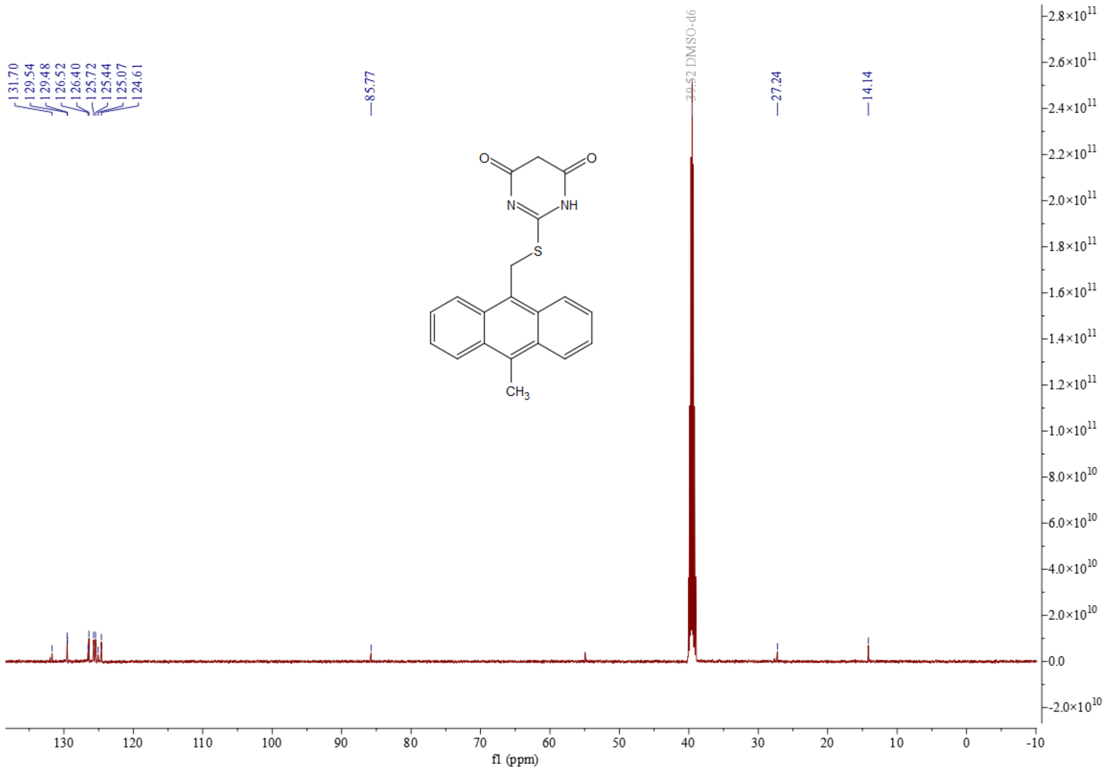

Figure S42 ^{13}C NMR spectrum of **13h**

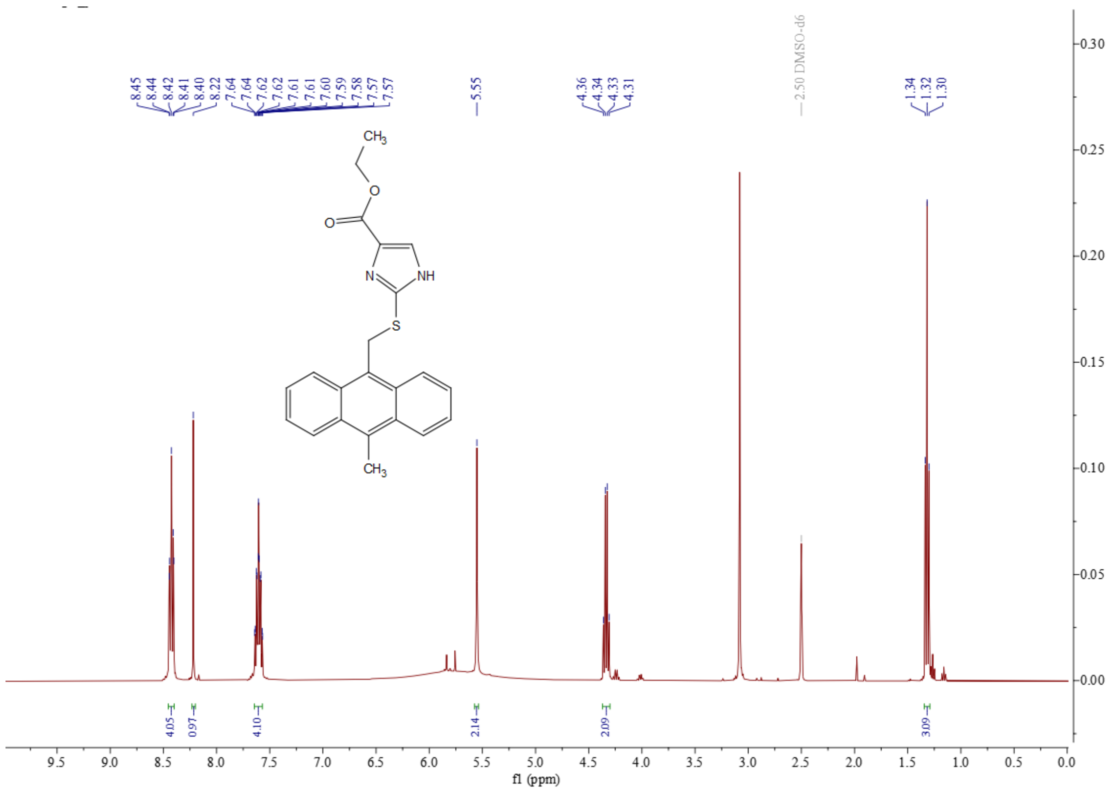

Figure S43 ^{1}H NMR spectrum of **13i**

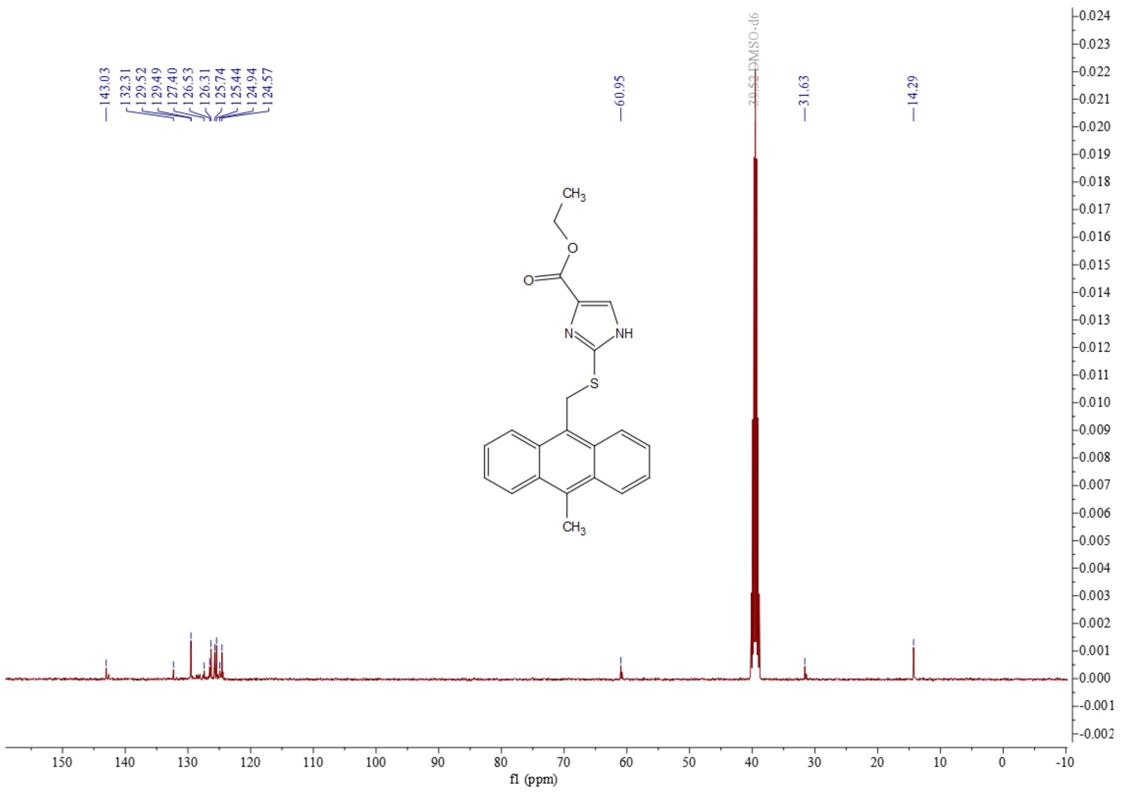

Figure S44 ^{13}C NMR spectrum of **13i**

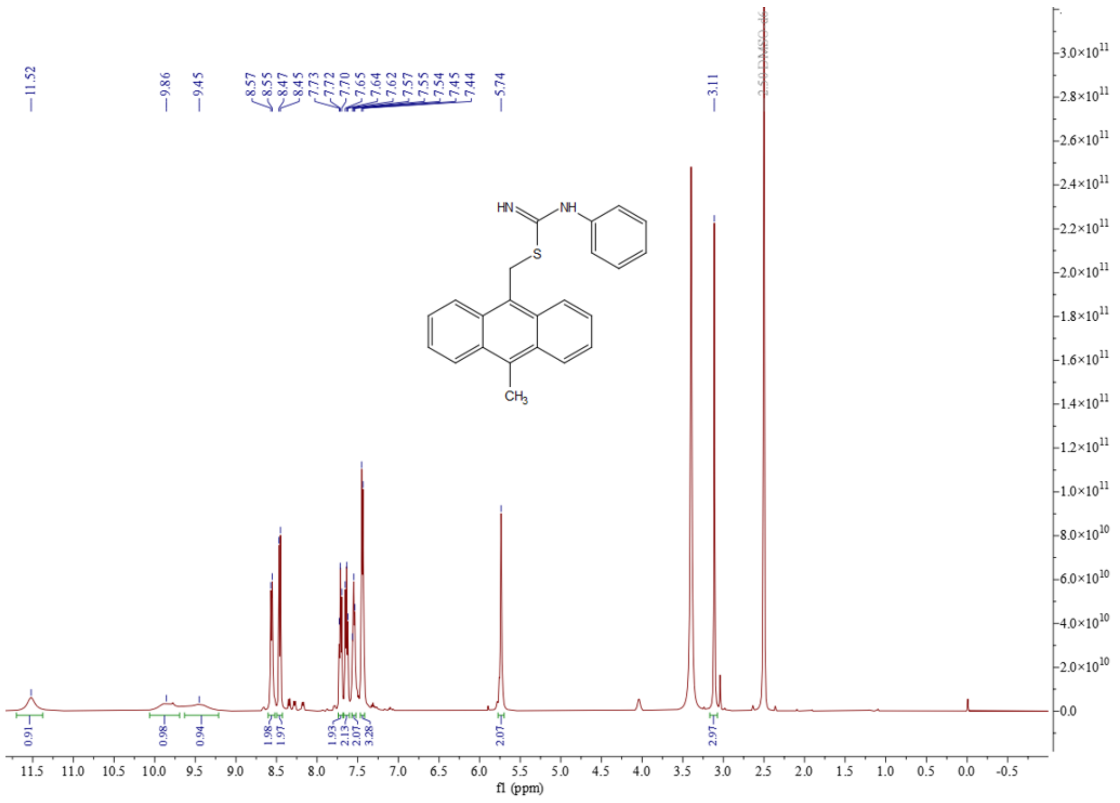

Figure S45 ^{1}H NMR spectrum of **13j**

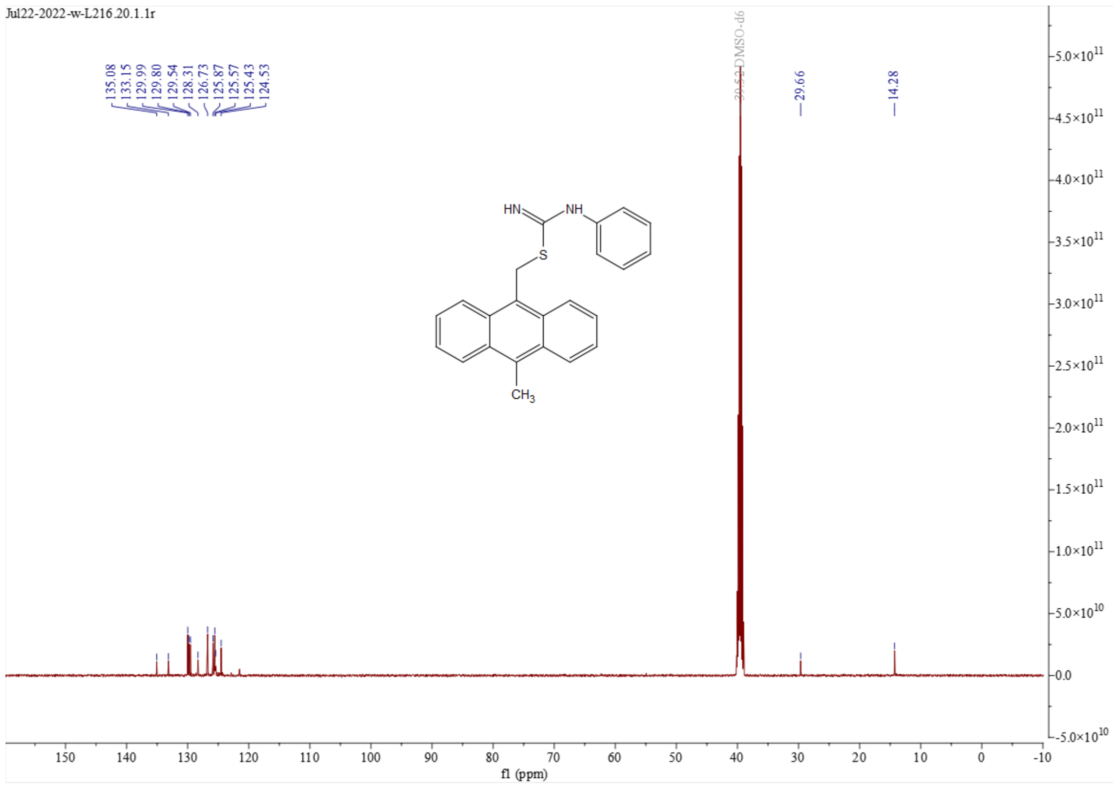

Figure S46 ^{13}C NMR spectrum of **13j**

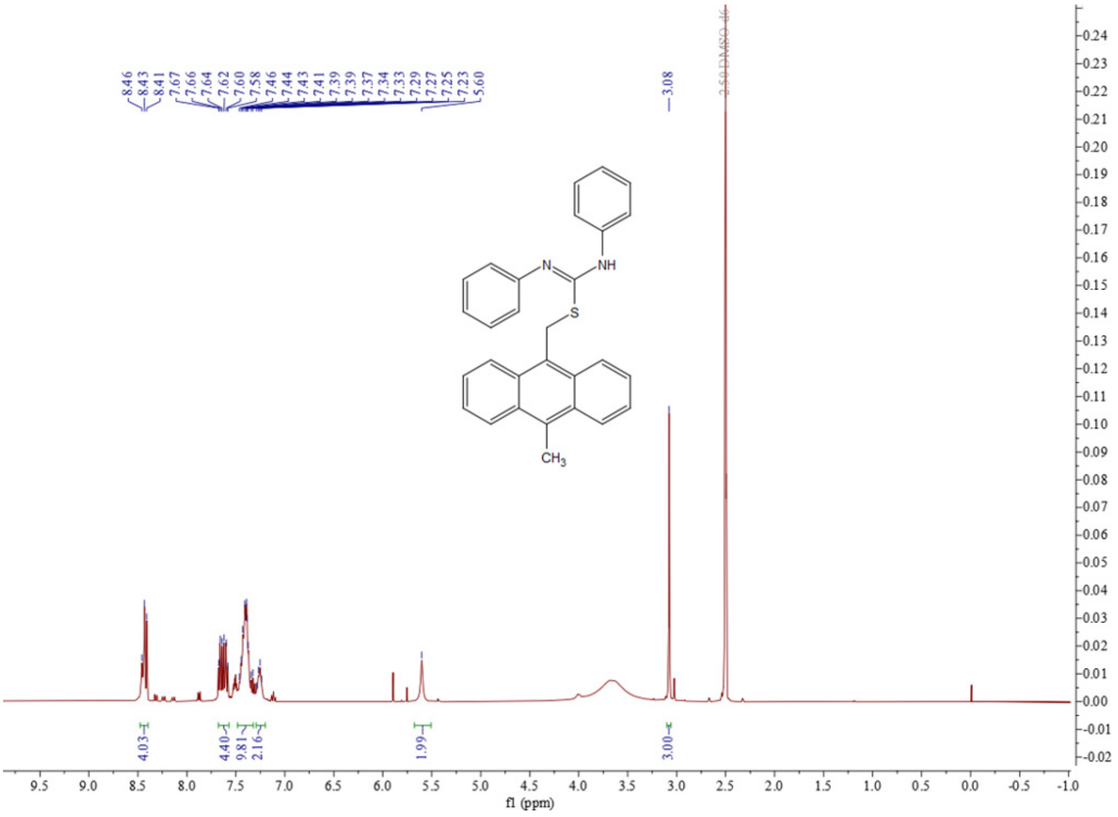

Figure S47 ^{1}H NMR spectrum of **13k**

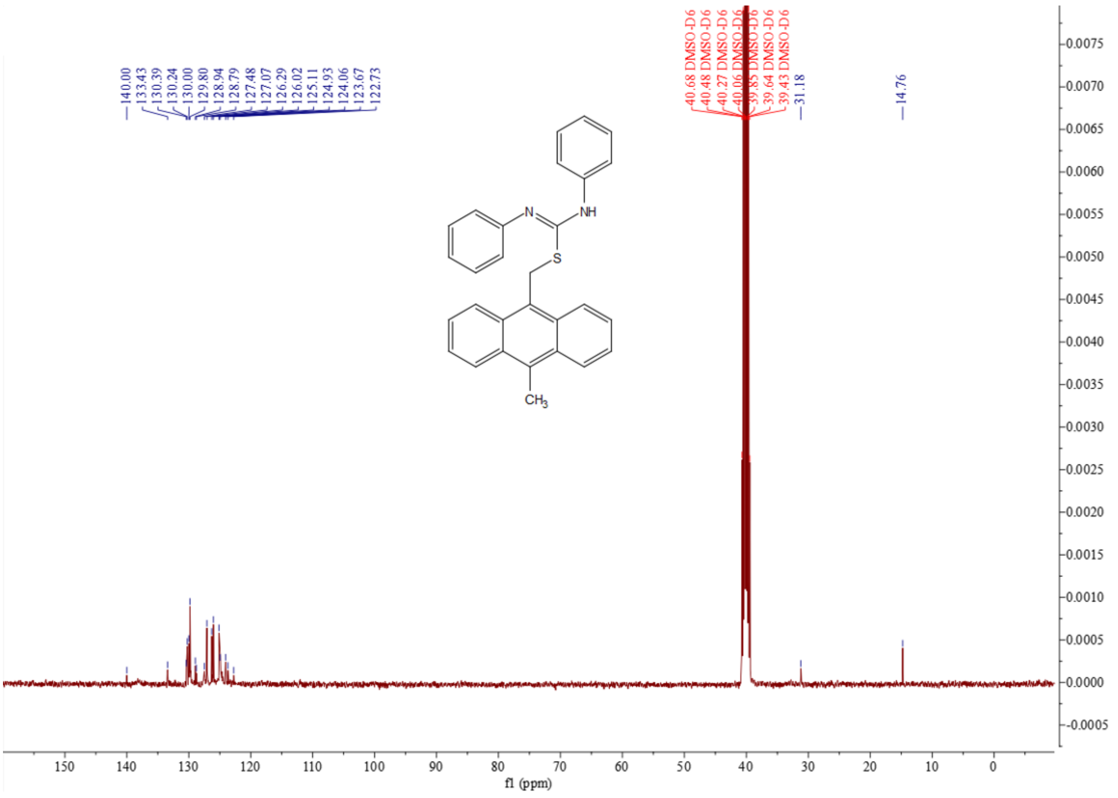

Figure S48 ^{13}C NMR spectrum of **13k**

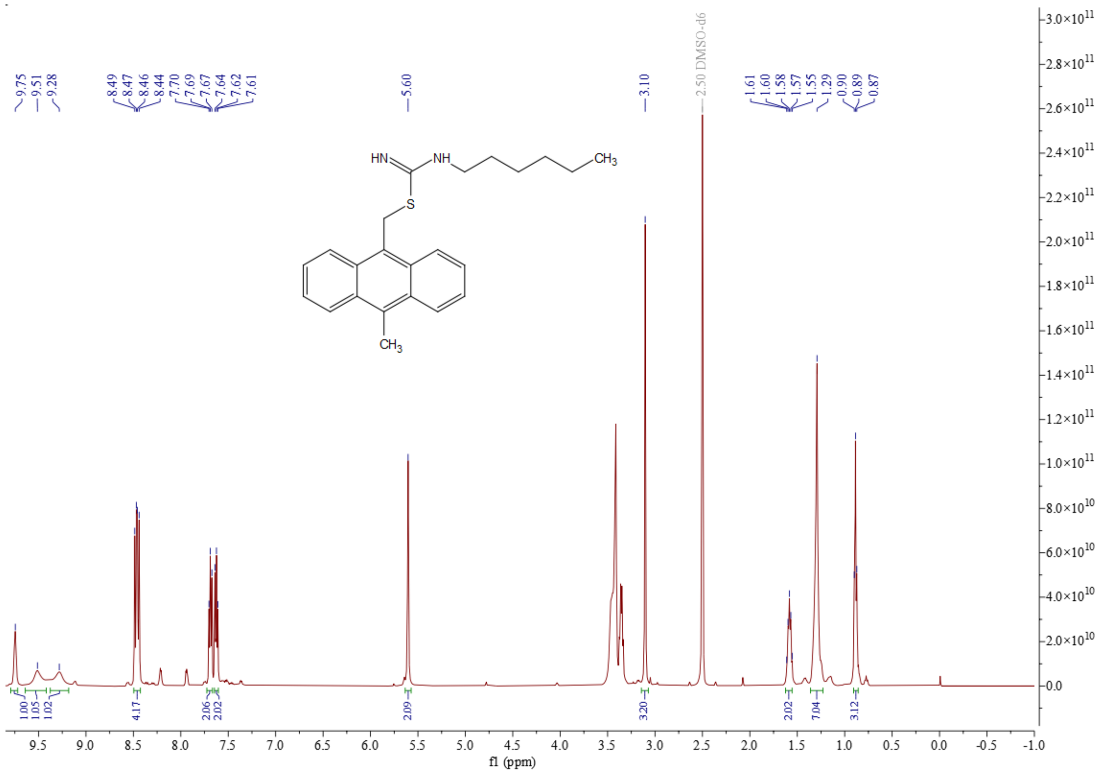

Figure S49 ^{1}H NMR spectrum of **13l**

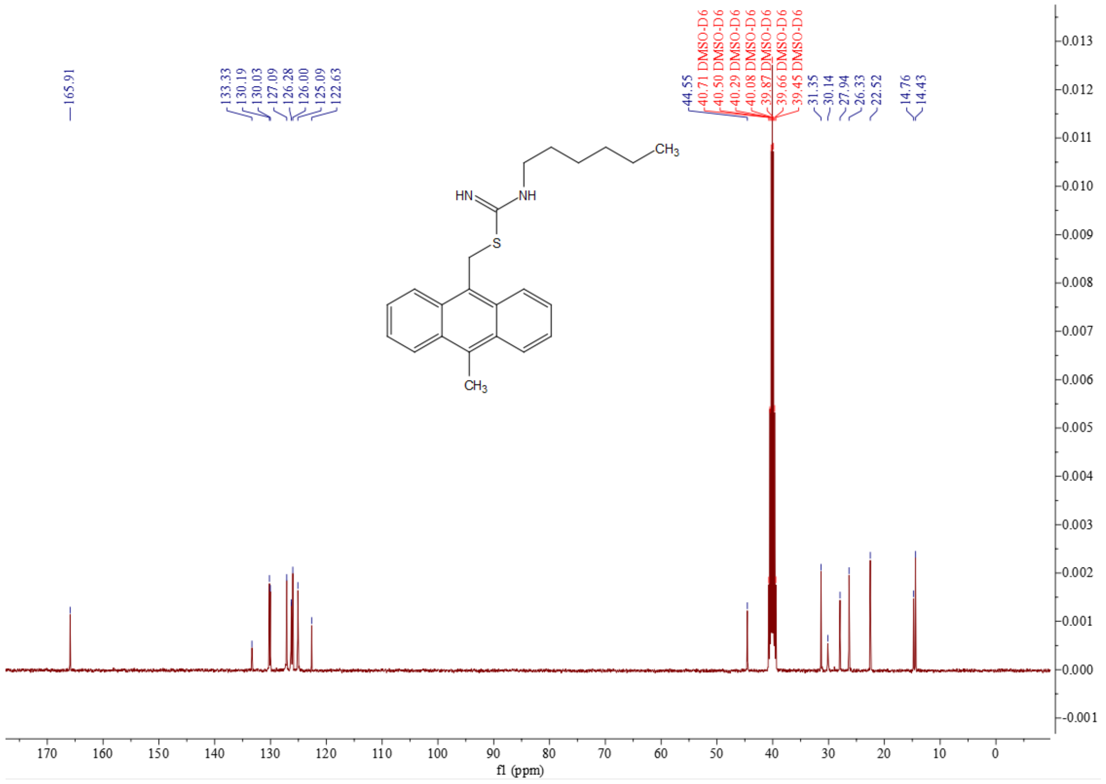

Figure S50 ^{13}C NMR spectrum of **13l**

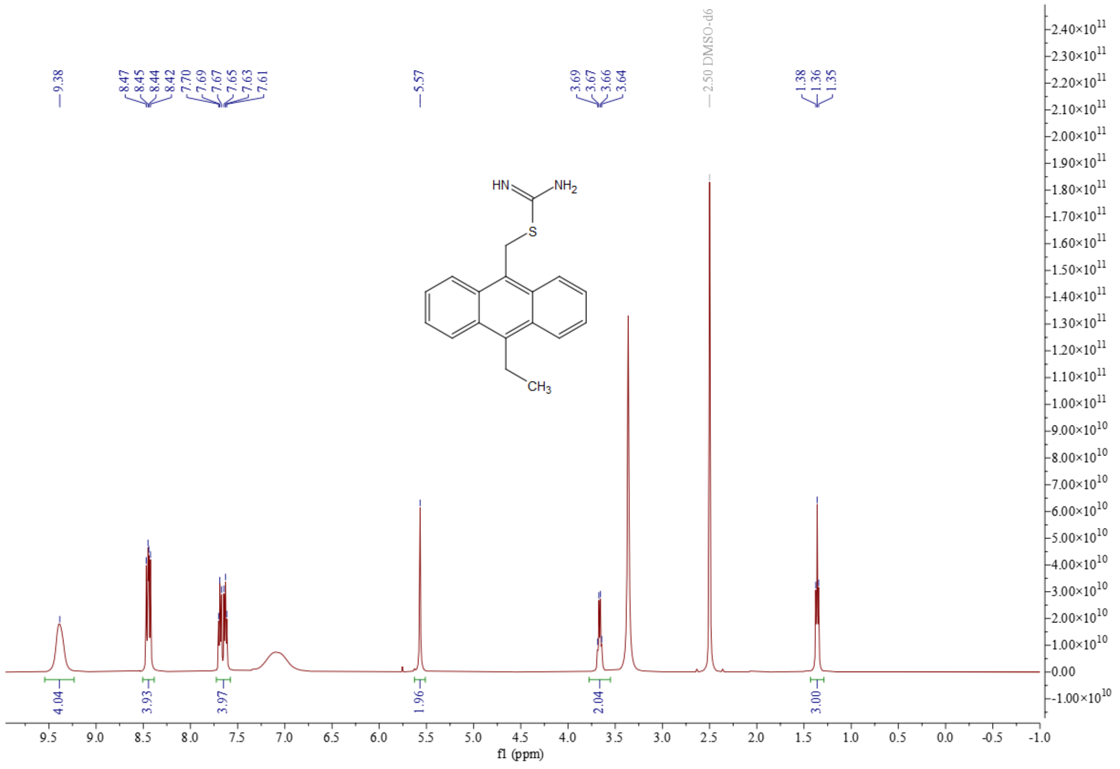

Figure S51 ^{1}H NMR spectrum of **14a**

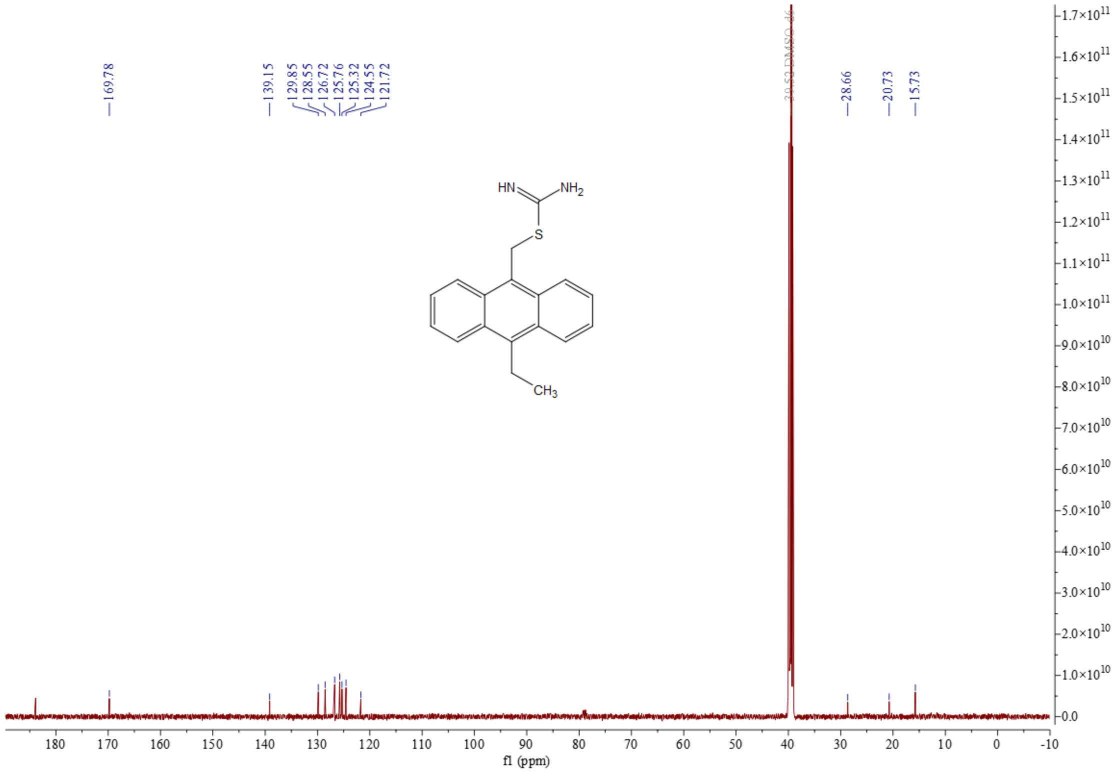

Figure S52 ^{13}C NMR spectrum of **14a**

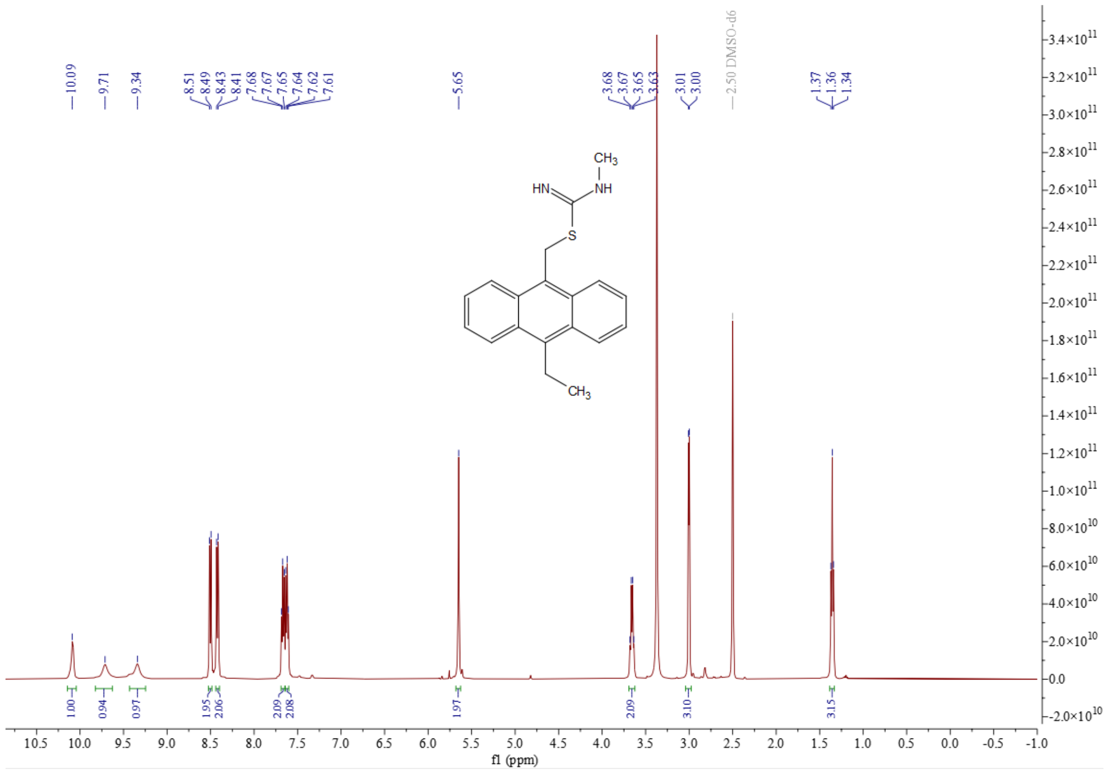

Figure S53 ^{1}H NMR spectrum of **14b**

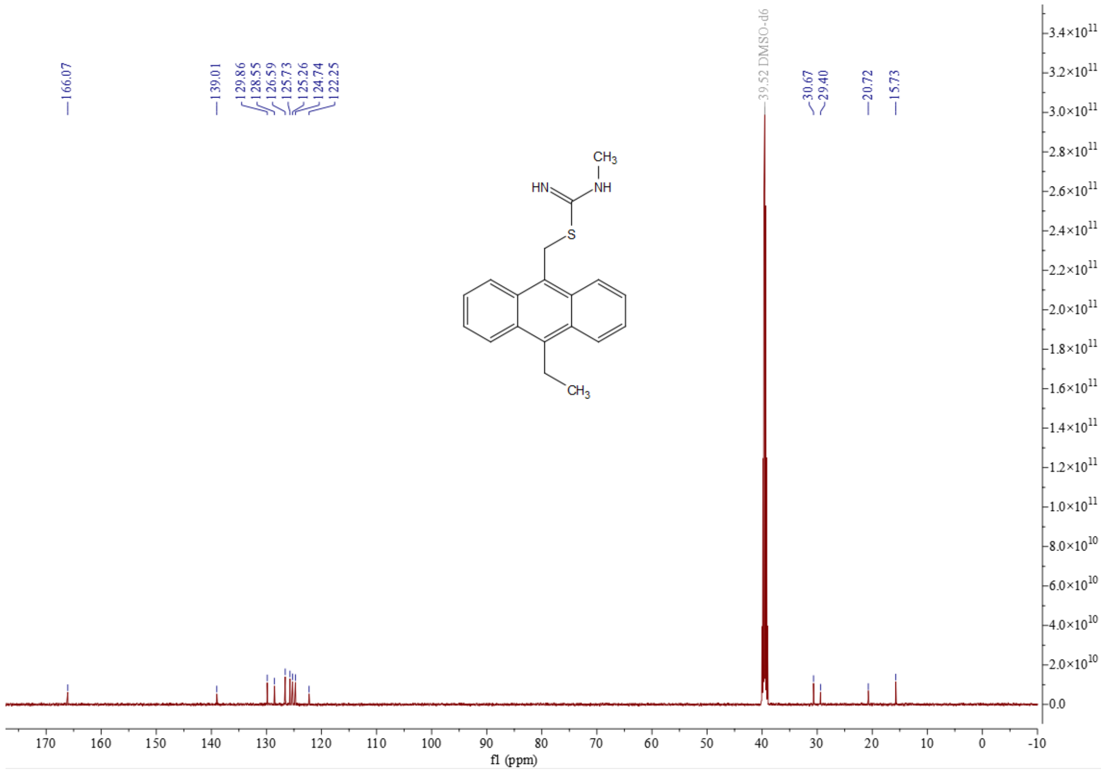

Figure S54 ^{13}C NMR spectrum of **14b**

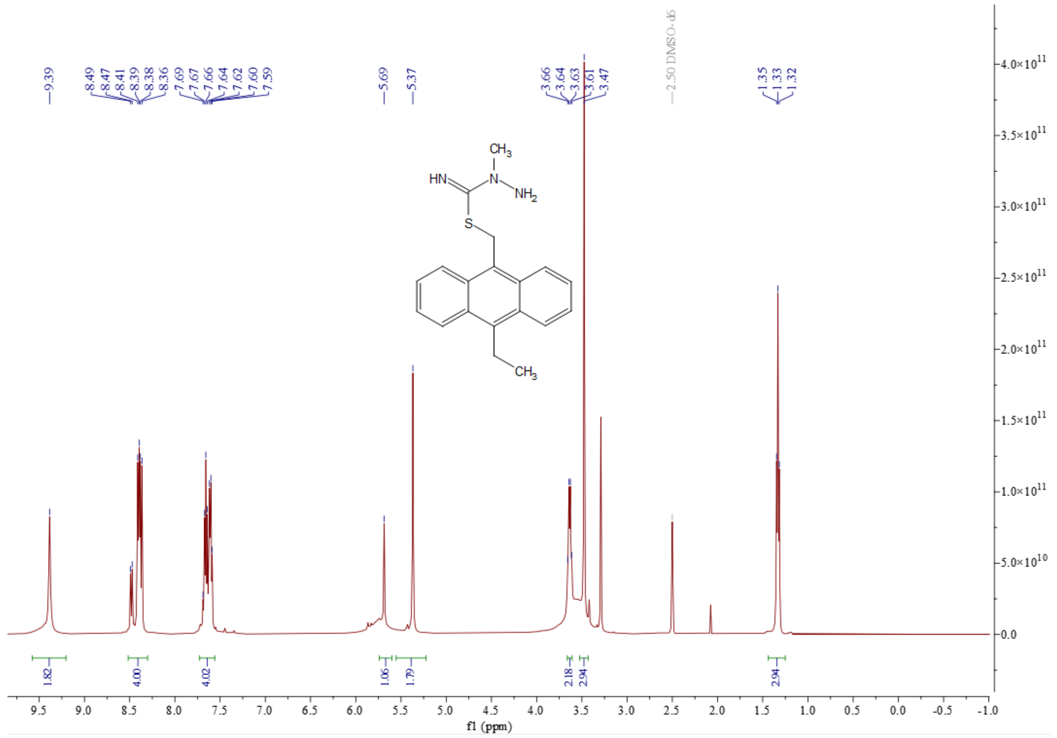

Figure S55 ^{1}H NMR spectrum of **14c**

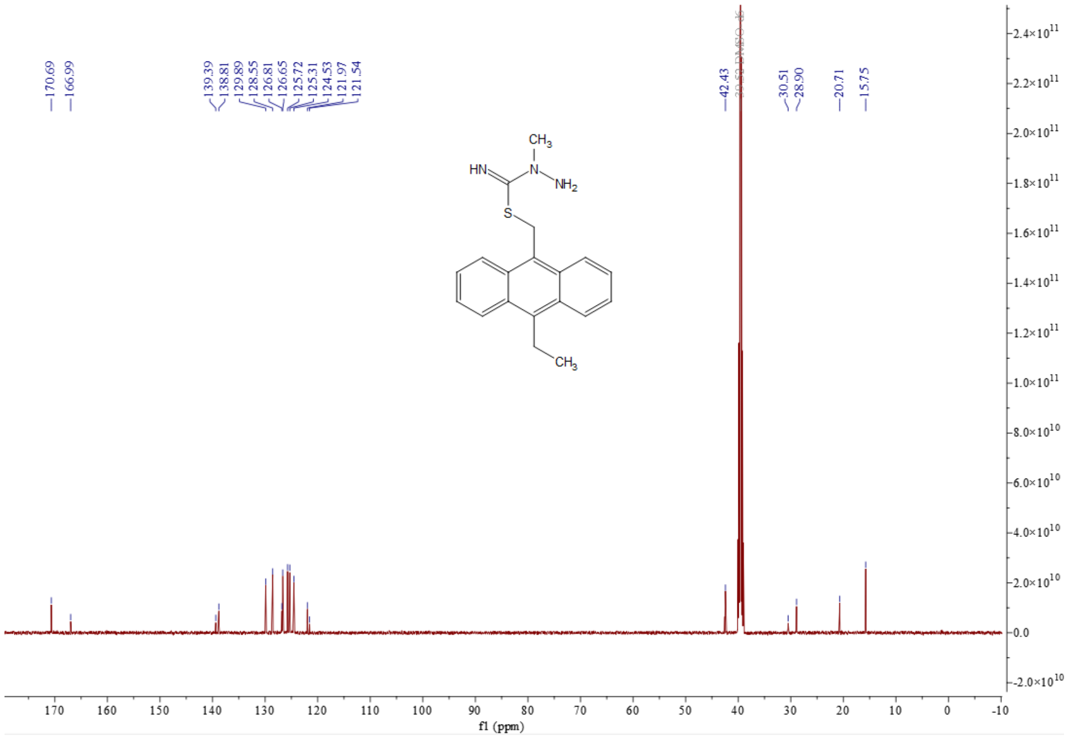

Figure S56 ^{13}C NMR spectrum of **14c**

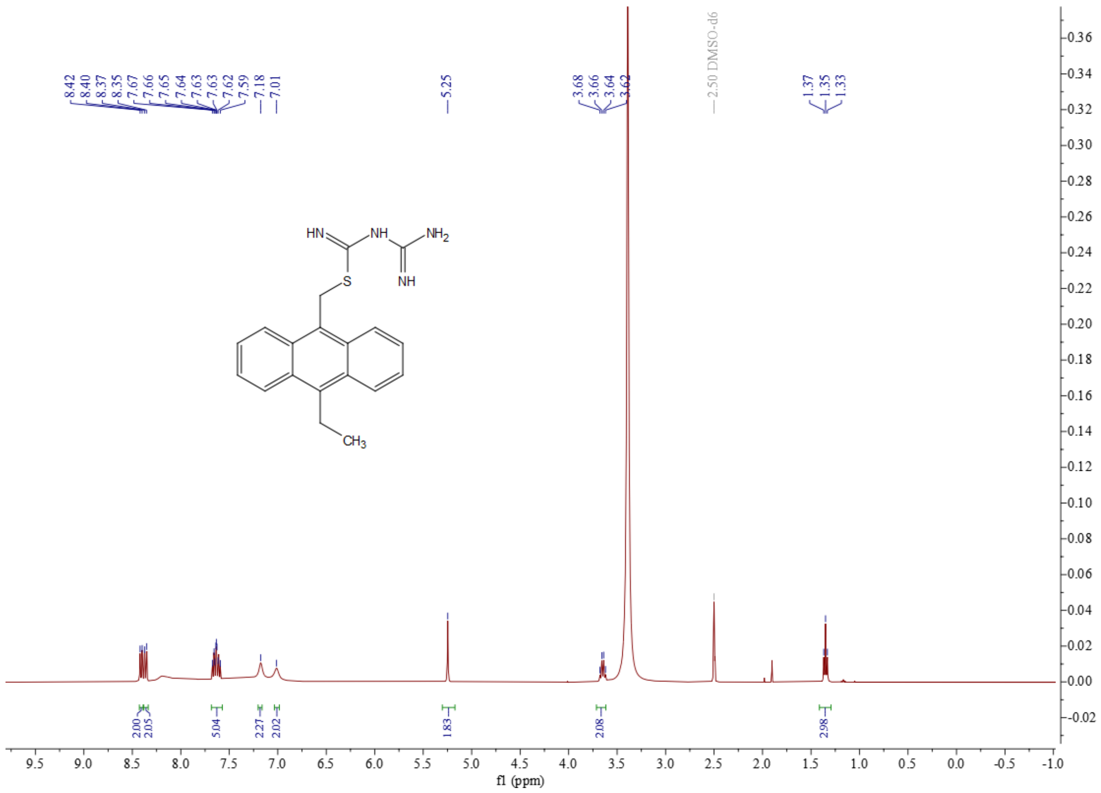

Figure S57 ^{1}H NMR spectrum of **14d**

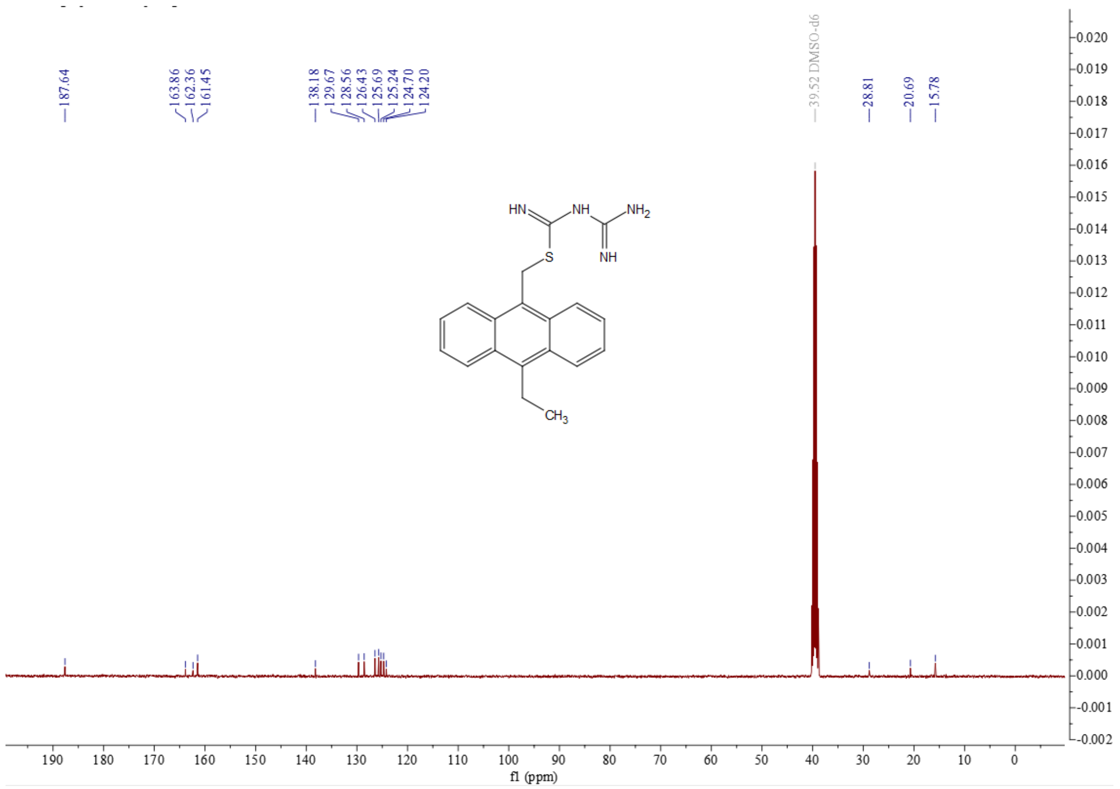

Figure S58 ^{13}C NMR spectrum of **14d**

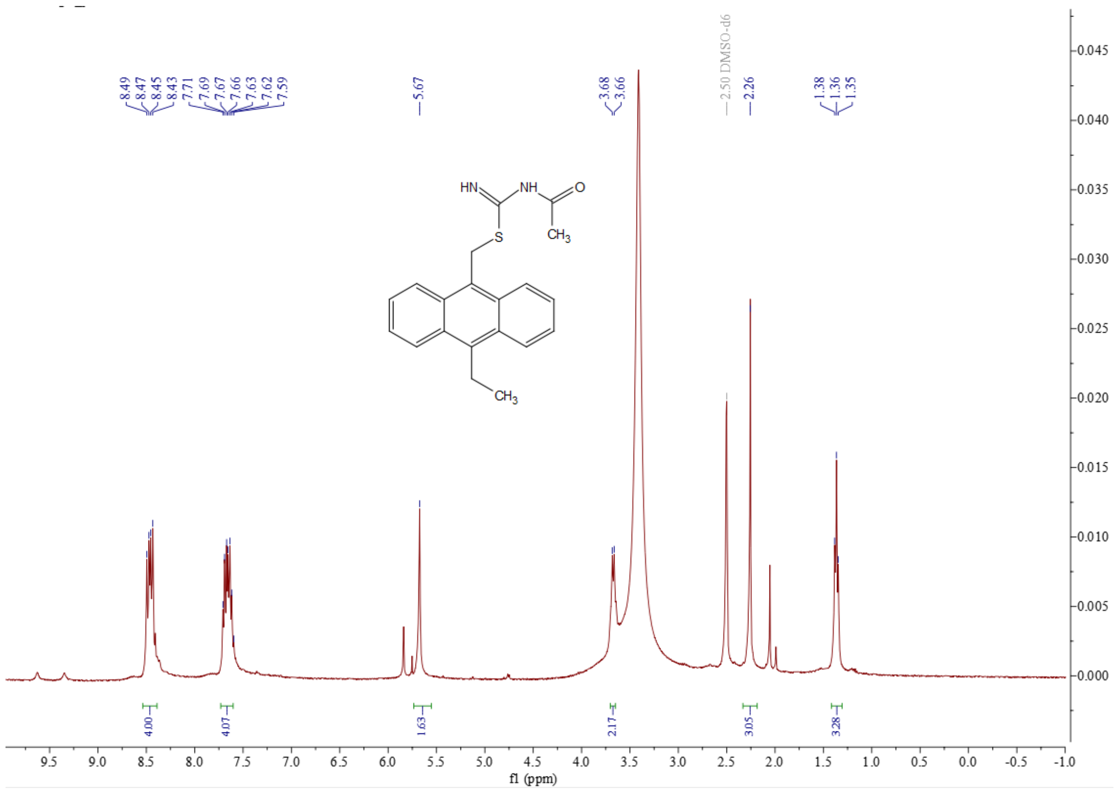

Figure S59 ^{1}H NMR spectrum of **14e**

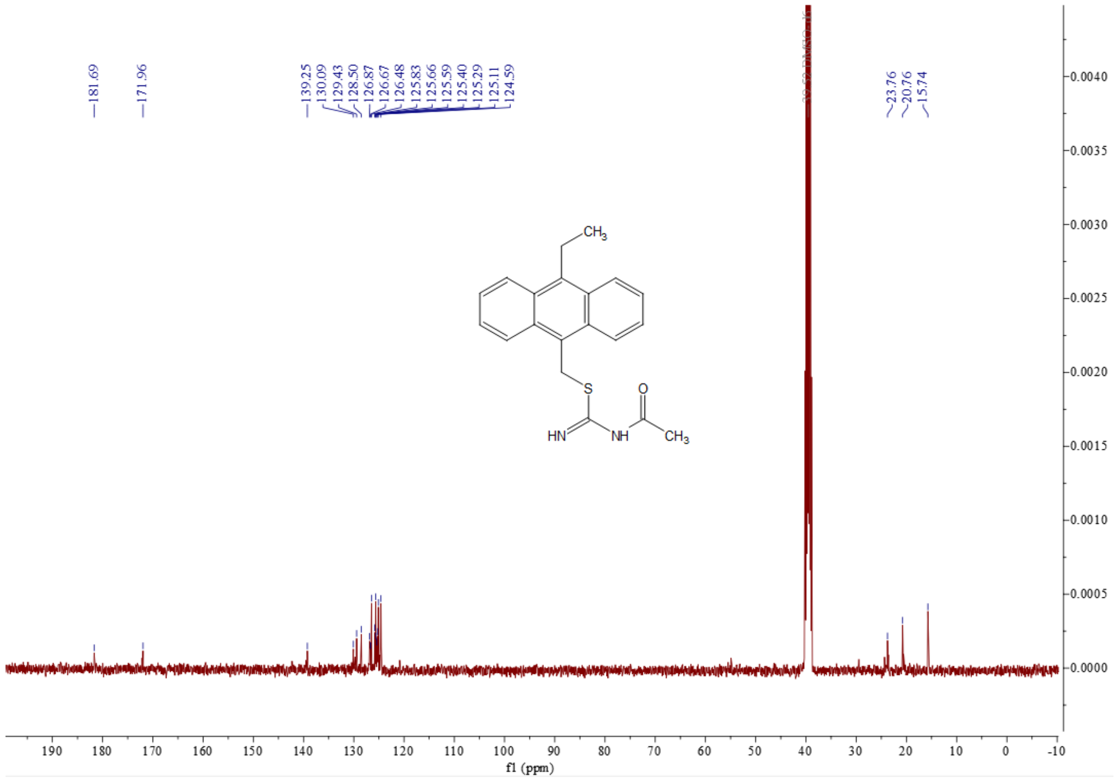

Figure S60 ^{13}C NMR spectrum of **14e**

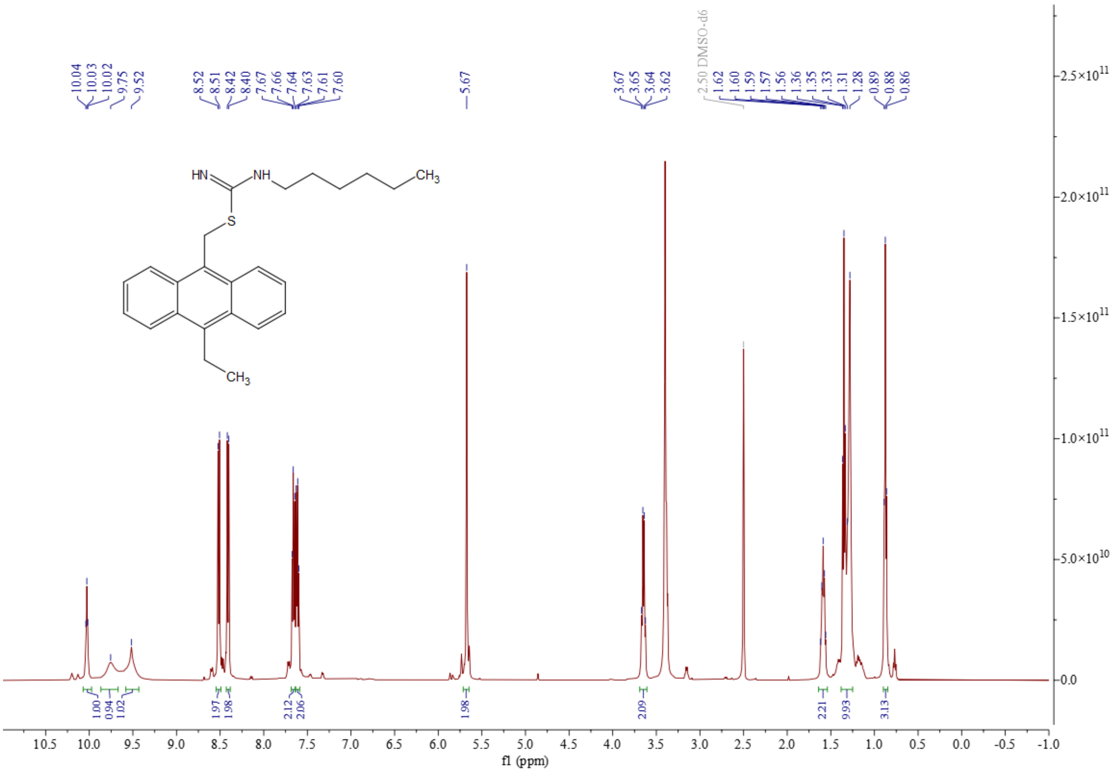

Figure S61 ^{1}H NMR spectrum of **14f**

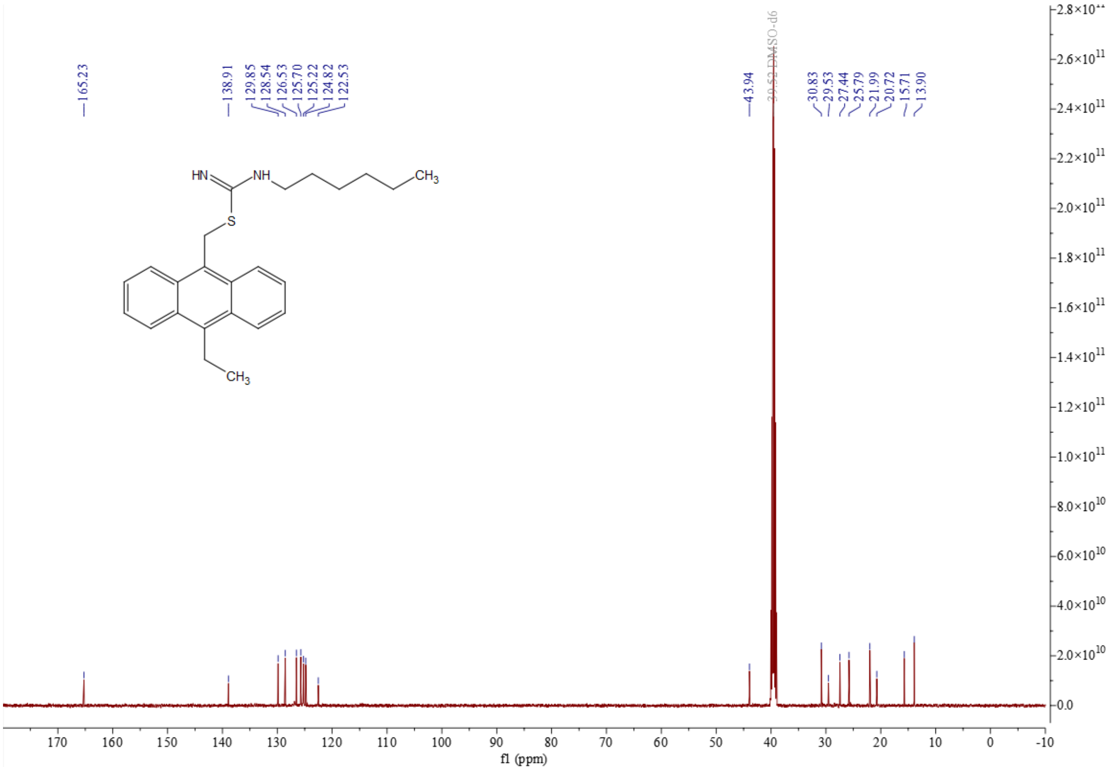

Figure S62 ^{13}C NMR spectrum of **14f**

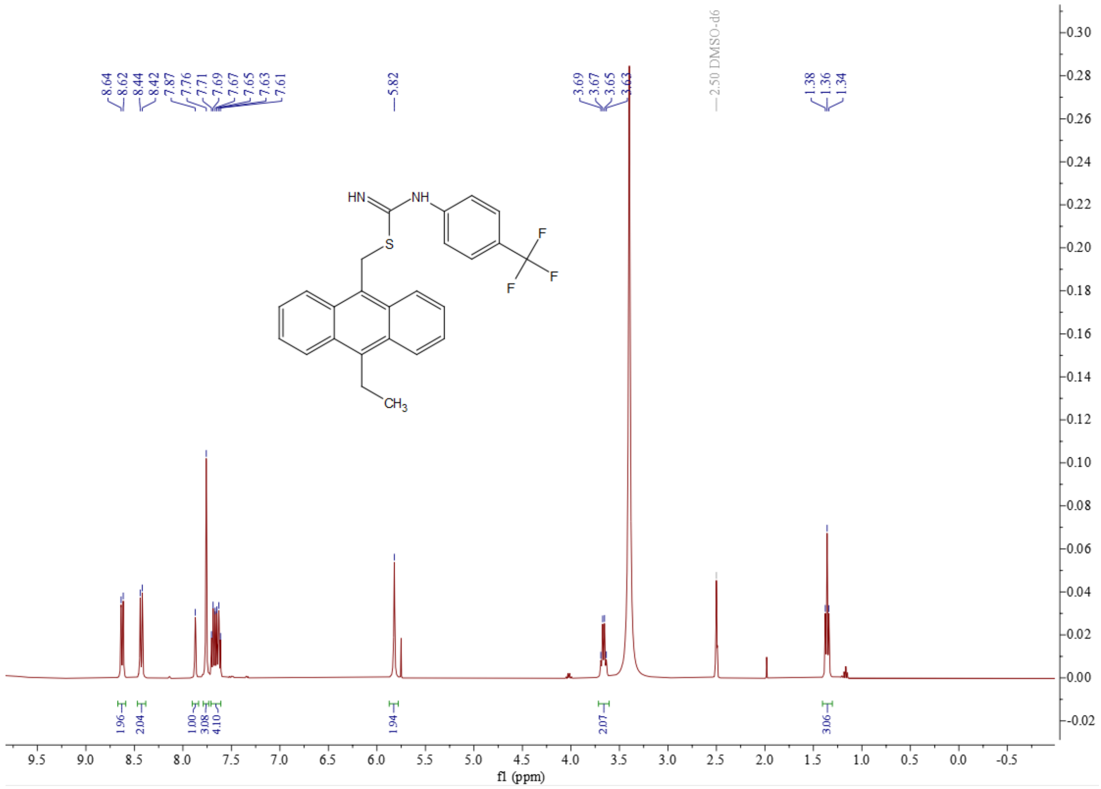

Figure S63 ^{1}H NMR spectrum of **14g**

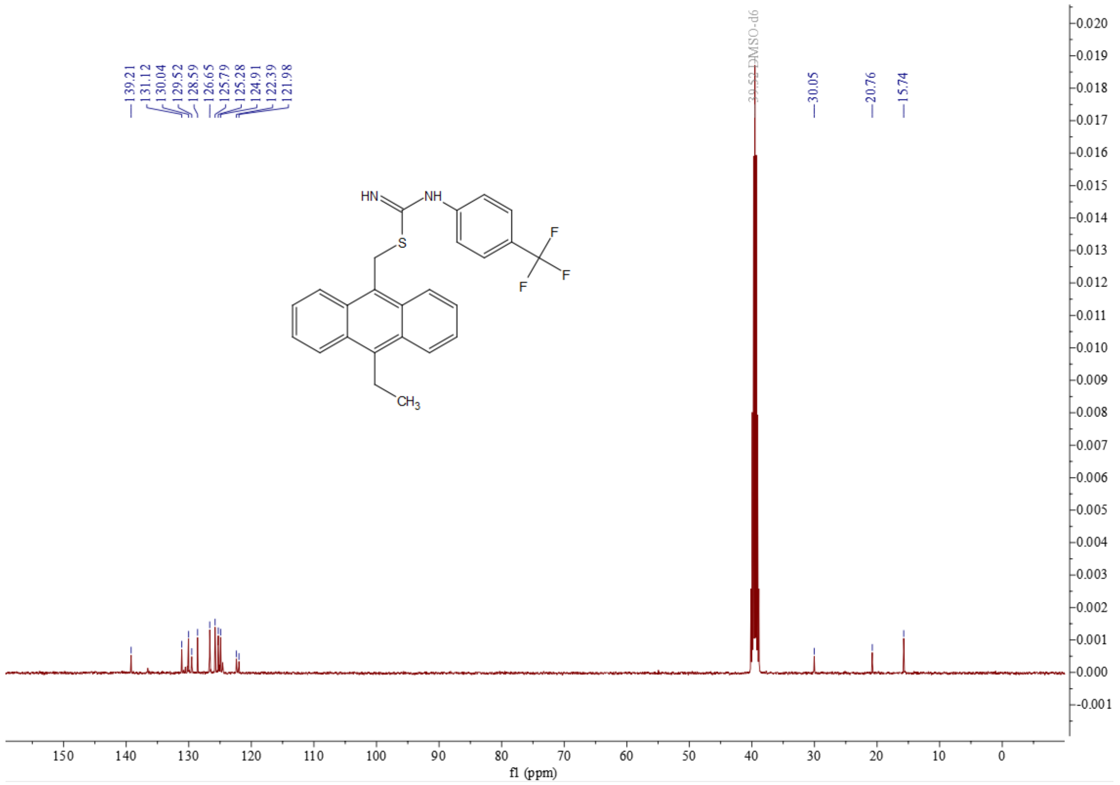

Figure S64 ^{13}C NMR spectrum of **14g**

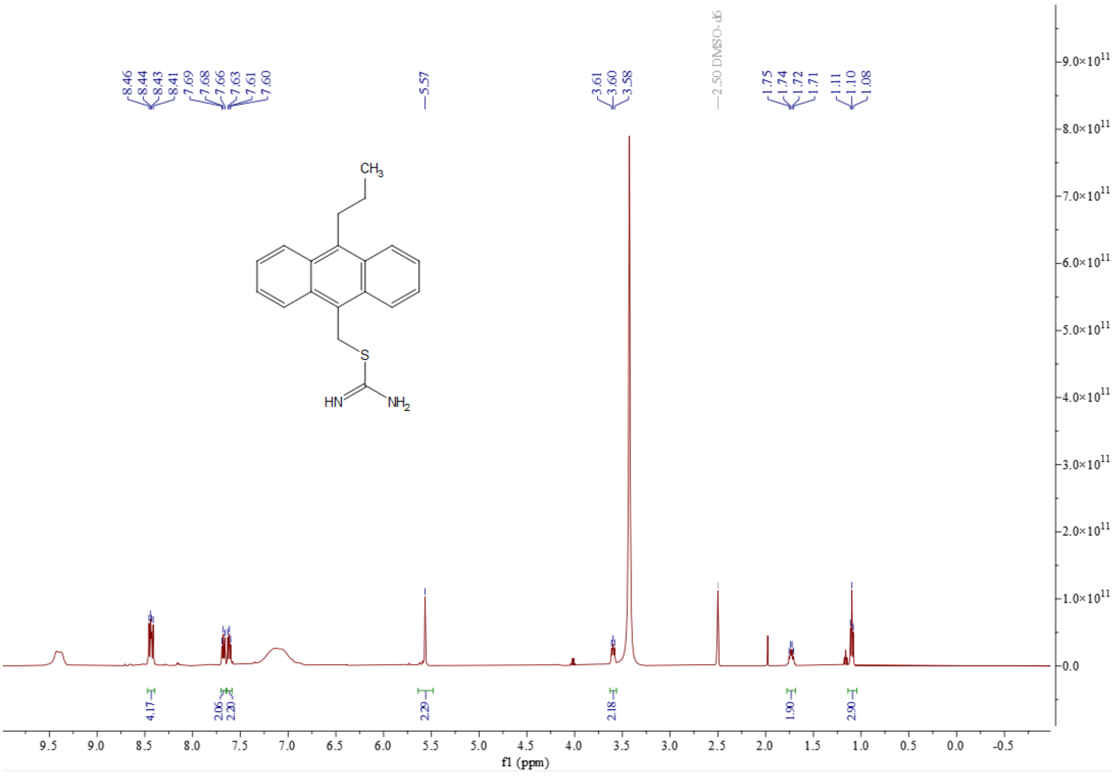

Figure S65 ^{1}H NMR spectrum of **14h**

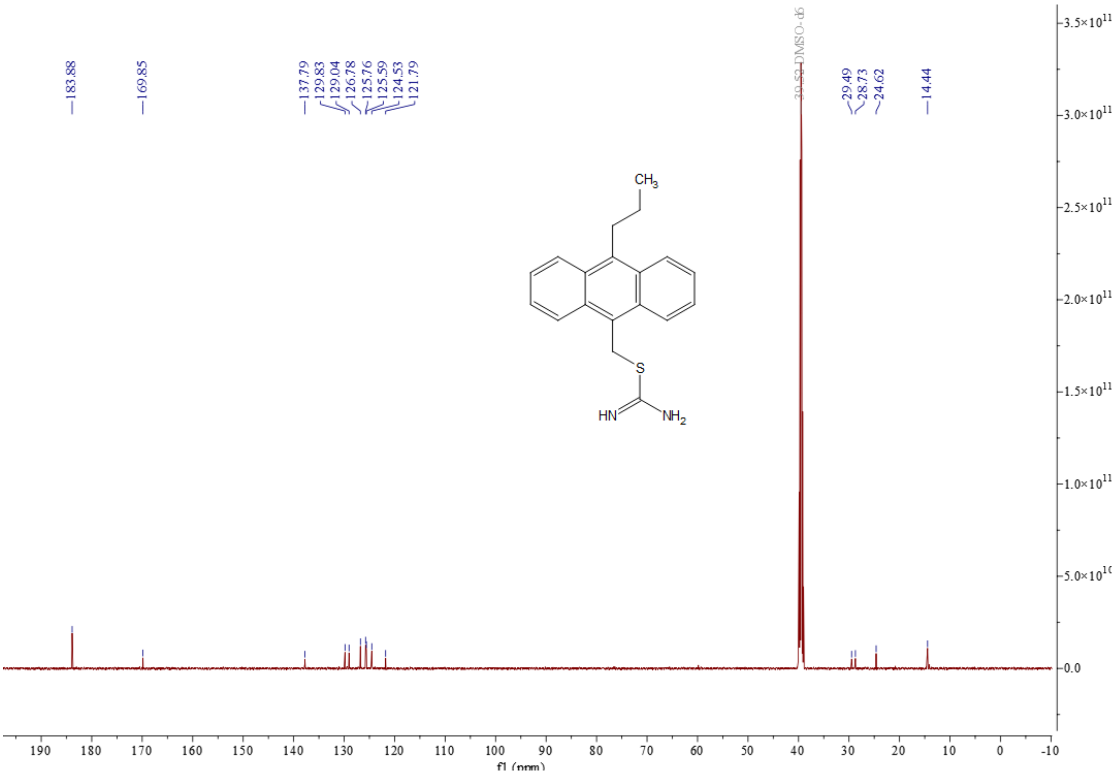

Figure S66 ^{13}C NMR spectrum of **14h**

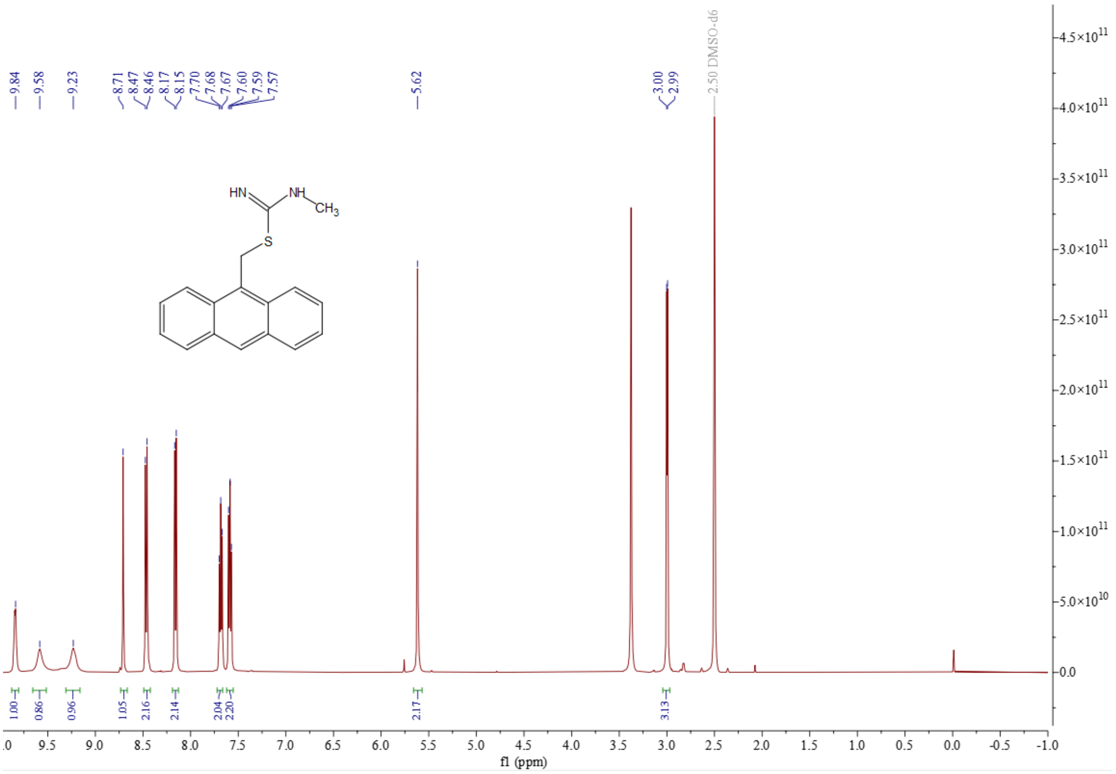

Figure S67 ^{1}H NMR spectrum of **14i**

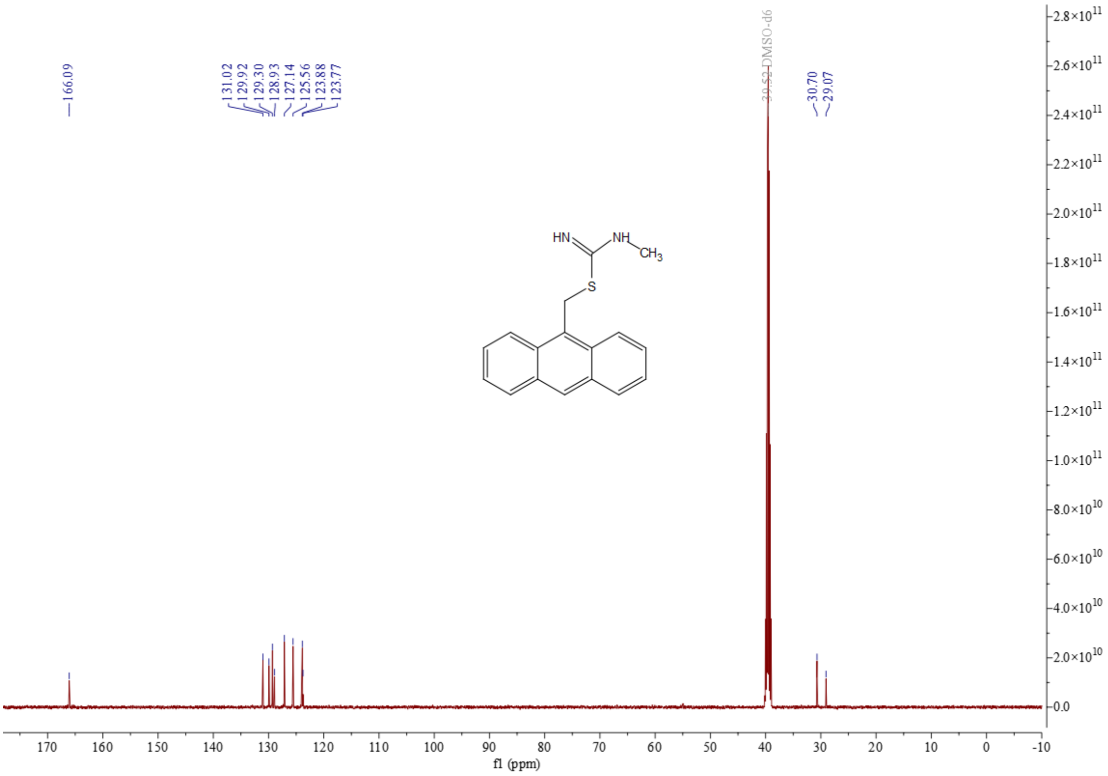

Figure S68 ^{13}C NMR spectrum of **14i**

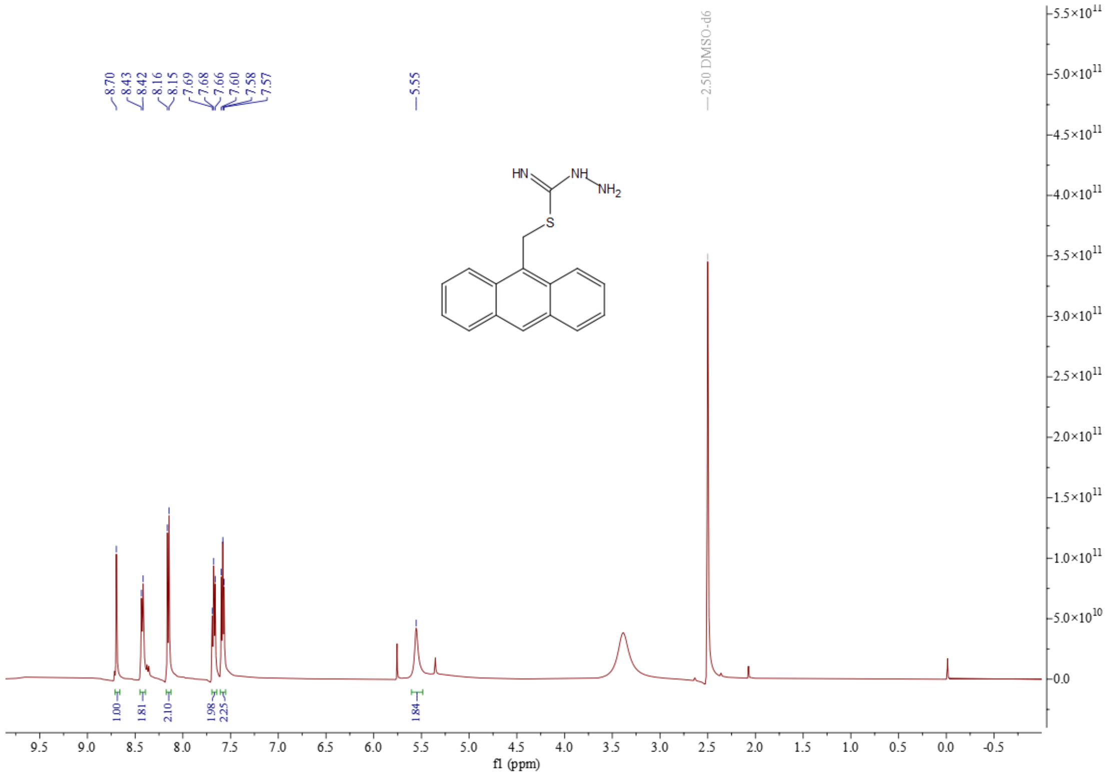

Figure S69 ^{1}H NMR spectrum of **14j**

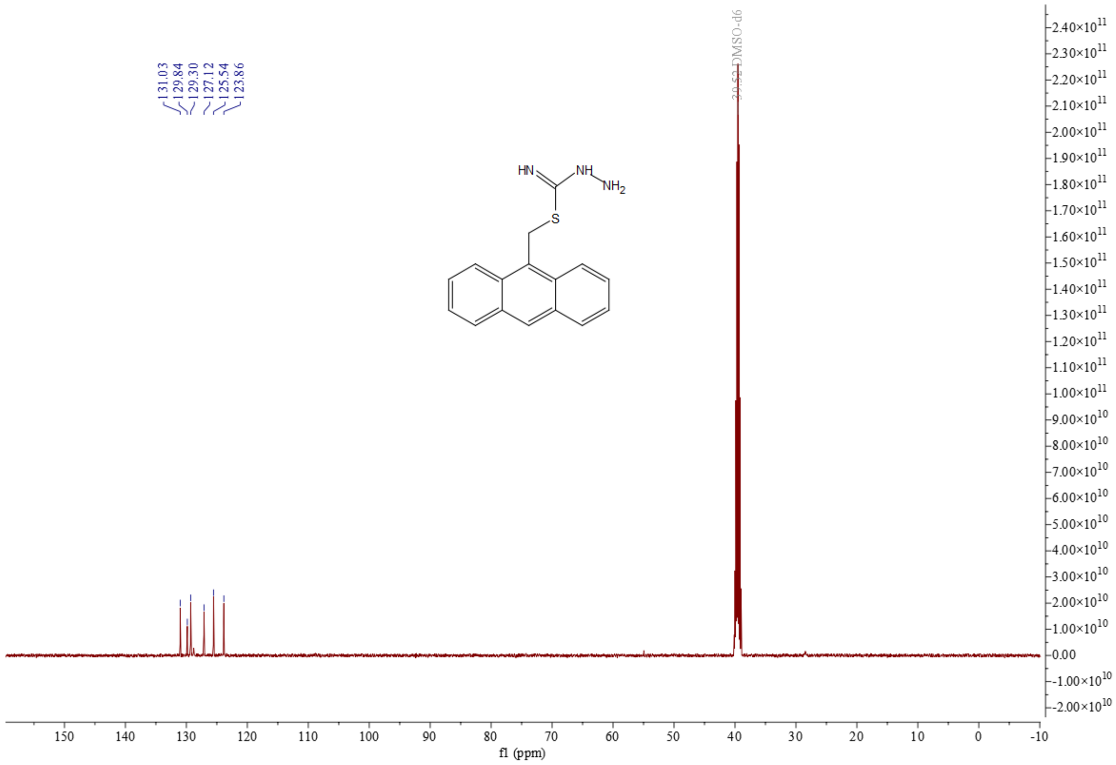

Figure S70 ^{13}C NMR spectrum of **14j**

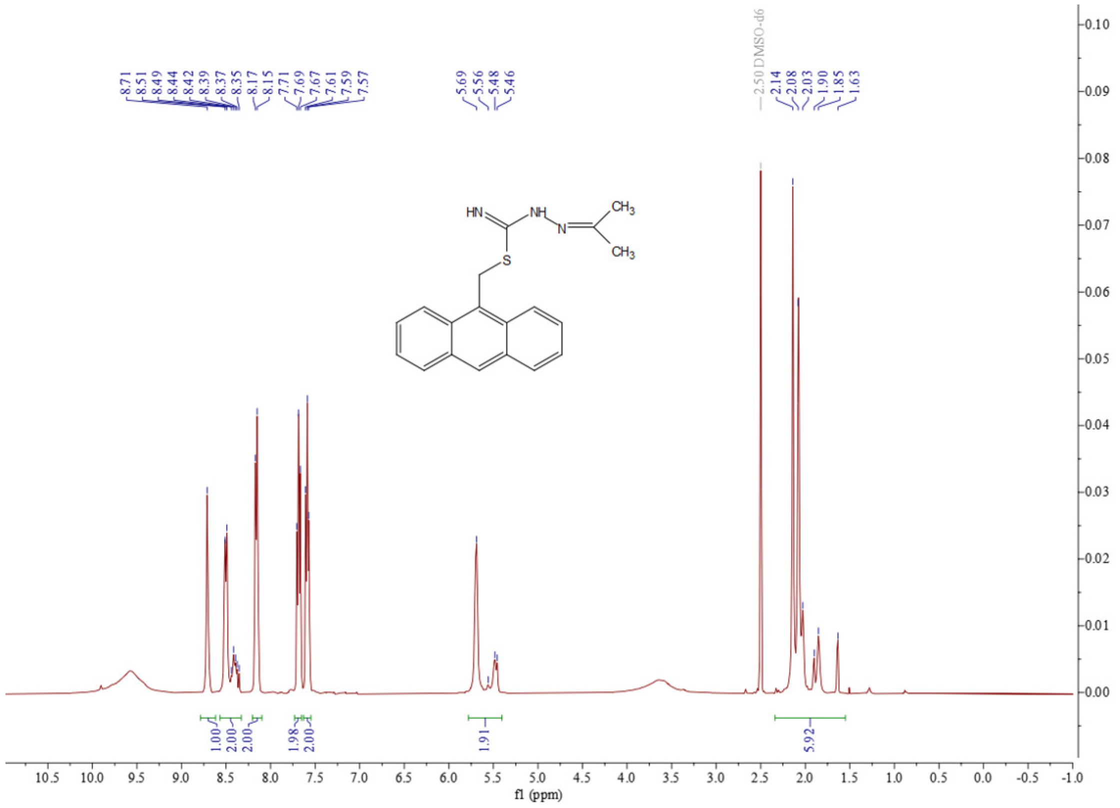

Figure S71 ^{1}H NMR spectrum of **14k**

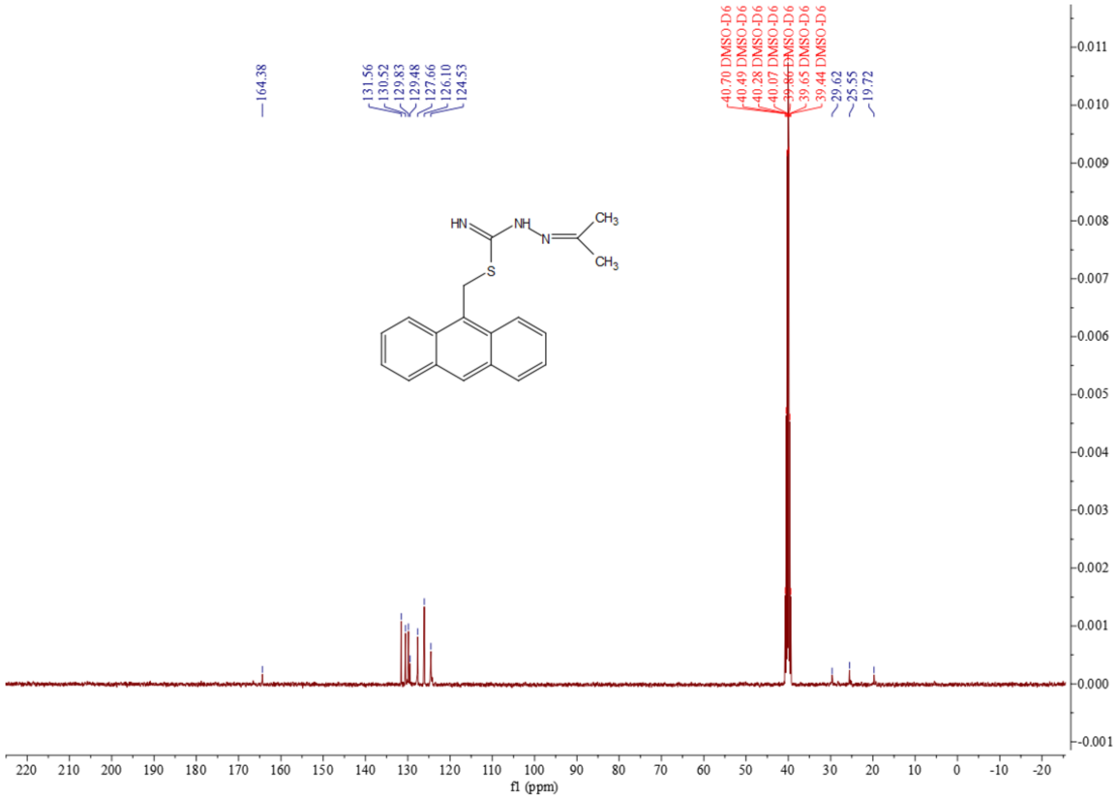

Figure S72 ^{13}C NMR spectrum of **14k**

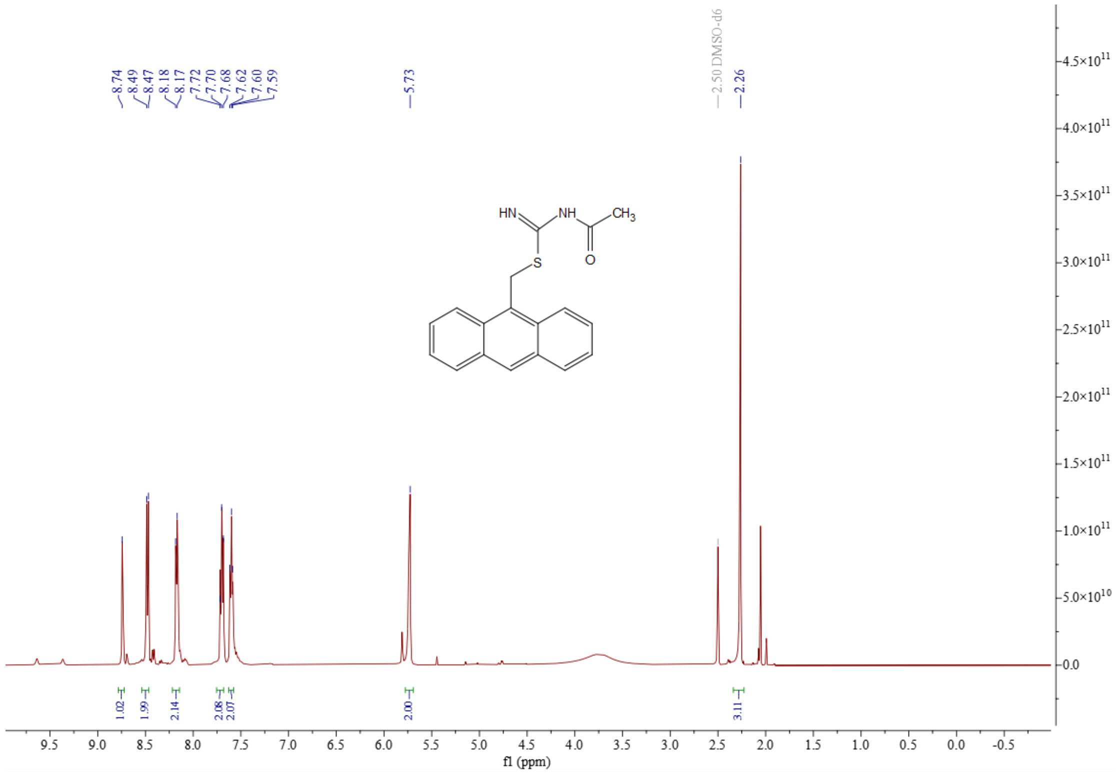

Figure S73 ^{1}H NMR spectrum of **14l**

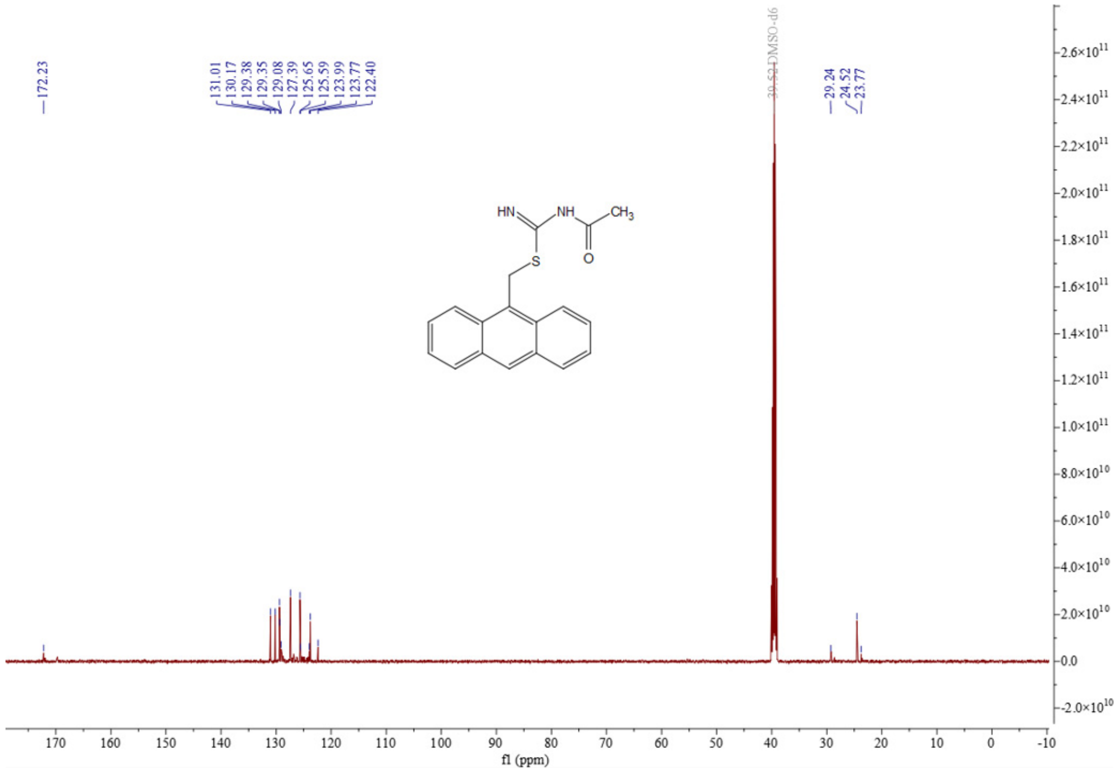

Figure S74 ^{13}C NMR spectrum of **14l**

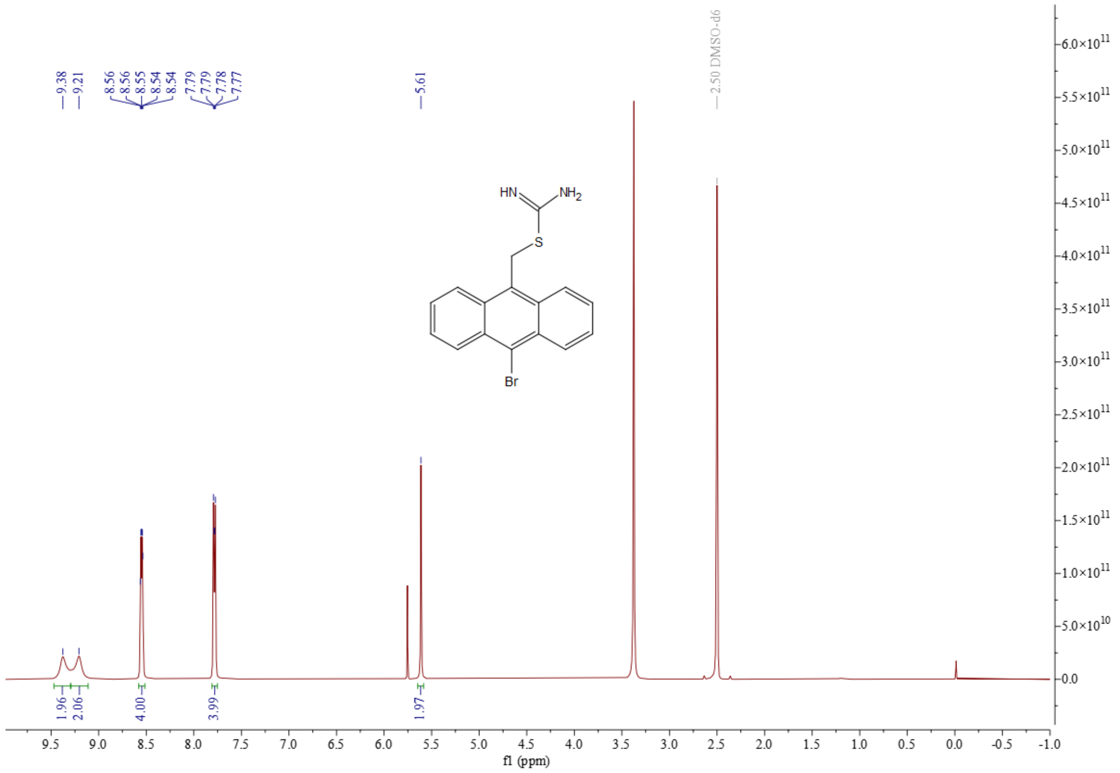

Figure S75 ^{1}H NMR spectrum of **14m**

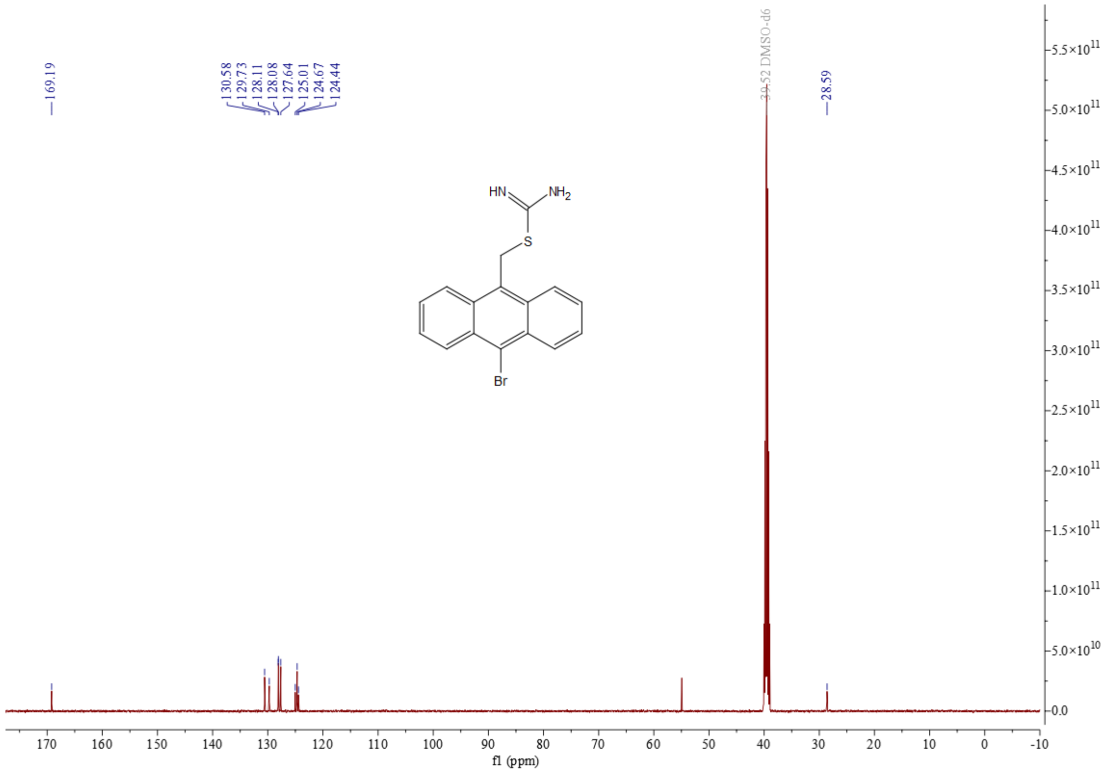

Figure S76 ^{13}C NMR spectrum of **14m**

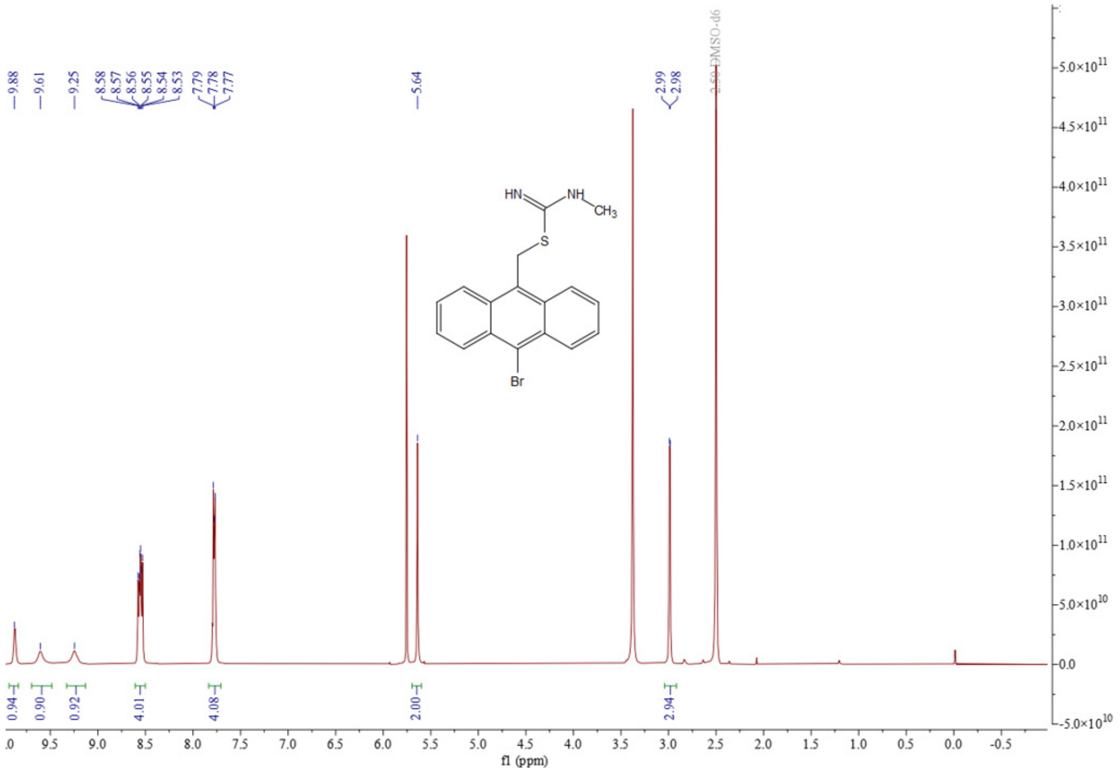

Figure S77 ^{1}H NMR spectrum of **14n**

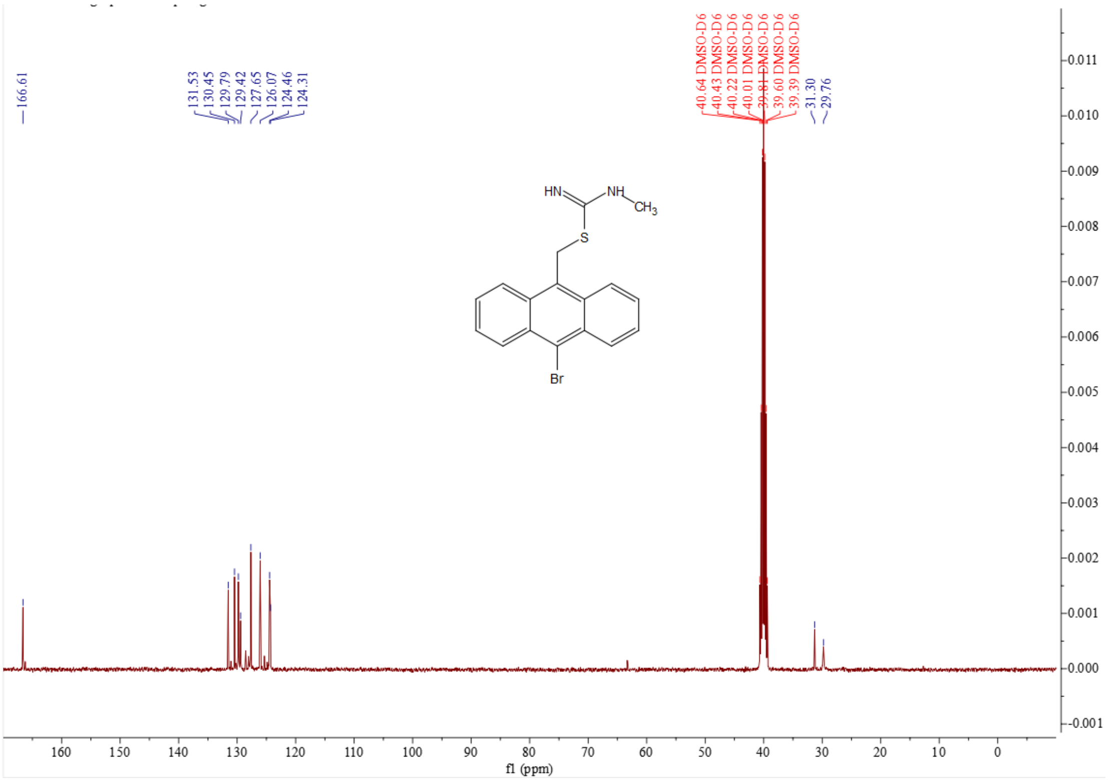

Figure S78 ^{13}C NMR spectrum of **14n**

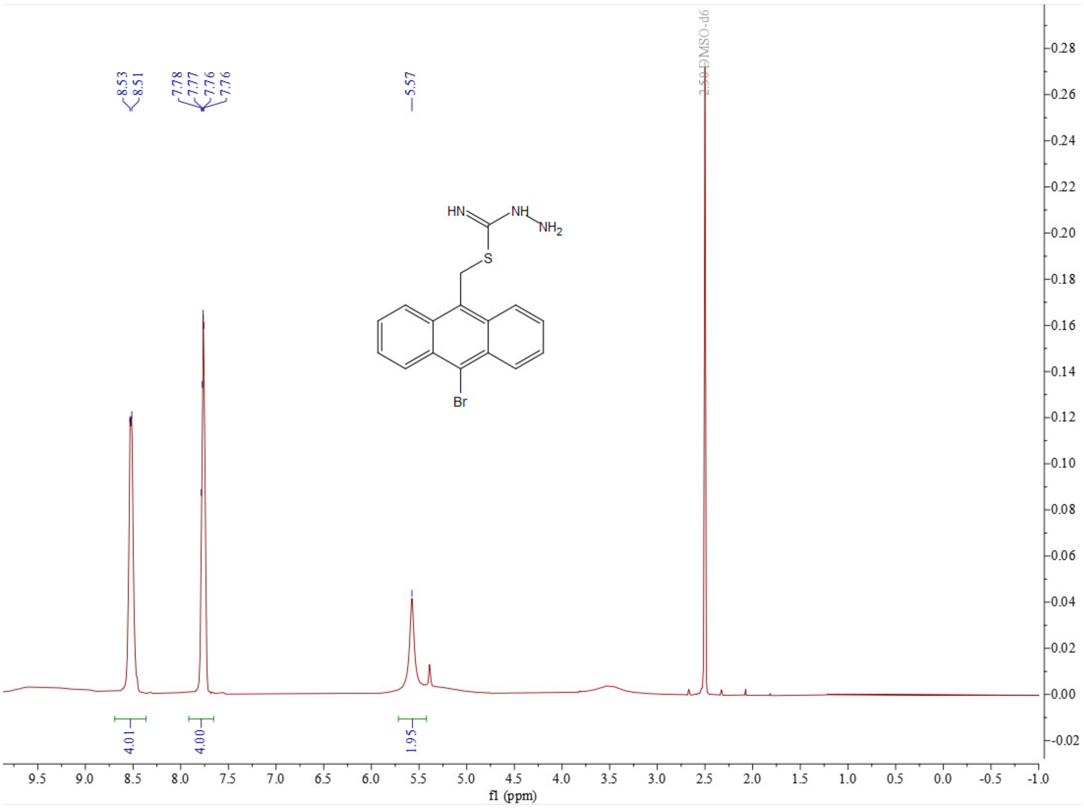

Figure S79 ^{1}H NMR spectrum of **14o**

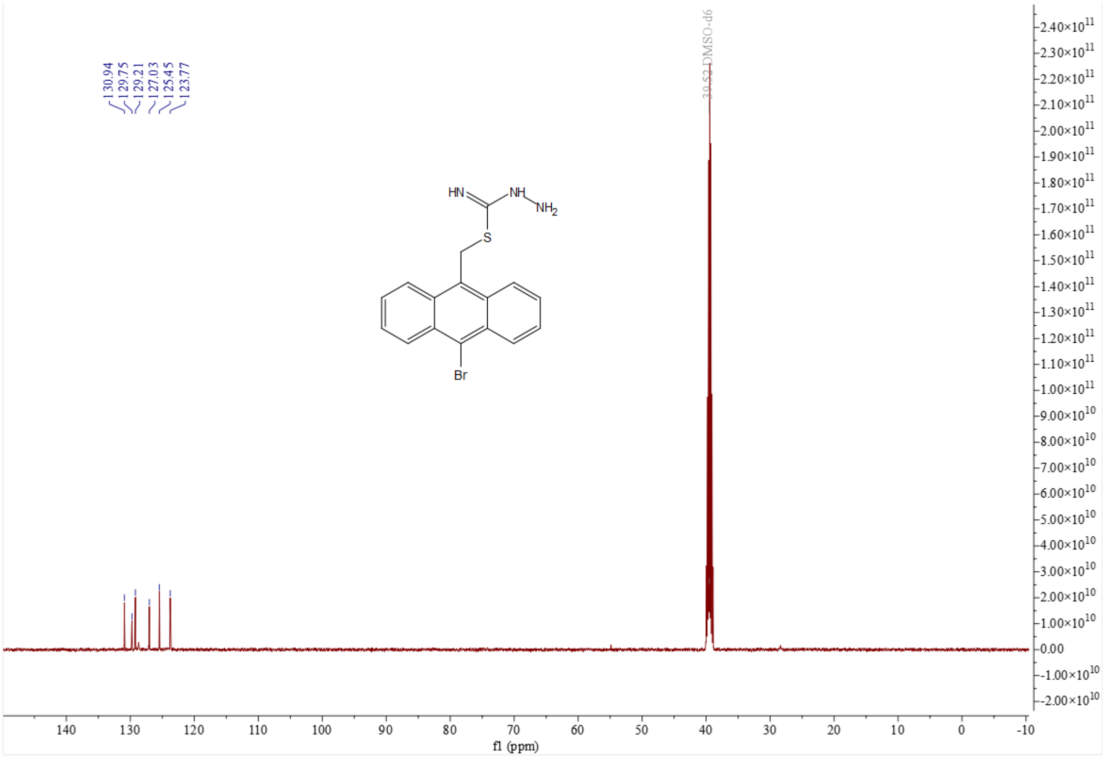

Figure S80 ^{13}C NMR spectrum of **14o**

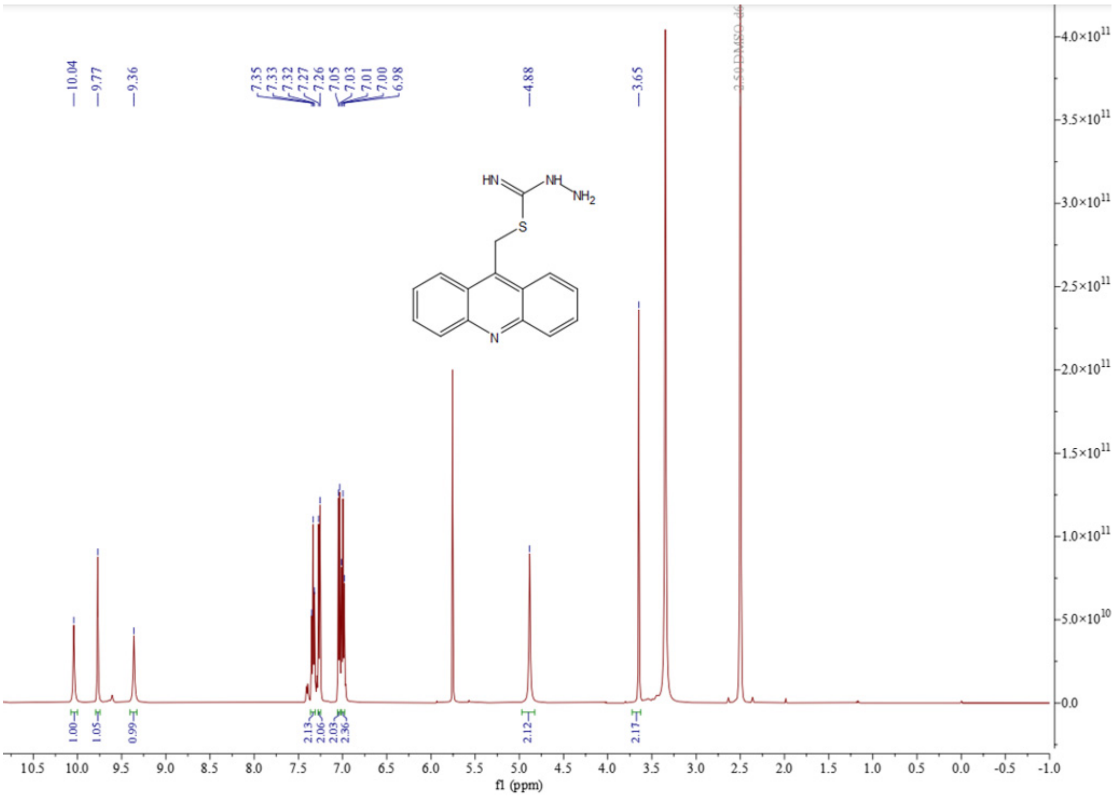

Figure S81 ^{1}H NMR spectrum of **14p**

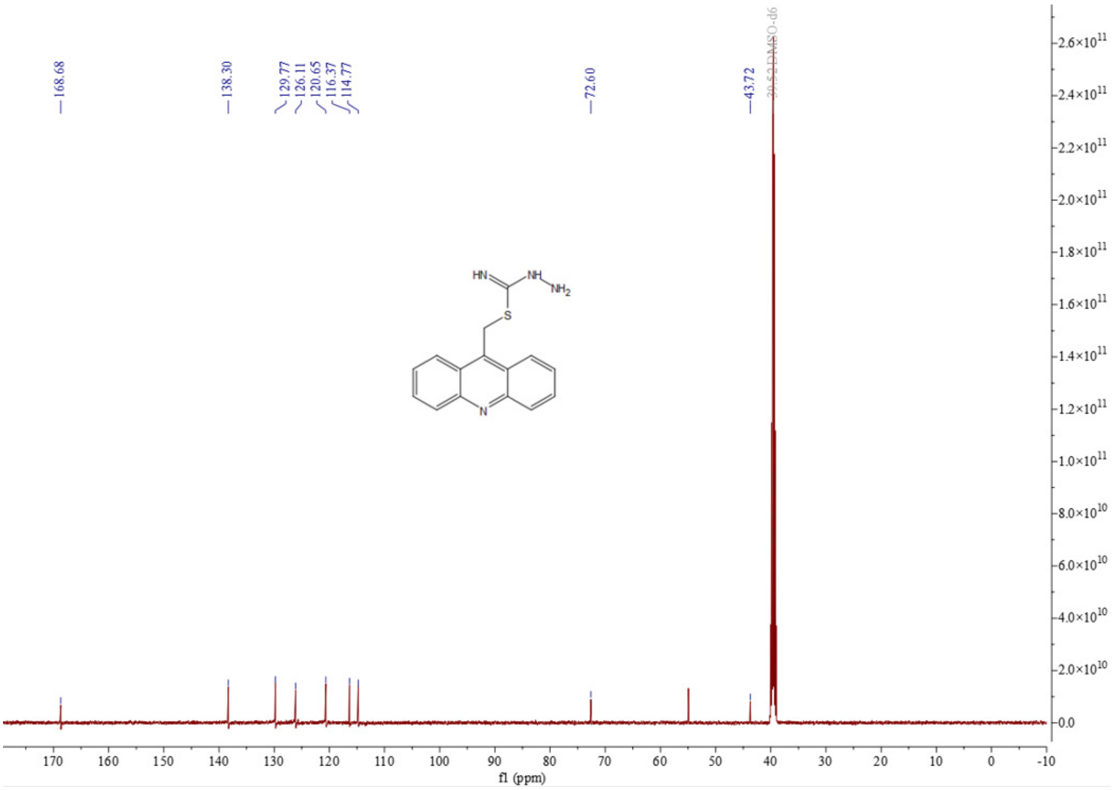

Figure S82 ^{13}C NMR spectrum of **14p**

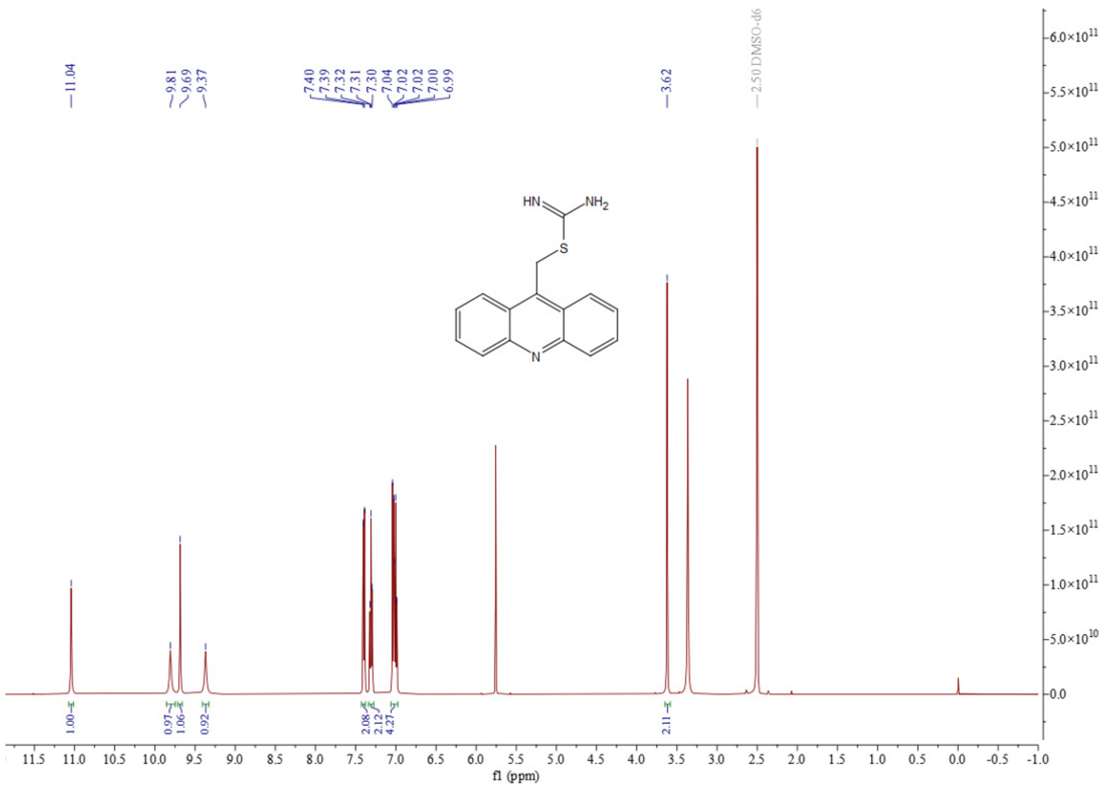

Figure S83 ^{1}H NMR spectrum of **14q**

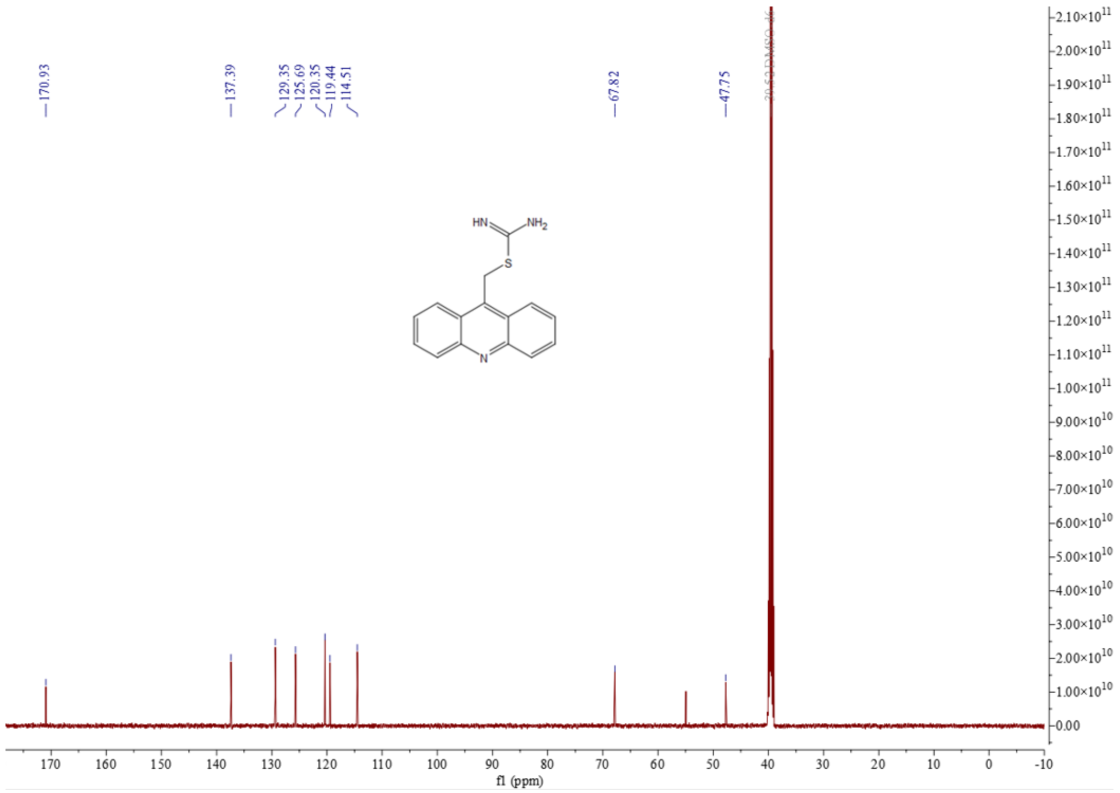

Figure S84 ^{13}C NMR spectrum of **14q**

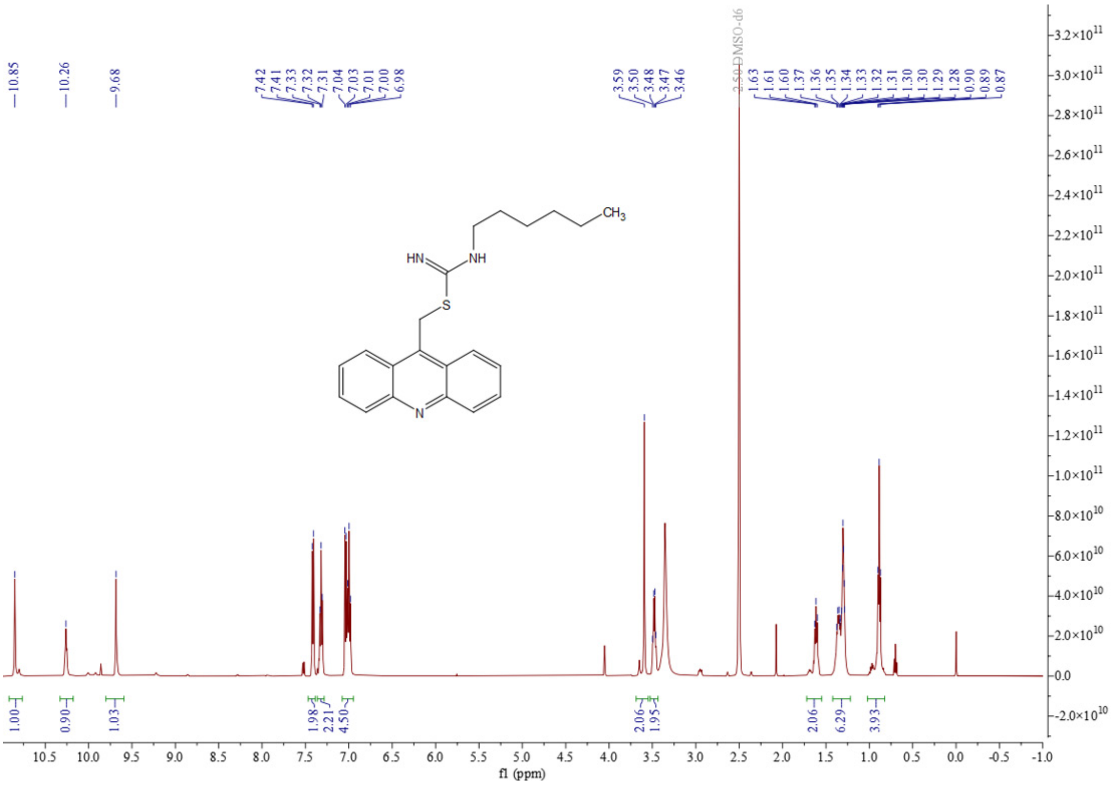

Figure S85 ^{1}H NMR spectrum of **14r**

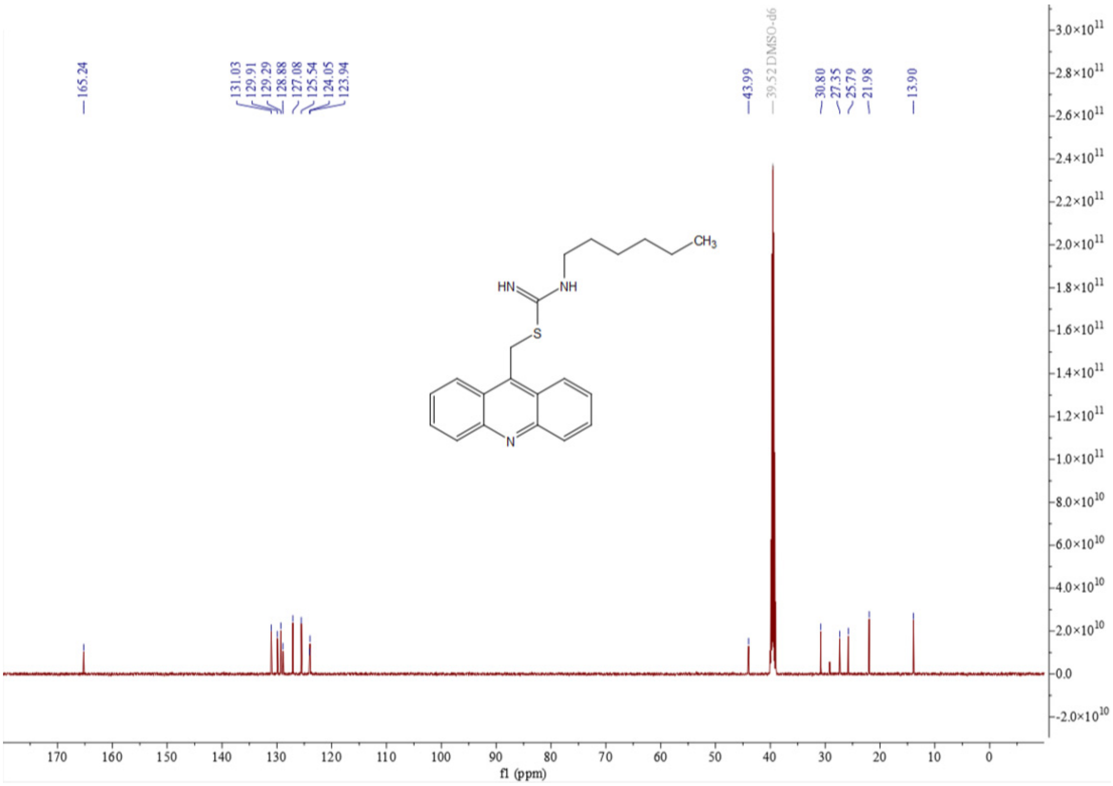

Figure S86 ^{13}C NMR spectrum of **14r**

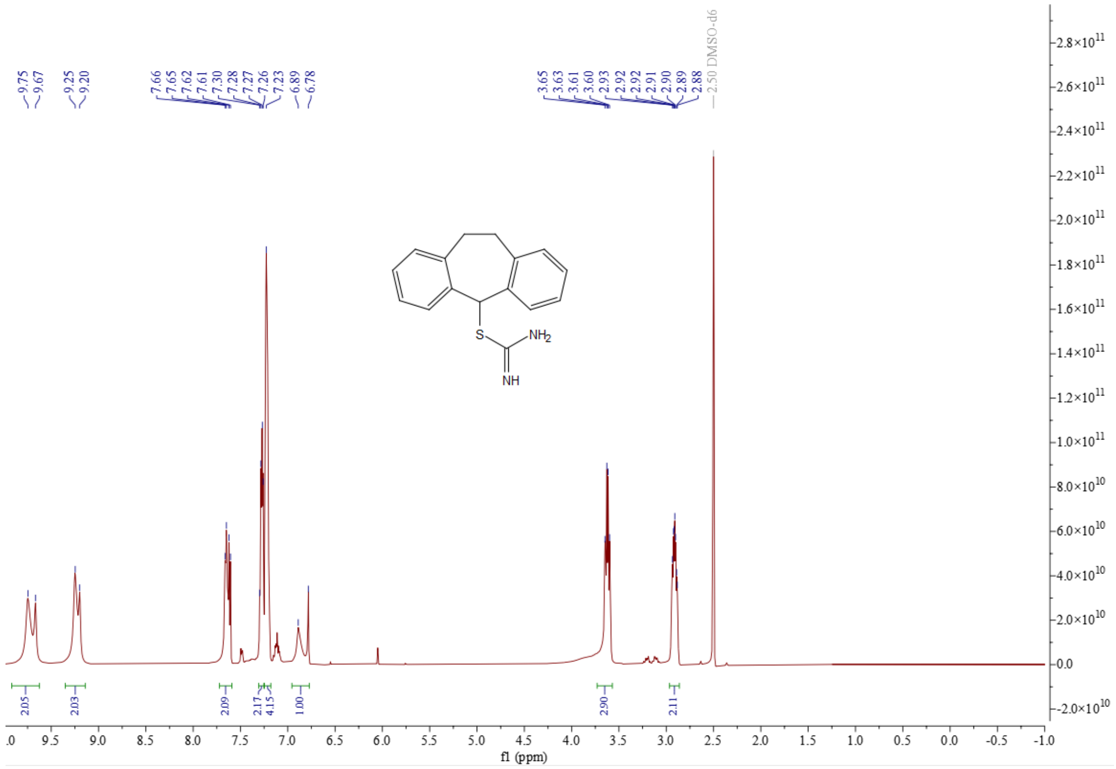

Figure S87 ^{1}H NMR spectrum of **14s**

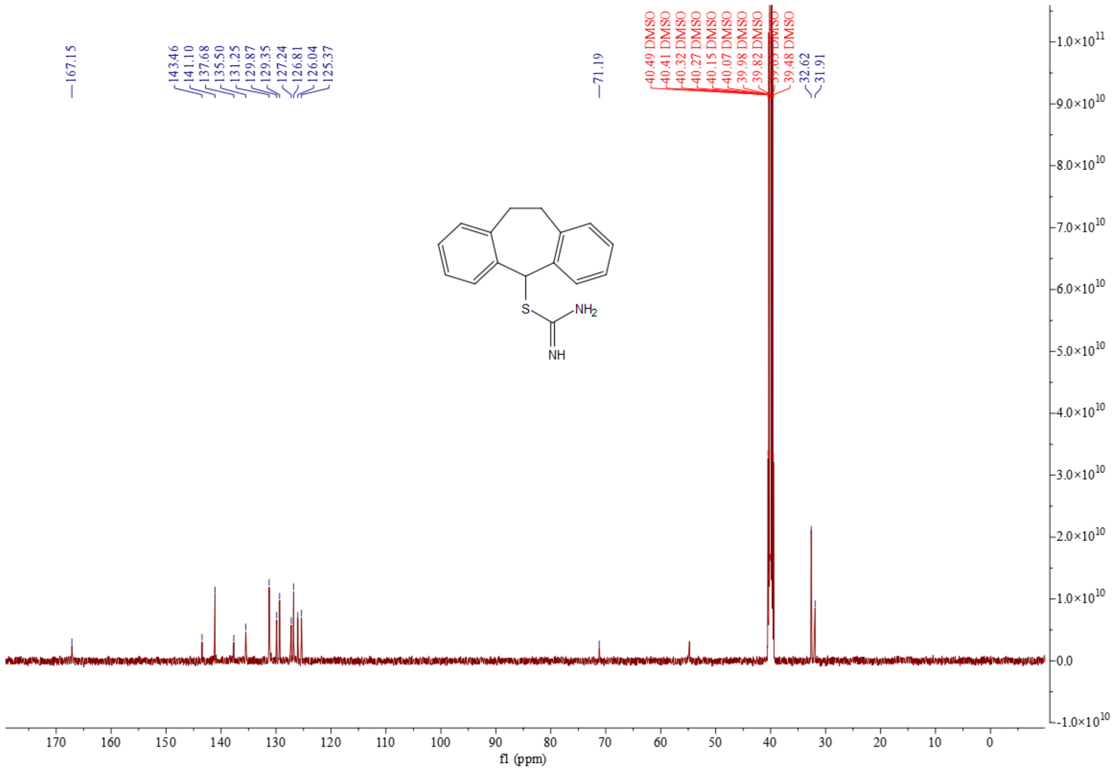

Figure S88 ^{13}C NMR spectrum of **14s**

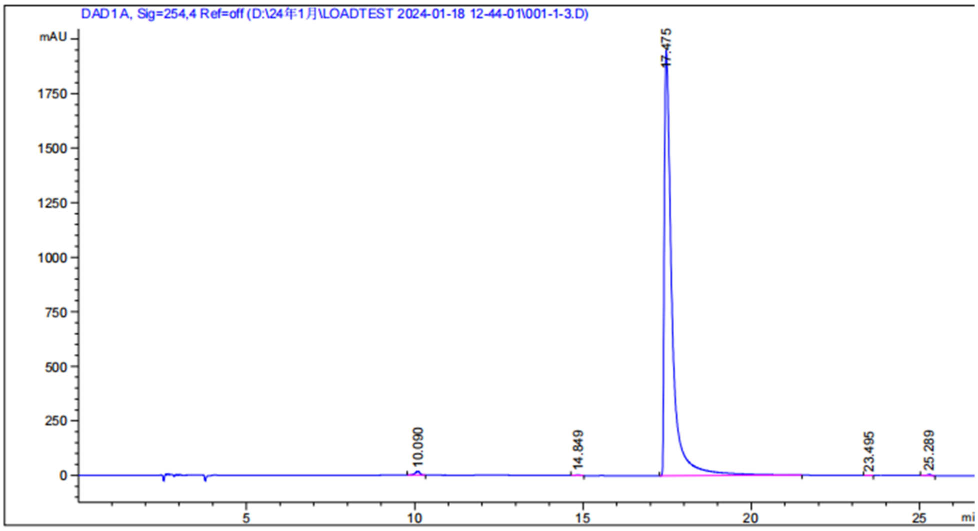

==
面积百分比报告
==

排序 : 信号
乘积因子 : 1.0000
稀释因子 : 1.0000
内标使用乘积因子和稀释因子

信号 1: DAD1 A, Sig=254,4 Ref=off

峰 #	保留时间 [min]	类型	峰宽 [min]	峰面积 [mAU*s]	峰高 [mAU]	峰面积 %
1	10.090	BB	0.1450	171.09261	17.98687	0.5492
2	14.849	BB	0.1239	29.31156	3.64301	0.0941
3	17.475	BB	0.2348	3.09070e4	1951.40308	99.2030
4	23.495	BB	0.1044	9.17282	1.29830	0.0294
5	25.289	BB	0.1325	38.73014	4.32314	0.1243

总量 : 3.11553e4 1978.65439

Figure S89 HPLC spectra of **13e**

4. Dose–response curves of compounds on the cytotoxicity of U87-MG, Hela, MCF-7, and HHL-5 cells **S58**

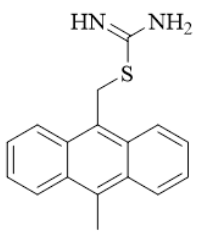

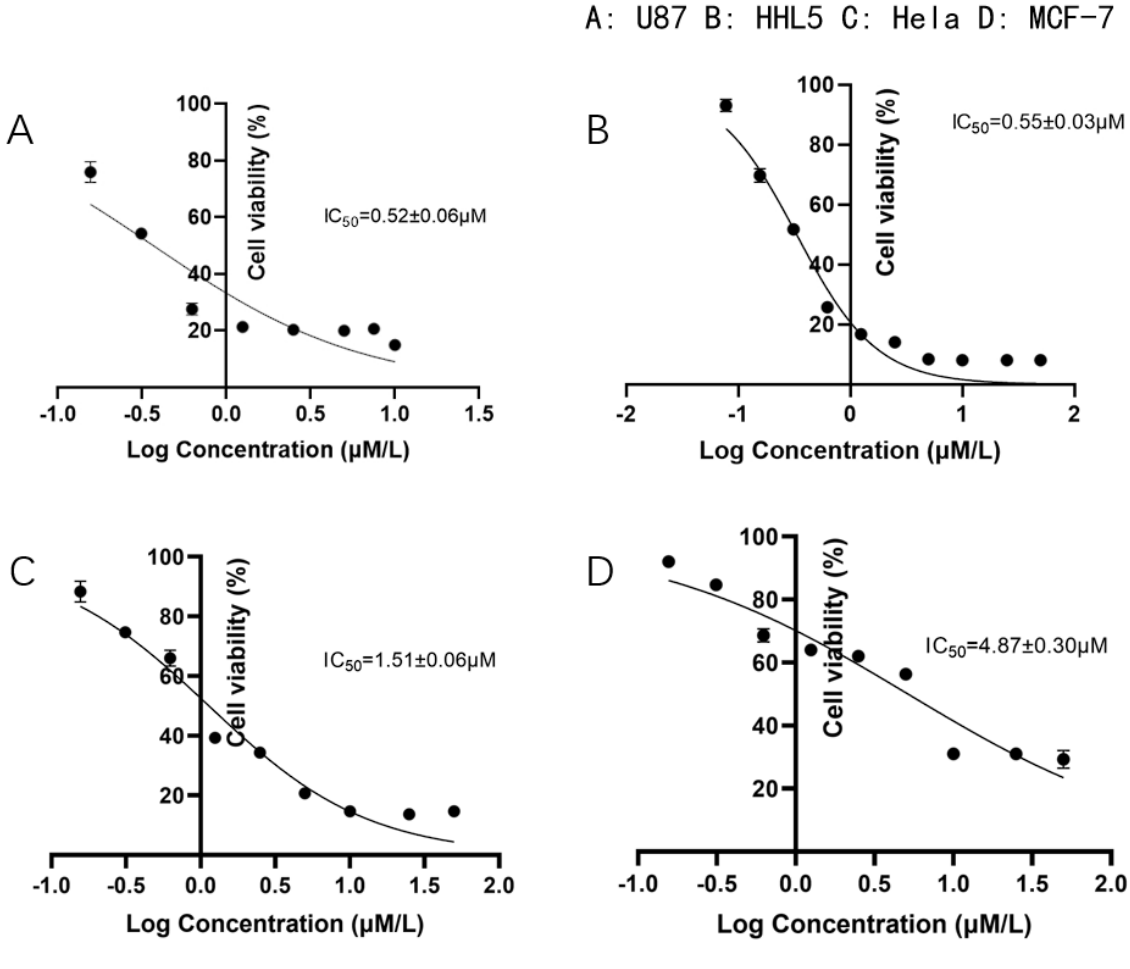

Figure S89 Dose–response curves of XI-011 on the cytotoxicity of U87-MG, Hela, MCF-7, and HHL-5 cells

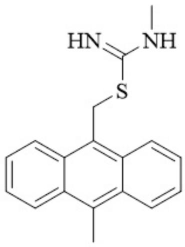

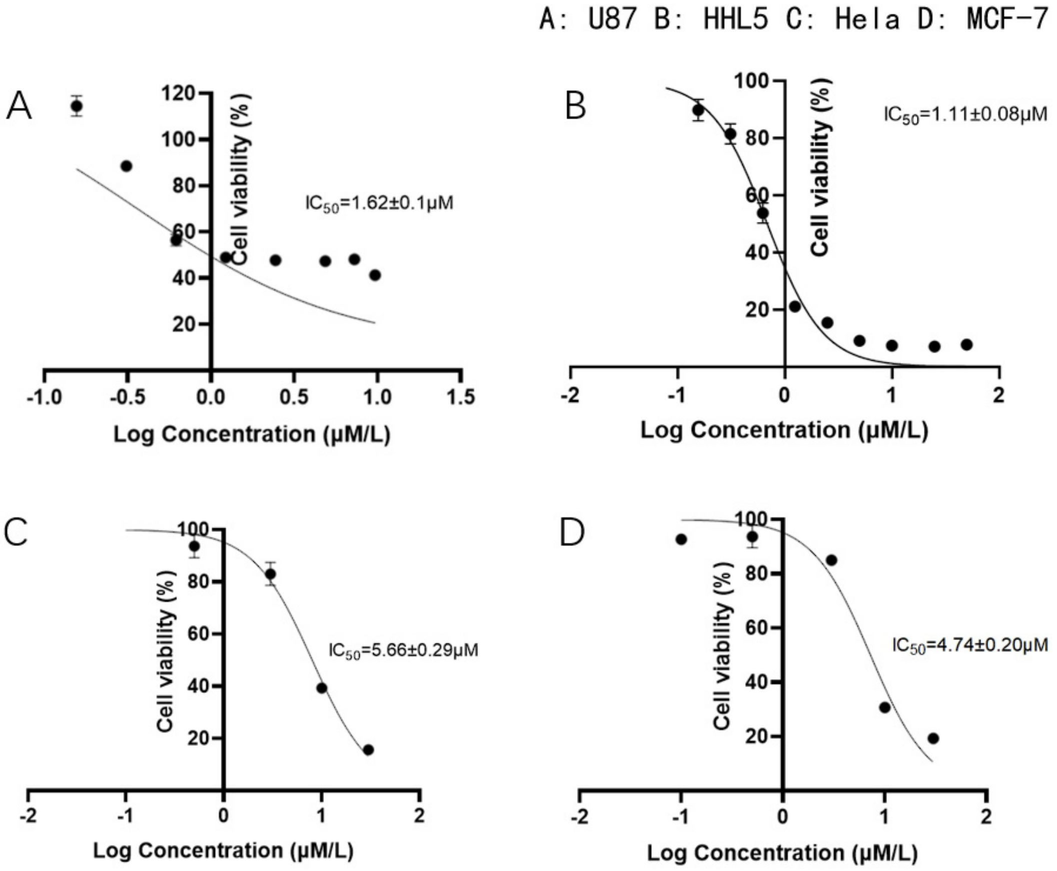

Figure S90 Dose–response curves of **13d** on the cytotoxicity of U87-MG, Hela, MCF-7, and HHL-5 cells

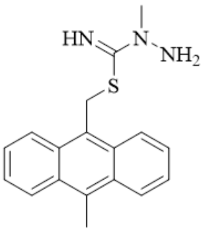

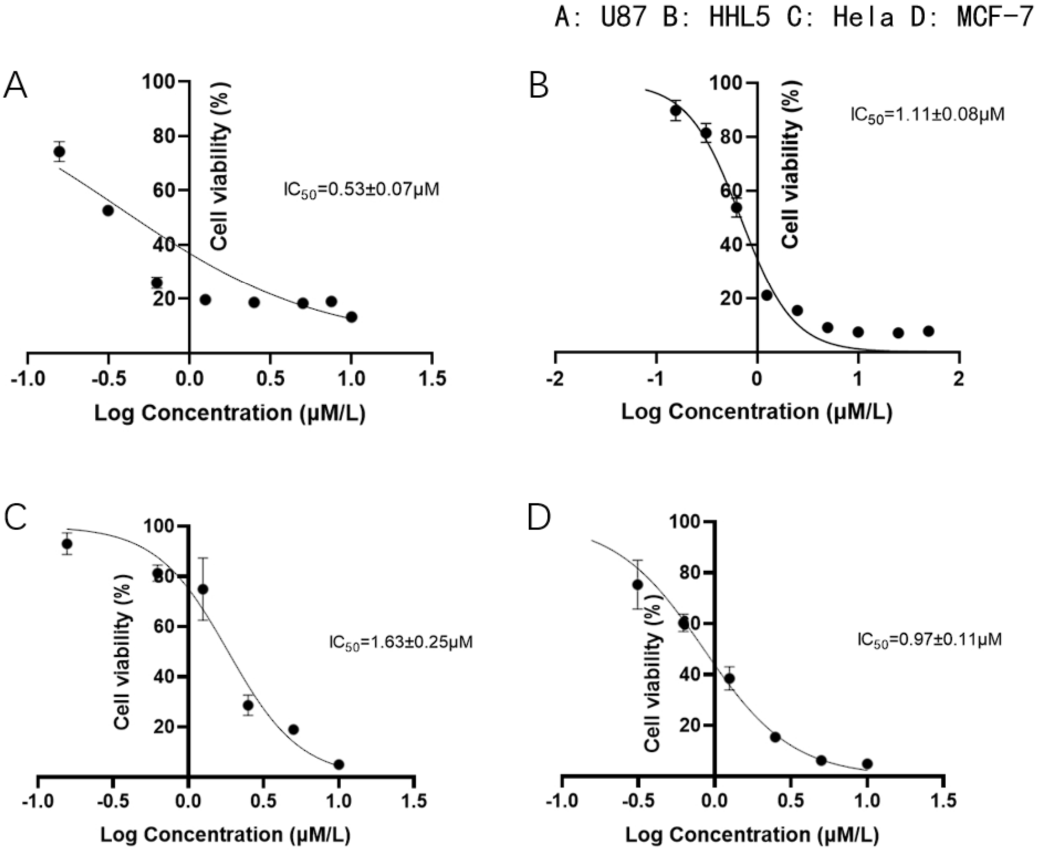

Figure S91 Dose–response curves of **13e** on the cytotoxicity of U87-MG, Hela, MCF-7, and HHL-5 cells

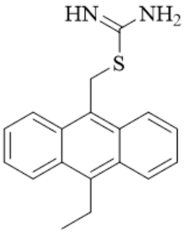

A: U87 B: HHL5 C: Hela D: MCF-7

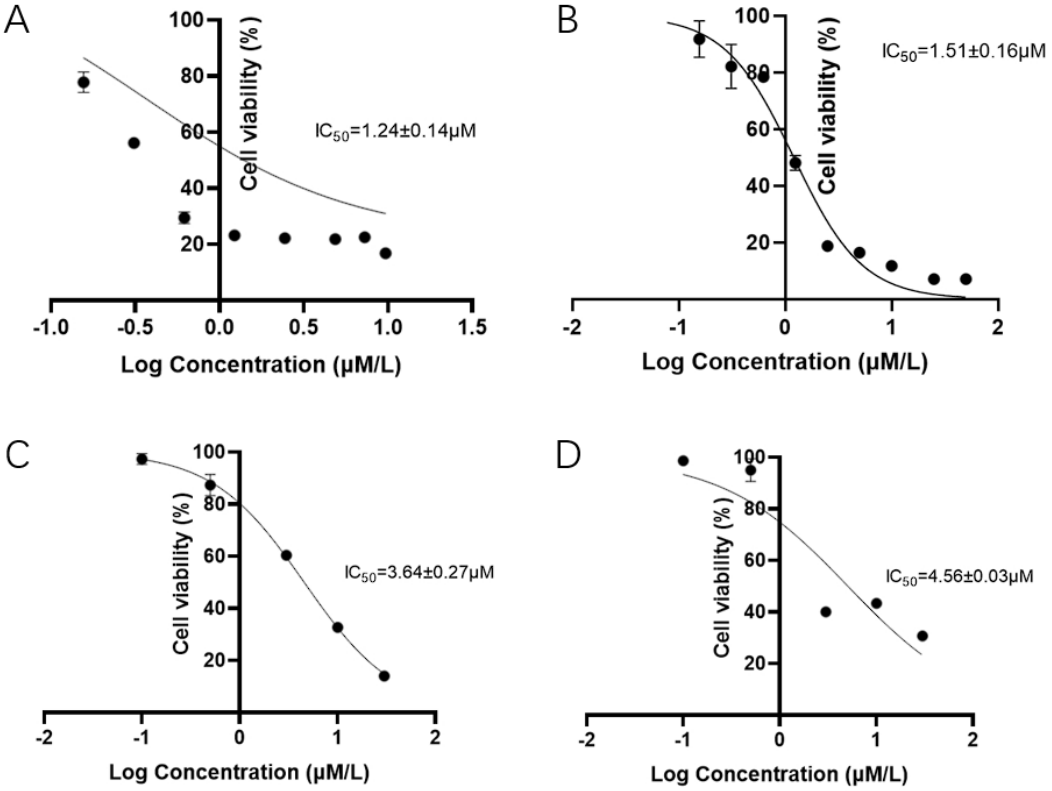

Figure S92 Dose–response curves of **14a** on the cytotoxicity of U87-MG, Hela, MCF-7, and HHL-5 cells

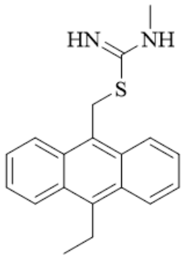

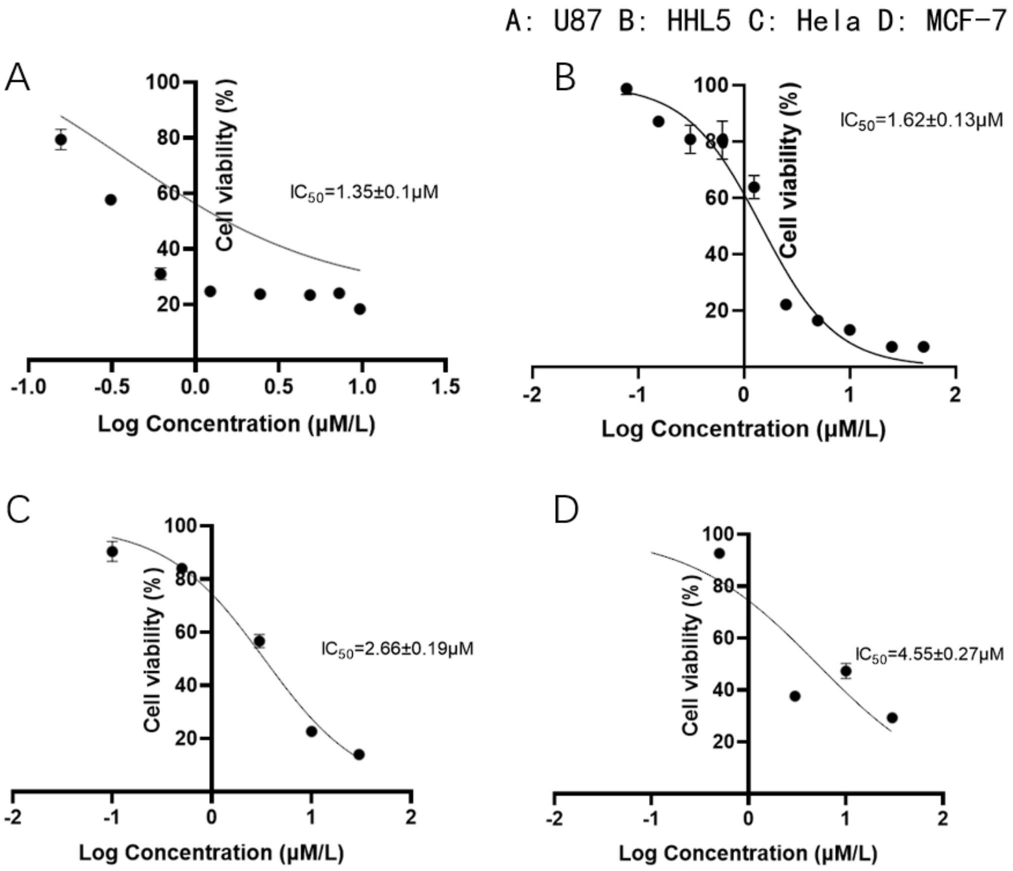

Figure S93 Dose–response curves of **14b** on the cytotoxicity of U87-MG, Hela, MCF-7, and HHL-5 cells

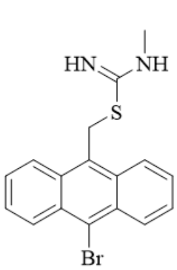

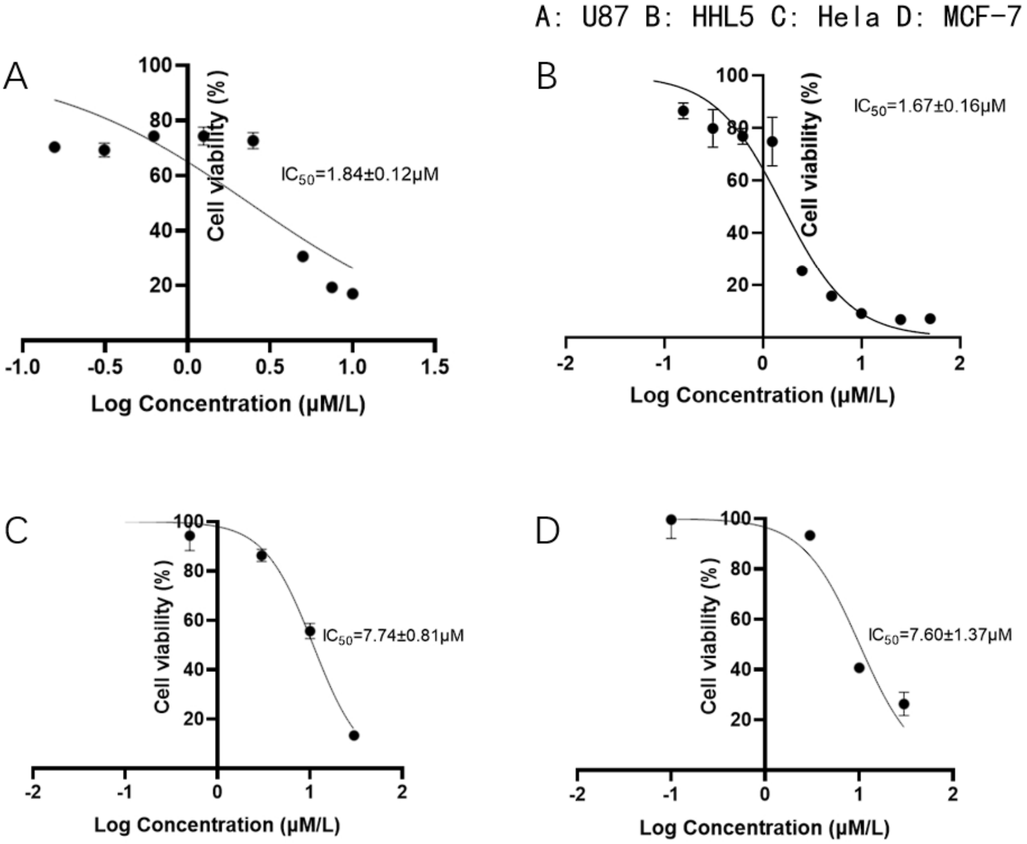

Figure S94 Dose–response curves of **14n** on the cytotoxicity of U87-MG, Hela, MCF-7, and HHL-5 cells

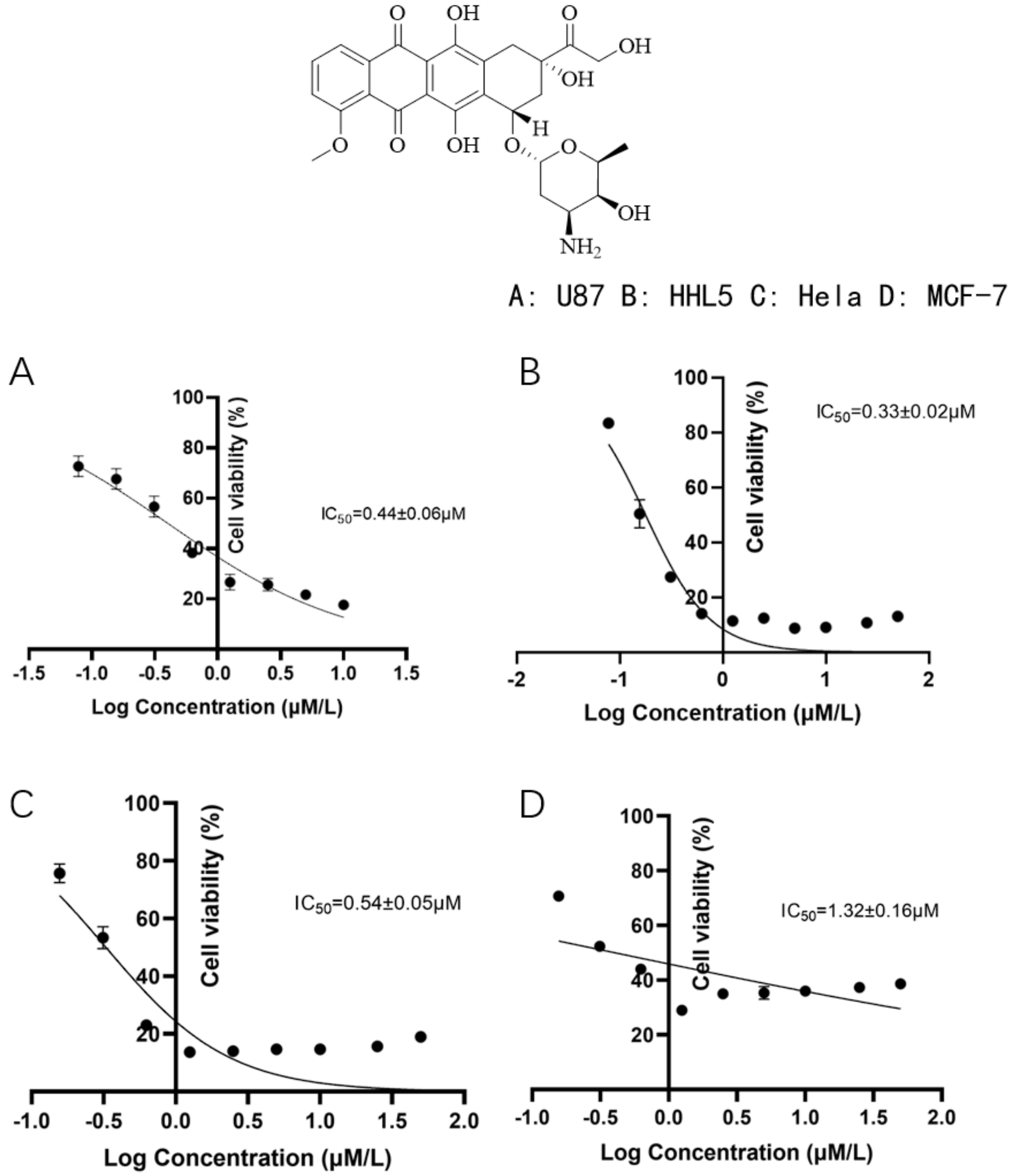

Figure S94 Dose–response curves of **Dox** on the cytotoxicity of U87-MG, Hela, MCF-7, and HHL-5 cells

6. **Origin western blots results in U87 cells treatment with 13e**

5.1 Origin western blots results of MDM4 in U87 cells treatment with 13e

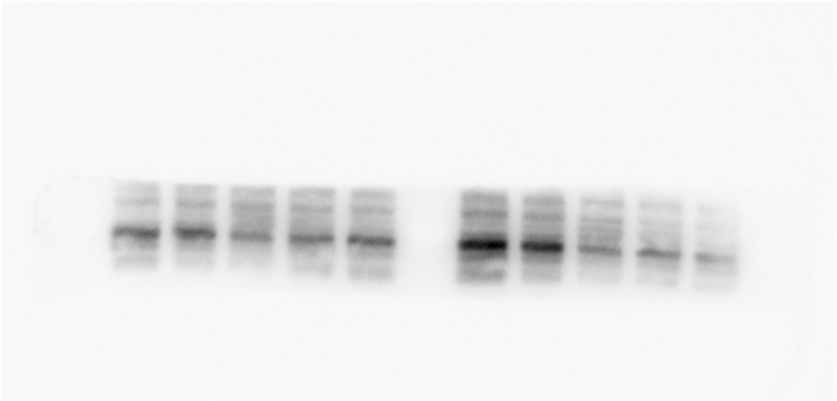

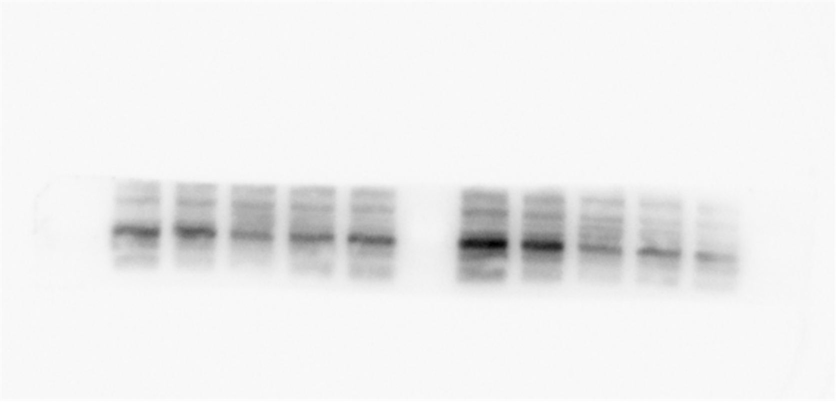

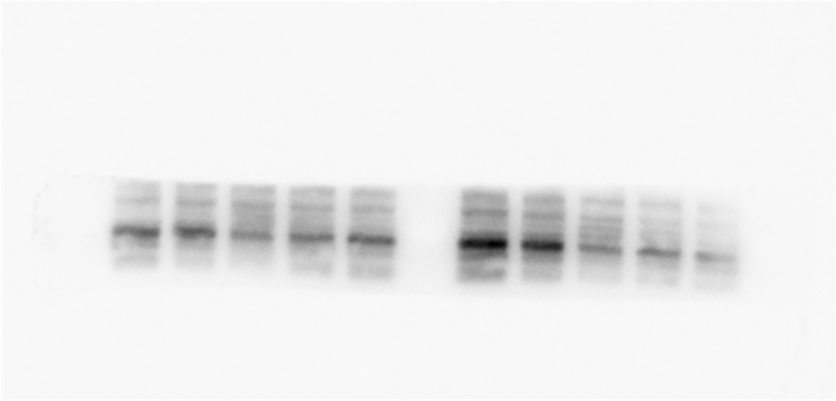

5.2 Origin western blots results of p53 in U87 cells treatment with 13e

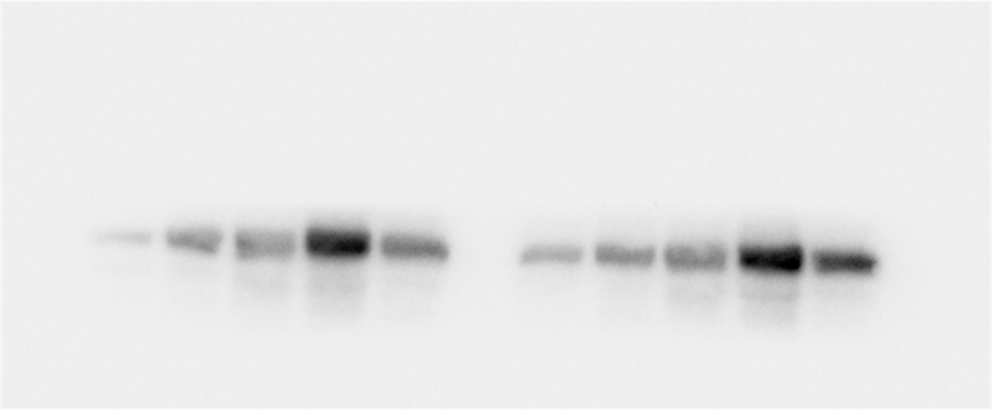

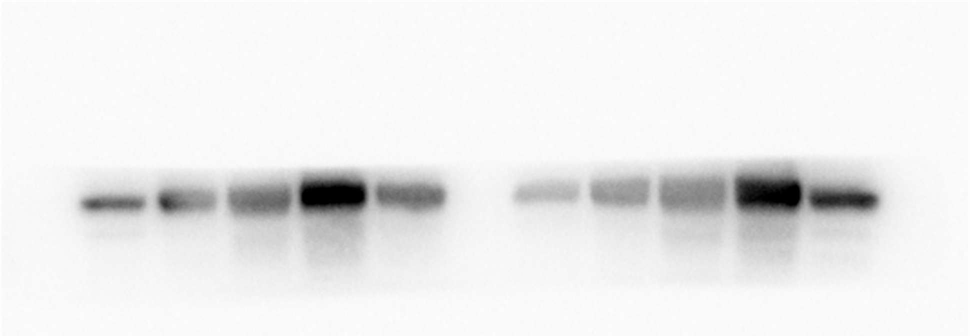

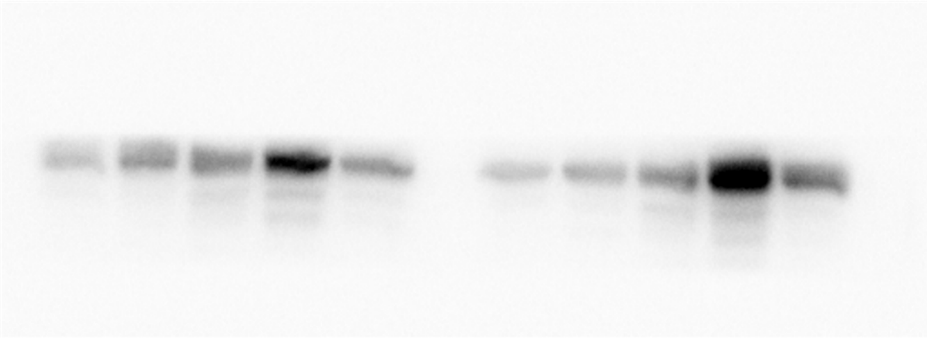

5.2 Origin western blots results of GAPDH in U87 cells treatment with 13e

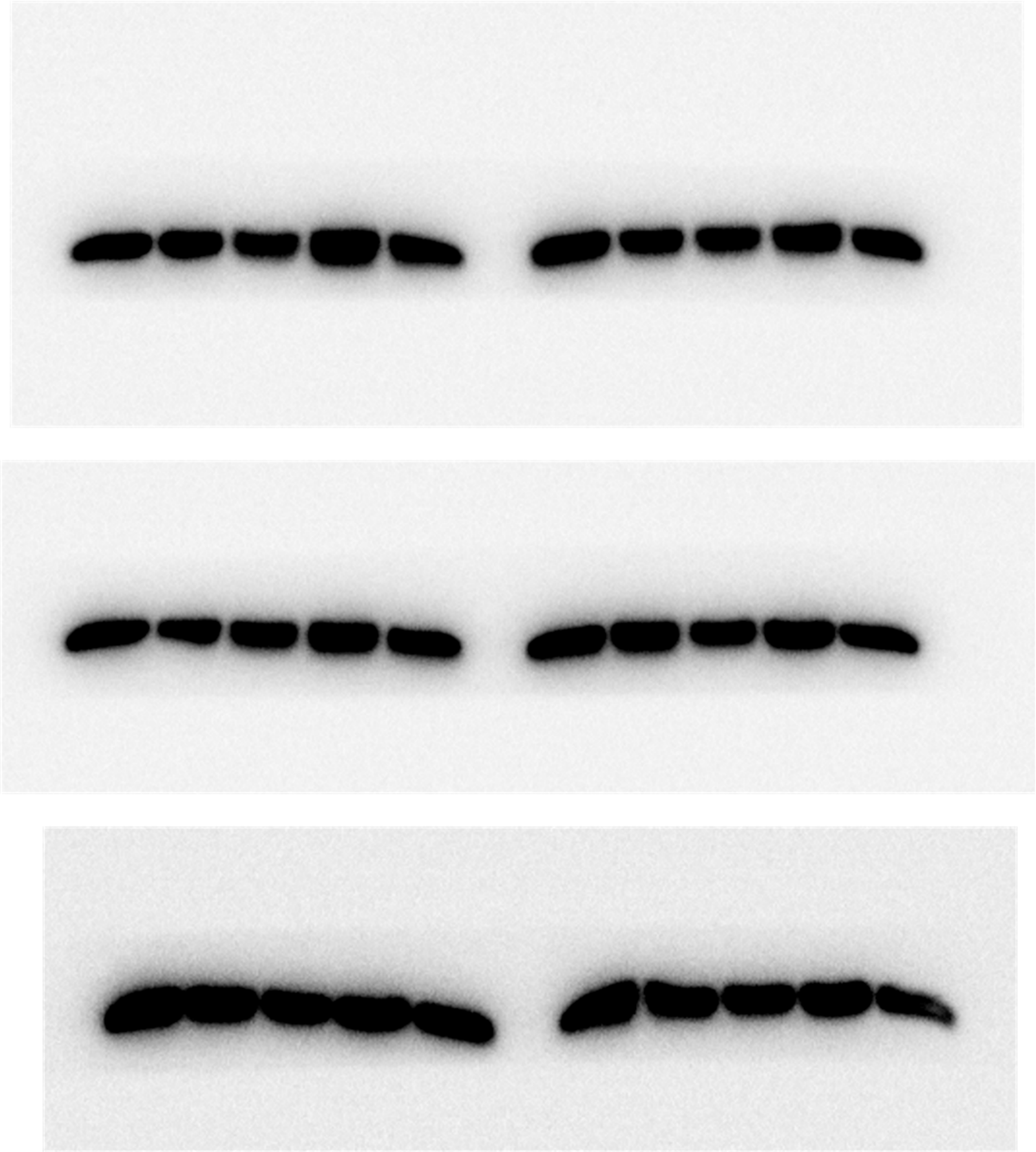

Supplement: Supplementary file 1 [file molecules-29-02396-s001.zip › molecules-2958951-supplementary.pdf]
